# Characterization of the Surfaces and Near-Surface Atmospheres of Ganymede, Europa and Callisto by JUICE

**DOI:** 10.1007/s11214-024-01089-8

**Published:** 2024-08-08

**Authors:** Federico Tosi, Thomas Roatsch, André Galli, Ernst Hauber, Alice Lucchetti, Philippa Molyneux, Katrin Stephan, Nicholas Achilleos, Francesca Bovolo, John Carter, Thibault Cavalié, Giuseppe Cimò, Emiliano D’Aversa, Klaus Gwinner, Paul Hartogh, Hans Huybrighs, Yves Langevin, Emmanuel Lellouch, Alessandra Migliorini, Pasquale Palumbo, Giuseppe Piccioni, Jeffrey J. Plaut, Frank Postberg, François Poulet, Kurt Retherford, Ladislav Rezac, Lorenz Roth, Anezina Solomonidou, Gabriel Tobie, Paolo Tortora, Cecilia Tubiana, Roland Wagner, Eva Wirström, Peter Wurz, Francesca Zambon, Marco Zannoni, Stas Barabash, Lorenzo Bruzzone, Michele Dougherty, Randy Gladstone, Leonid I. Gurvits, Hauke Hussmann, Luciano Iess, Jan-Erik Wahlund, Olivier Witasse, Claire Vallat, Rosario Lorente

**Affiliations:** 1 Istituto Nazionale di Astrofisica – Istituto di Astrofisica e Planetologia Spaziali (INAF-IAPS), Rome, Italy; 2 Institute of Planetary Research, German Aerospace Center (DLR), Berlin, Germany; 3 Physics Institute, Space Research and Planetary Sciences, University of Bern, Bern, Switzerland; 4 Istituto Nazionale di Astrofisica – Osservatorio Astronomico di Padova (INAF-OAPd), Padua, Italy; 5 Southwest Research Institute, San Antonio, TX USA; 6 Department of Physics & Astronomy, University College London, London, UK; 7 Center for Digital Society, Fondazione Bruno Kessler (FBK), Trento, Italy; 8 Institut d’Astrophysique Spatiale (IAS), CNRS/Université Paris-Saclay, Orsay, France; 9 Laboratoire d’Astrophysique de Bordeaux, Université de Bordeaux, CNRS, Pessac, France; 10 LESIA, Observatoire de Paris, Meudon, France; 11 Joint Institute for VLBI ERIC, Dwingeloo, The Netherlands; 12 Max Planck Institute for Solar System Research, Göttingen, Germany; 13 Space and Planetary Science Center, Khalifa University, Abu Dhabi, UAE; 14 School of Cosmic Physics, Dunsink Observatory, Dublin Institute for Advanced Studies (DIAS), Dublin, Ireland; 15 NASA Jet Propulsion Laboratory, Pasadena, CA USA; 16 Department of Earth Sciences, Freie Universität Berlin, Berlin, Germany; 17 Division of Space and Plasma Physics, KTH Royal Institute of Technology, Stockholm, Sweden; 18 Hellenic Space Center, Athens, Greece; 19 Laboratoire de Planétologie et Géosciences, Nantes Université, Nantes, France; 20 Department of Industrial Engineering (DIN), Università di Bologna, Forlì, Italy; 21 Chalmers University of Technology, Onsala, Sweden; 22 Swedish Institute of Space Physics, Kiruna, Sweden; 23 Dipartimento di Ingegneria e Scienza dell’Informazione, Università degli Studi di Trento, Trento, Italy; 24 Department of Physics, Imperial College London, London, UK; 25 Faculty of Aerospace Engineering, Delft University of Technology, Delft, The Netherlands; 26 Dipartimento di Ingegneria Meccanica e Aerospaziale (DIMA), Università degli Studi di Roma “La Sapienza”, Rome, Italy; 27 Swedish Institute of Space Physics, Uppsala, Sweden; 28 European Space Agency – European Space Research and Technology Centre (ESA-ESTEC), Noordwijk, The Netherlands; 29 European Space Agency – European Space Astronomy Centre (ESA-ESAC), Madrid, Spain

**Keywords:** JUICE, Icy Galilean satellites, Geology, Surface composition, Near-surface atmospheres

## Abstract

We present the state of the art on the study of surfaces and tenuous atmospheres of the icy Galilean satellites Ganymede, Europa and Callisto, from past and ongoing space exploration conducted with several spacecraft to recent telescopic observations, and we show how the ESA JUICE mission plans to explore these surfaces and atmospheres in detail with its scientific payload. The surface geology of the moons is the main evidence of their evolution and reflects the internal heating provided by tidal interactions. Surface composition is the result of endogenous and exogenous processes, with the former providing valuable information about the potential composition of shallow subsurface liquid pockets, possibly connected to deeper oceans. Finally, the icy Galilean moons have tenuous atmospheres that arise from charged particle sputtering affecting their surfaces. In the case of Europa, plumes of water vapour have also been reported, whose phenomenology at present is poorly understood and requires future close exploration. In the three main sections of the article, we discuss these topics, highlighting the key scientific objectives and investigations to be achieved by JUICE. Based on a recent predicted trajectory, we also show potential coverage maps and other examples of reference measurements. The scientific discussion and observation planning presented here are the outcome of the JUICE Working Group 2 (WG2): “*Surfaces and Near-surface Exospheres of the Satellites, dust and rings*”.

## Introduction

Working Group 2 (WG2): “*Surfaces and Near-surface Exospheres of the Satellites, dust and rings*” is one of four groups established by the JUICE Project in 2015 to provide both scientific and operation support to the Science Working Team (SWT), and to work closely with the Science Operations Center (SOC) to produce the Long-Term Science Planning. The primary goals of WG2 are to: (i) consolidate and update the science goals and requirements of JUICE concerning the surface and tenuous atmospheres of the Galilean satellites, as well as the smaller moons and the dust ring system; (ii) prepare detailed observation strategies for planning purposes; (iii) assess the science return from the different JUICE mission phases; and (iv) understand opportunities for synergistic observations between instruments.

In this article, focusing on the surfaces and tenuous atmospheres of the three icy Galilean satellites, we summarise the scientific rationale deriving from the state of the art of the observations and modelling available up to now, and we clarify which key measurements will be addressed by JUICE in the light of this rationale and of the planning discussed within WG2, considering the expected instrument performances.

Section 2 of this article summarises the general geology of the three satellites with reference to the past and present geological processes found there. We show the coverage achievable by optical imaging and we emphasise the potential of combining optical and topographical data to derive morpho-stratigraphic maps of the surface. Section 3 focuses on the surface composition of the three moons with an emphasis on the different classes of compounds known or expected on the icy Galilean satellites, and clarifying what could be obtained by a multi-wavelength analysis of remotely sensed data. A specific subsection is dedicated to the connections between the surface and subsurface (geophysical processes are discussed in detail in Van Hoolst et al. 2024, this collection), which could highlight specific areas where fresh material has risen from the interior. Section 4 focuses on the tenuous atmospheres of the three satellites, clarifying both their chemical and physical properties known to date and placing emphasis on the connections with the surface and on the potential detection of plumes. Finally, an Appendix specifies the JUICE scientific objectives and investigations relevant to Ganymede, Europa and Callisto, as defined in the Science Requirements Matrix (SRM) found in the Science Requirements Document (SciRD). These objectives are referred to in the text by means of specific codes made up by a pair of capital letters followed by a number and a lowercase letter (e.g., EA.2d, EB.1b, EC.3c, GB.1b, GC.4b, GD.1a, CA.1a, CB.1c, CC.3c, where the first capital letter refers to Europa, Ganymede and Callisto, respectively).

Given the breadth of topics covered by JUICE WG2, a separate article in this collection focuses on Io and Jupiter’s minor moons (Denk et al. 2024, this collection).

## Surface Geology of the Icy Galilean Moons (Ganymede, Callisto, Europa)

### Background

**Fig. 1:**
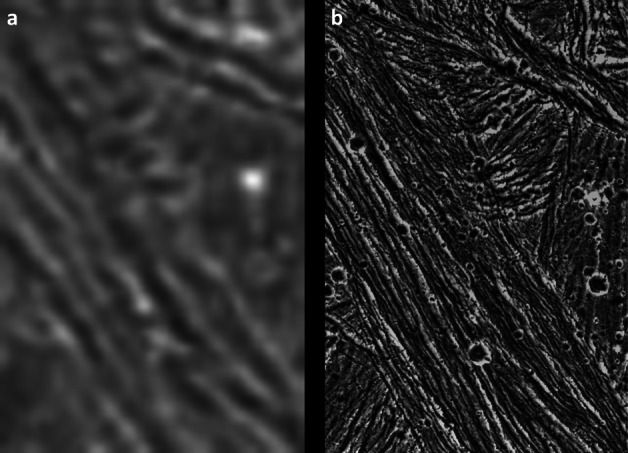
Comparison of spatial resolutions in two images obtained during the Voyager (panel a) and Galileo (panel b) explorations of Ganymede. The left and right frames have a scale of ∼1.3 km px^−1^ and 74 m px^−1^, respectively. In the left frame (panel a), high-albedo (bright) and low-albedo (dark) bands can be seen but no details can be resolved. In the right frame (panel b), with a resolution improved by a factor of 17, each band turns out to be made of many smaller ridges. In both frames north is to the top, and the Sun illuminates the surface from the lower left. The area is centred at 10°N/167°W and is about 35 × 55 km in size (Image: NASA/JPL). The reports by Kersten et al. (2021) and Hansen et al. (2024) provide information on the images of Ganymede and Europa acquired by the Voyager, Galileo, and Juno missions, respectively

The surfaces of the icy Galilean satellites have been closely explored by six NASA-led spacecraft: Pioneer 10 and 11 (1973 and 1974, respectively), Voyager 1 and 2 (1979), Galileo (1995–2003), and Juno (2016–present). The data recorded by the remote sensing instruments revealed that the surfaces are shaped by different endogenic and exogenic geological processes such as tectonism, cryovolcanism, mass wasting, and impact cratering. Thus, their surfaces became accessible for analysis through geological methods and could be compared among themselves and with those of other bodies in our Solar System (e.g., Johnson 2005; Prockter and Pappalardo 2014; Collins and Johnson 2014). With increasing spatial resolution of the measurements (Fig. 1), older ideas were abandoned and/or revised, and a better understanding of the geological evolution was gained – not only of the Galilean satellites themselves, but also of the Jovian system at large. The detection of global oceans below the surface (Khurana et al. 1998; Kivelson et al. 2000; Zimmer et al. 2000; Kivelson et al. 2002; Saur et al. 2015) and the implications for habitability (for an early discussion see Reynolds et al. 1983) increased the interest in the surfaces of the icy Galilean satellites even more, as their analysis can, e.g., reveal the mechanisms of tidal heating, constrain the thicknesses and mechanical properties of the icy shells, provide insights into possible exchange processes between the interior and space, and determine the chronology of events.

### Surface Characteristics of Ganymede, Callisto and Europa

**Fig. 2:**
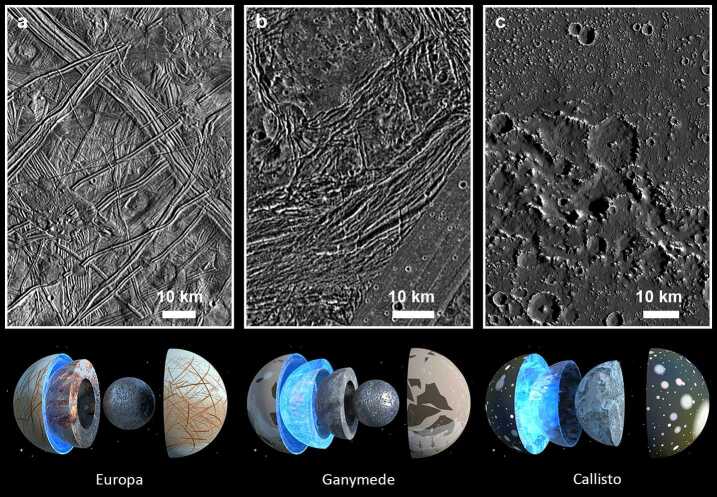
Above: Examples of typical surfaces of the icy Galilean satellites (panel a: Europa, near Conamara Chaos region at 8.1°N/90.9°E; panel b: Ganymede, Nicholson Regio at ∼14°S/12.7°E; panel c: Callisto, detail of Asgard impact basin at 14.7°N/218°E). All images are scaled to 150 m px^−1^. The crater density (a proxy of surface age) increases from Europa to Callisto, reflecting the geological history of surface processes. The surface of Europa displays the most complex inventory of landforms that were formed by endogenic processes, whereas Callisto’s surface is almost devoid of any evidence for tectonic and cryovolcanic activity (Image: NASA/DLR). Below: Models of the interiors of the icy Galilean satellites (Images: ESA/ATG Medialab). Details on their examination by JUICE are provided by Van Hoolst et al. (2024, this collection)

Some geological surface characteristics are shared among Europa, Ganymede, and Callisto. At least two of these satellites are thought to feature an icy shell above a likely (salty) liquid ocean (Ganymede may even have a stack of layered oceans, Vance et al. 2014), and all of them display evidence for bombardment by exogenic impactors (i.e., impact craters and basins). Despite their spatial proximity, however, each Galilean satellite is a world of its own. One reason for that is the amount of tidal heating that they experience. Europa, the innermost of the three, receives most tidal heating, which is reflected by the youthful appearance of its surface (less impact craters) due to ongoing resurfacing processes. Europa also has the most complex surface compared to Ganymede and Callisto in terms of tectonic deformation and possible cryovolcanic activity. Ganymede is situated between Europa and Callisto, and its surface is tectonically less complex with less and/or ambiguous evidence for cryovolcanism, while showing a higher density of impact craters pointing to an older age than Europa. Lastly, Callisto is the outermost Galilean satellite, and its surface shows the least signs of recent geological activity, it is covered by large parts of dark lag deposits and is most heavily cratered. In this regard, the surfaces of the icy Galilean satellites reflect important characteristics of their interior geodynamic activity and, in contrast to the old adage «Don’t judge a book by its cover», their sharply differing covers (i.e. surfaces) are indeed indicators of significant differences in the interiors (Fig. 2). For a general review on the geology of the icy Galilean satellites see, e.g., Stephan et al. (2013).

### Major Past and Present Geological Surface Processes

**Fig. 3:**
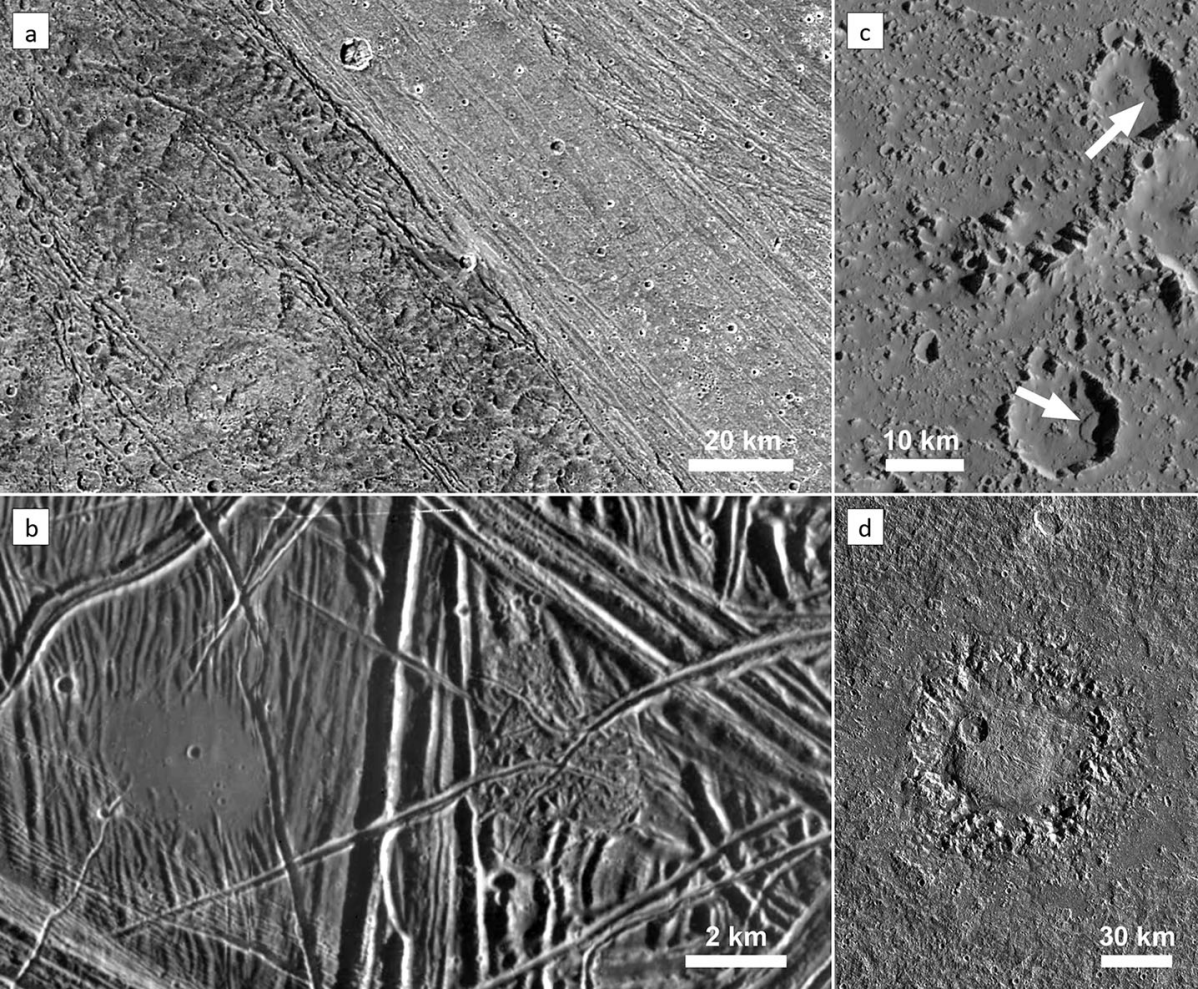
Examples of landforms created by the main four geological processes that shape the surfaces of the icy Galilean satellites. (a) Structural features in dark and bright terrains of Ganymede (Nicholson Regio and Harpagia Sulcus, respectively) are visible as linear zones of fractures. (image: NASA/JPL/DLR). (b) On Europa, a ∼3 km patch («puddle») of smooth, level terrain left of the image centre is interpreted as an area that has been flooded by an extruded liquid (water). Many tectonic fractures with different orientations and styles are crossing the entire scene (image: NASA/JPL/ASU). (c) Landslide deposits in craters on Callisto. The two landslides (arrows) are about 3 to 3.5 km long and are a result of mass wasting at the inner crater walls (image: NASA/JPL/ASU). (d) Dome crater Neith on Ganymede. The 45 km dome in the crater interior is surrounded by a concentric zone of rugged terrain, which represents a former central pit. The actual crater rim is barely visible and is located along the outer boundary of a relatively smooth, circular area. Neith is one example of a crater whose topography was strongly modified by post-impact relaxation or by the response of a weak substrate to a high-energy impact (image: NASA/JPL/DLR). North is up in all images

A review of the investigation of specific landforms and their geological implications would be beyond the scope of this section. To this end, the reader is referred to individual review studies on specific features and processes. Most, if not all landforms on the surfaces of Europa, Ganymede and Callisto can be attributed to four classes of geological processes: (1) tectonism, (2) cryovolcanism, (3) mass wasting, and (4) impact cratering (Fig. 3). Tectonic deformation of the ice shells left a diverse record of stresses and associated strain, expressed as different classes of structural features (Collins et al. 2010). They are most pristine and complex on Europa, still abundant in the bright grooved terrains of Ganymede (Fig. 3a, b), and least obvious on Callisto. The second class of endogenic landforms is represented by cryovolcanic features (Fig. 3b), which are most diverse on Europa, less numerous and certain on Ganymede, and basically absent on Callisto, consistent with the notion that tidal heating and the consequent geological activity decreases from Io to Ganymede, and does not concern Callisto (Geissler 2015; Fagents et al. 2022). Mass wasting occurs everywhere on Solar System bodies with (partly) inclined surfaces, including volatile-rich terrain (Moore et al. 1996; Melosh 2012) (Fig. 3c). Last, but not least, impact craters and basins are the most ubiquitous surface features (Fig. 3d), and the investigation of their morphologies, specifically, reveal important properties of the icy shells of the Galilean satellites and their thermal structure and evolution (Senft and Stewart 2011; Burchell 2013). A comprehensive list of landforms that are relevant to meeting the science objectives of JUICE and a selection of suggested targets is presented by Stephan et al. (2021a).

The analysis of all the respective surface features will benefit from a combination of data sets acquired by several remote instruments onboard JUICE, notably JANUS images (Palumbo et al. 2024, this collection) and GALA laser altimetry profiles (Hussmann et al. 2024, this collection). See Table 1 for a summary of the ground sampling of several JUICE remote sensing instruments for different sub-phases of the Ganymede orbital mission phase.

**Table 1:** Ground sampling / spot size of JANUS, MAJIS, UVS, GALA and SWI as a function of altitude above the surface for the three different sub-phases of the JUICE orbital mission at Ganymede, assuming nadir looking. GALA cannot operate in GCO5000. (*) The GCO200 sub-phase is not optimal for acquiring images and spectra in the ultraviolet to infrared range, due to smearing and long shadows in the observed scene, ultimately resulting in very low SNR. For JANUS, MAJIS and UVS, the salient sub-phases are GCO5000 and GCO500. SWI also does not plan to operate in GCO200, due to inability to calibrate the instrument with the spacecraft in a strict nadir-looking attitude at 200-km height

	Ganymede orbital mission phase		
	GCO5000	GCO500	GCO200*
Average altitude (km)	5100	490	200
JANUS (m)	76.5	7.4	3
MAJIS (m)	765	75	30
UVS (m)	8011	770	315
GALA (m)	-	49	20
SWI (600 GHz) (m)	10200	980	-
SWI (1200 GHz) (m)	5100	490	-

Images enable the recognition and morphologic investigation of landforms as well as the determination of model ages (Stephan et al. 2013). Stereoscopic images allow the derivation of topographic information and the construction of Digital Elevation Models (DEM). Laser altimetry profiles provide along-track shot-to-shot topography and can be interpolated along-track and cross-track to construct DEM. In cross-track, the resolution of a GALA-based terrain model is driven by the separation of neighbouring profiles. These vary over the surface of Ganymede from a few hundred metres at polar regions to a few km at the equator. The spot size corresponds to about 7 × 7 JANUS pixels and roughly the expected spatial resolution of DTM determined from stereo-pairs. Topographic information from either stereoscopic images or laser altimetry is essential for quantifying processes in all four categories of landforms and surface processes (e.g., Melosh 2012). Subsurface profiles of thermal, compositional, and structural horizons obtained through RIME (Bruzzone et al., this collection) will be very useful to combine surface and subsurface information. The analysis of the surface morphology of the icy Galilean satellites will enable addressing many of the JUICE scientific objectives (see the Appendix).

#### Tectonics

Different tectonic features exist on the surface of Europa, Ganymede and Callisto. In particular, Europa shows ubiquitous extensional deformation on its surface, such as isolated troughs, which are V-shaped depressions measuring 100-300 m wide and spanning several to hundreds of kilometres, whose formation remains uncertain. These troughs are part of a morphological progression that includes double ridges on Europa and wider ridge complexes, suggesting an evolutionary sequence. Such ridges, which are composed of raised rims to either side of a central fracture, likely formed in response to a combination of extensional, compressive, and shear-related processes. Then, dilatation bands, which are polygonal regions of smoother terrain with distinct boundaries, formed in response to both tidally driven extension and endogenic processes. For a complete review of tectonic features on the surfaces of Europa we refer to Kattenhorn and Hurford (2009).

On the other hand, Ganymede’s surface exhibits widespread brittle deformation, with furrows in low-albedo (dark) regions and grooves in high-albedo (bright) ones. Furrows, linear to curvilinear kilometre-scale troughs, offer insight into Ganymede’s early history and tectonics. Grooves, regional scale morphotectonic structures, represent brittle deformation in light terrains, indicative of fractures and faults. Proposed tectonic processes include extensional forces, like horst-and-graben normal faulting, and strike-slip kinematics. For a complete review of tectonic features on the surfaces of Ganymede we refer to Pappalardo et al. (2004). In contrast, Callisto lacks prominent tectonic features. Furrows similar to those found on Ganymede, as well ridges and scarps, are impact-related and were created in basin-forming events, such as Valhalla or Asgard (Moore et al. 2004).

The identification and characterization of structural elements is a critical component of all surface studies of the icy shells of the outer planets’ satellites. Deformation features (e.g., faults) bear a record of the strain that the ice shells experienced (e.g., Bland and McKinnon 2015), and of exchange processes between the subsurface, the surface and, if present, the atmosphere (Tobie et al. 2010). Therefore, their investigation is pivotal to better understand of the stresses acting on the satellite (e.g., tides, non-synchronous rotation, internal forces such as convection), the properties of the icy “lithosphere”, the tectonic regime (e.g., Collins et al. 2022) and, indirectly, of the nature of an underlying ocean. Despite their overwhelming importance, however, many tectonic landforms on the icy Galilean satellites are still poorly understood (Collins et al. 2010). Several remote sensing instruments onboard JUICE will contribute to address many of the key science questions related to the structural evolution of Ganymede’s icy shell, especially JANUS, GALA, 3GM, and RIME (GB.1b, GB.2b, GD.1a, GD.1b, GD.2a). In the following paragraphs, we describe key observations of the structural geology of Ganymede and how they will be used to derive information about its tectonic history and the evolution of its lithosphere. Basically, the same considerations apply to Europa (EA.2d, EA.2e, EB.1b, EB.1c), although the limited coverage of Europa during flybys implies that tectonic studies cannot be global and structural geology will have to focus on specific targets. Callisto will be observed to a larger degree than Europa, but its tectonic inventory is sparse and may be limited to impact tectonics (Collins et al. 2010).

In the following, we report the different investigations JUICE will perform at various scales. Specifically, the global, regional and local scale analyses refer to both spatial resolution and geological analysis providing a comprehensive understanding of features and processes at different spatial scales across the planetary surface. The global scale involves features or processes that stand out across the entire planetary body or a large portion of it, and which can be studied through optical observations carried out by JANUS at a few 100s metres. At a regional scale, the focus narrows down to specific areas or regions where geological or geomorphological characteristics may exhibit some level of homogeneity or distinctiveness. This scale allows for a more detailed examination of features and processes that may be unique to certain geographic regions and require an intermediate resolution of about 100 m. Finally, the local scale zooms in even further to investigate specific sites or features within a region, providing a high-resolution view of geological formations or surface characteristics. Local-scale analysis enables the identification and characterization of individual landforms, structures, or surface textures, requiring high-resolution imaging from <10 metres to a few tens of metres).

##### Global-Scale Analyses

JUICE data will significantly improve the accuracy of structural mapping and gain an understanding of global stratigraphy. At Ganymede, global colour coverage (RGB filters) at 308 m/pixel and potentially down to 77 m/pixel with the panchromatic filter will ultimately enable complete mapping of basic categories of geological surface units (i.e., light terrain, dark terrain, impacts) (Patterson et al. 2010). Global photogeological mapping at such spatial resolution will also include large-scale tectonically relevant surface units such as bright grooved terrain, chaos regions, and bands (GD.1a). Moreover, it will be possible to map linear structural elements such as furrows and (large) ridges. Although solar illumination evolves during the Ganymede orbital mission phase in such a way that the solar incidence angle increases over time for any given location observed nadir on the dayside, making the illumination increasingly grazing (for details about the JUICE mission profile, see Boutonnet et al. 2024, this collection), the dimensions of furrows (width ∼6 to 20 km, vertical relief up to 1 km; Giese et al. 1998) and (double) ridges (width 200 m to >4 km, relief up to 100 m, even more for double ridges; Nimmo et al. 2003) are sufficiently large to be identified and mapped (e.g., lineament mapping). In addition, quantitative (e.g., strain) analyses can also be performed based on such data. The analysis of global topography as provided by GALA (e.g., through DEMs) will enable assessing large-scale elevation differences, identifying structurally coherent units, and putting constraints on displacements. However, 2D imaging data will also provide quantitative information on the deformation history, as the amount of strain associated with the opening of dilatation bands can be estimated if kinematic analysis techniques are utilised. Such analysis requires the identification of offset indicators (e.g., so-called “piercing-points”; Schultz et al. 2010a) that can reliably be identified in images with moderate resolution acquired by JANUS. Another example is the determination of lateral offset along strike-slip faults, which are known to exist on both Europa and Ganymede (e.g., Tufts et al. 1999; Cameron et al. 2018, 2019). Lateral (horizontal) strain in the lithosphere will also be quantified by determination of the ellipticity of craters, which is thought to have originated from pure extension and/or simple shear (Pappalardo and Collins 2005). Indeed, on Ganymede, tectonically deformed craters can serve as markers to test the hypothesis that high-strain fault blocks can tectonically resurface pre-existing terrains (Pappalardo and Collins 2005).

##### Regional- and Local-Scale Analyses

The global-scale mapping and the kinematic analyses will be complemented by in-depth investigations of structural elements (GD.1a and GD.1b, GD.2a). The next logical step after global mapping and kinematic analyses (and building on their results) will be the close-up inspection of selected targets to reveal their genetic characteristics. Photogeological studies of sets of deformation features, such as furrows or ridge complexes, at higher resolution are required to determine their true mechanical nature (e.g., in analogy to deformation (shear) bands in terrestrial rocks as suggested by Aydin (2006). In this context, images with a scale of <100 m/px will enable studying their detailed morphology and developing models of their origin and evolution. Detailed lineament mapping over regional scales (tens to hundreds of km) will reveal cross-cutting relationships and therefore will be pivotal to acquire a robust stratigraphy (e.g., Rossi et al. 2018). Lineament analysis will also reveal characteristics of fault populations (e.g., length-frequency distributions, interaction, and linkage), which are required information for the further analysis of mechanical stratigraphy, strain partitioning, and fault growth in ice (see Schultz et al. (2010b) for a review of the value of fault population analysis for the understanding of lithospheric properties and processes). Lineament mapping will also reveal the spacing between linear tectonic elements. For example, an applied horizontal stress can induce regularly spaced folds (buckling) with a wavelength that is a function of the thickness of the elastic lithosphere (Turcotte and Schubert 2002). Although the required stresses might be too large for Ganymede (Tobie et al. 2010), other periodic spacings such as observed in grooved terrain on Ganymede (typical wavelengths of ∼3–17 km) will be readily measurable in JANUS imagery and in GALA altimetric profiles. If the periodicity in grooved terrain is indeed caused by extensional necking instabilities, the results can be used to constrain the depth to the brittle-ductile transition and the implied thermal gradients (Dombard and McKinnon 2001). Images of JANUS, complemented by altimetric (GALA) and radar sounding (RIME) data, will also help to distinguish between different models of grooved terrain formation (e.g., tilt-block faulting).

The analysis of topographic laser profiles acquired by GALA will be extremely useful to characterise the geometry of structural features on the icy Galilean satellites. In particular, the combination of laser altimetry and imaging data is a powerful tool for the mechanical analysis of deformation features (e.g., Hauber et al. 2010). Laser altimetry profiles will be co-registered with medium-resolution images to obtain topographic profiles with very high vertical accuracy that cross tectonic features (e.g., furrows) at high angles and are directly linked to spatially resolved information (necessary to interpret the morphology, which is difficult or even impossible if based on discrete laser shots alone). Topography as measured by a laser altimeter can then be used to quantify mechanical deformation and derived properties of the icy shell (e.g., flexural uplift of trough flanks; Nimmo and Pappalardo 2004; Giese et al. 2008). Ideally, such information will be coupled with subsurface profiles of structural horizons and discontinuities acquired by RIME (e.g., the brittle-ductile interface in bright terrains; Heggy et al. 2017) (GB.1a, GB.2a).

##### High-Resolution and Stereo Imaging

Even more high-resolution images are needed to provide critical insight into some aspects of the small-scale architecture of folds and faults. Although the stereo coverage of JANUS will be limited due to operation aspects, it will be sufficient to determine key properties of selected individual faults or fault populations. One example is the determination of the displacement-length scaling of faults (Schultz et al. 2006), which has rarely been realised for icy satellites. At larger fault lengths (km to tens of km) this can be accomplished by a combination of a sufficiently dense grid of laser altimetry measurements by GALA and co-registered image data from JANUS, but for smaller fault lengths (hundreds of metres to kilometres) it can only be done with gridded DEM derived from JANUS stereo images. During the Ganymede orbital phase, the best dataset will be acquired on selected regions of interest at an average altitude of 490 km above the surface (see Table 1). High-resolution optical imagery obtained in the 7-30 metres/pixel range will also enable a better understanding of how dark material is mobilised with respect to the topography of tectonic structures (Prockter et al. 2010; Rossi et al. 2023), or how low-albedo (dark) terrain changes into high-albedo (bright) terrain (which has been suggested to be the result of motion along faults). Small-scale architectural elements of faults (e.g., relay ramps) can also be examined in detail only with very high-resolution images obtained by JANUS.

#### Cryovolcanism

Cryovolcanism is defined as the «Eruption of liquid or vapour phases (with or without entrained solids) of water or other volatiles that would be frozen solid at the normal temperature of an icy satellite’s surface» (Geissler 2015). It is a widespread process in the outer Solar System and has been reported to occur in icy satellites (Europa, Ganymede, Enceladus, Titan, Triton), on Pluto and its largest satellite Charon, and possibly even on asteroids (Ceres). As cryovolcanic eruptions might deliver materials from the interior (e.g., from subsurface oceans) to the surface, they would represent direct evidence for interior-surface exchange processes and, therefore, would make potential records of habitable environments (e.g., Kargel et al. 2000) accessible for remote sensing observations and possible future in situ analysis. Nevertheless, it should be noted that a direct ascent of ocean water to the surface of Europa is unlikely due to the very high pressures that would be required (Manga and Wang 2007), and shallow subsurface sources may be more likely (Gaidos and Nimmo 2000). Regardless of the exact location of melted subsurface reservoirs, the search for and analysis of potential subsurface intrusions is among the most important science objectives of JUICE related to Europa and Ganymede (e.g., Grasset et al. 2013).

Evidence or indication for cryovolcanism comes from observations of active plumes on Triton (Soderblom et al. 1990), Enceladus (Porco et al. 2006; Spitale and Porco 2007), Europa (Roth et al. 2014a; Jia et al. 2018), and possibly on Ceres (Küppers et al. 2014). Optical imagery of the surfaces can indirectly support these findings, e.g., unusually smooth areas on Europa or “caldera-like” features on Ganymede (Schenk et al. 2001). Europa is the JUICE target with the strongest evidence for cryovolcanism, and surface evidence consists of lobate “flow-like features”, certain elliptical to circular lenticulae, «chaos» terrain, and low-lying, smooth, low-albedo surfaces (Fagents 2003). The analysis of JANUS optical images and MAJIS hyperspectral images (Poulet et al. 2024, this collection) will provide complementary information on the morphology, microtexture and composition of potential cryovolcanic features and deposits (EA.2d). GALA will acquire two topographic profiles during the two flybys, and any segments of the laser tracks that show flat and smooth terrain (as revealed by relative shot-to-shot elevation differences as well as through the analysis of within-footprint roughness; Hussmann et al. 2024, this collection) would be candidate examples of, specifically, the cryovolcanic flows (EA.2e). Ideally, RIME will acquire co-located profiles that may display supporting subsurface evidence of subsurface intrusions (e.g., basal interfaces).

Whereas early studies, based on the inspection of lower resolution images, have tentatively interpreted bright terrains on Ganymede as the consequence of cryovolcanic resurfacing (e.g., Parmentier et al. 1982; Schenk et al. 2001), later analysis of higher-resolution Galileo images has shown that possible cryovolcanic deposits on Ganymede are rare, at best (e.g., Geissler 2015). According to our current knowledge, scarp-bounded depressions that appear to be the result of collapse and roughly resemble volcanic calderas on Earth (Lucchitta 1980), are the best candidate sites to search for cryovolcanic deposits (Solomonidou et al. 2021). They are described as «flat-floored depressions surrounded by inward-scalloped walls, breached on one side and typically associated with light subdued materials» by Collins et al. (2013). Another type of potential cryovolcanic landforms are craters hosting central domes whose formation suggests a diapiric origin, whose rise began by post impact subsurface adjustments (e.g., Melkart crater, Lucchetti et al. 2023). Only very limited topographic information is available for these landforms (Giese et al. 2017). The diameters of the caldera-like landforms range from several kilometres to tens of kilometres, hence a first identification together with establishing the geological context (GD.1a), once in orbit around Ganymede, will be possible through JANUS imaging carried out at an average altitude of 5100 km above the surface. These images will also be essential to search for potential surface changes that occurred since the last look given by the Galileo mission at the caldera-like features, but also at fractures. For a better characterization of the collapse mechanism, the associated tectonic deformation (e.g., through the detection of margin-parallel tension cracks), and the ages (GD.2a), however, higher-resolution images will be necessary (resolution of a few 10s m or better), which will require spacecraft pointing for selected features. A quantitative modelling of the physical mechanisms of cryomagma ascent and eruption together with the associated surface deformation will require topographic information. GALA measurements together with co-aligned monoscopic JANUS images will be very useful, but ideally JANUS stereo images (with respective pointing requirements) will provide spatially extended Digital Elevation Models (DEM) that allow topographic mapping and modelling of the caldera areas and surroundings (GB.2f). As for Europa, RIME profiles could reveal subsurface structures associated with cryovolcanic deposits that may have originated at these caldera-like collapse depressions (GB.2a, GD.1c).

Importantly, the compositional analysis of cryovolcanic deposits will be an important objective of JUICE. Colour information coming from JANUS in different filters (Palumbo et al. 2024, this collection) will provide constraints about the composition of such features on Ganymede (GE.1e), which will be coupled with MAJIS data at coarser spatial resolution (GE.1a; see Sect. 3).

#### Mass Wasting

The presence of various types of mass movement processes and associated features have modified the surfaces of Europa, Callisto and Ganymede at a regional scale (Moore et al. 1999; Chuang and Greeley 2000). Small-scale mass movements have been detected on Europa, primarily occurring on the steep slopes of ridges and bands, and occasionally on the rims of impact craters. These movements result in deposits accumulating at the base of the slope from where they detached, forming tongue-like bulges at the front of the deposit. On Ganymede, significant sliding movements are noted on the slopes of certain recently formed impact craters. The most prevalent mass movements observed on Callisto are lobate (flow-like) features, typically resulting in alcoves and fan-like structures due to lateral spreading. These flow-like movements often mix with impact ejecta, obscuring the original topography of neighbouring regions (Parekh et al. 2023).

The study of mass wasting processes on icy satellites can improve our understanding of frictional forces (e.g., frictional heating) and, in turn, can inform about material properties (e.g., Singer et al. 2012) and contribute to the determination of the properties of the icy shell (GB.1). Mass wasting may have also contributed to concentrate lag material in dark cratered terrains (Patterson et al. 2010). Although the topographic relief of the icy Galilean satellites is relatively small on average (though increasing from Europa to Callisto, see e.g. Giese et al. 1998; Zubarev et al. 2017; Schenk et al. 2021; Schenk 2024), high-resolution imaging by JANUS will allow the identification of such mass wasting deposits, e.g. at the foot of topographic scarps such as faults (Mills et al. 2023), curved ridges, or straight segments of polygonal impact craters (e.g., Baby et al. 2024). The initial identification of mass wasting deposits in images of relatively lower spatial resolution should then be followed by high-resolution images of selected deposits, if possible, in stereo. Typically, this will require pointing geometries. Stereo coverage will enable determining the volume or the height-runout length ratio (a measure to approximate the friction coefficients for landslides), but in some cases topography information may also come from laser altimetry data (GALA) or from photoclinometry. High-resolution data from JANUS (ideally a stereo-derived DEM) can also be used to similarly investigate the effects of frictional heating on faults (Lucas 2012).

#### Impact Cratering

Impact craters and basins are among the most ubiquitous surface features on Solar System bodies with solid surfaces, and the icy Galilean satellites are no exception. Ganymede and Callisto arguably exhibit the largest variety of impact crater morphologies among the icy moons (e.g., Kirchoff et al. 2024), which range from simple bowl-shaped craters to complex craters with (1) central peaks, (2) central pits, (3) central domes, but also include (4) topographically elevated ejecta blankets (pedestals) (5) and multi-ring impact basins (Schenk et al. 2004). These different morphologies are considered to be a function of the mechanical properties of ice or the presence of liquids in the subsurface and thus mirror the target properties of the icy subsurface at the time of the impact event (Bray et al. 2012; Luttrell and Sandwell 2006; Schenk 2002). In addition, the response of the lithosphere to an initial topographic load on an icy satellite, i.e. relaxation processes, can reveal the thermal structure and its evolution after the formation of the feature. For example, the fact that large impact craters on both satellites have sharp crater rims but anomalously shallow depths, has been interpreted as the consequence of impact crater relaxation in an ice shell where viscosity decreases with depth (Parmentier and Head 1981). One important goal is to assess how impact crater morphology (complex craters with diameter larger than 25 km) can inform the analysis of the stratigraphy and evolution of Ganymede’s near-subsurface (Schenk 2002; Senft and Stewart 2011; Bjonnes et al. 2022) and the accessibility and habitability of subsurface oceans, including exchange processes between the surface and subsurface.

Furthermore, crater chains (= *catenae*) on Ganymede and Callisto, which are thought to have been formed by the impact of a body that was broken up by tidal forces into a string of smaller objects following roughly the same orbit (Schenk et al. 1996), have been included in this target category. Their investigation enables us to better define the dynamical and physical properties of the impactors in the Jovian system and their mass and size distribution.

The determination of impact crater size-frequency distributions is a vital tool for dating planetary surfaces, which is crucial for understanding the geological evolution of these bodies themselves and their context in the broader Solar System. The crater retention ages of outer solar system bodies are model-dependent, very complicated to determine and poorly constrained due to the lack of global-scale camera data coverage at sufficiently high spatial resolutions of the icy satellite surfaces. One of the most important assumptions in interpreting crater counts is the source, nature and origin of the primary projectile population(s), which is still not well constrained. Another important assumption is the rate at which these impactor populations produce craters, but also in this case research into the dynamical evolution of projectile populations is urgently needed and not yet fully addressed. Finally, scaling laws are needed to translate the crater diameters in impact projectiles. All these points make the determination of craters age on icy satellites controversial and difficult to constrain (e.g., Bottke et al. 2024). JUICE will help in revealing insight into this topic. Indeed, achieving a nearly complete coverage of Ganymede’s and Callisto’s surfaces at homogeneous spatial scales of about 100 m/pixel will be crucial in the identification of impact craters and, hence, for advancing our understanding of the projectile populations and impact scenarios within the Jovian system. As an example, determining the absolute age of the light terrain is essential for establishing the timing or period of its formation relative to Ganymede’s evolution (Baby et al. 2024).

Several remote sensing instruments will provide data that will be useful to investigate the surface morphology of impact craters. High resolution imagery (JANUS) and topography data (JANUS and GALA) are needed to characterise the different morphologies and the transitions between the crater classes in detail and to put the individual craters into a chronological context. In particular, the large impact basins (order of a few 100s km in diameter) on both satellites are a major stratigraphic marker. The latter is essential to distinguish between morphological characteristics established during and/or after the impact event (Luttrell and Sandwell 2006) on the one hand and viscous crater relaxation with time on the other hand (Bland et al. 2017; Singer et al. 2018). Subsurface information for craters and their surroundings will be provided by RIME, ideally coupled with knowledge on the topography (as for all targets observed with RIME). Complementary to the information on surface morphology, knowledge about the physical and chemical properties of the crater material will come from MAJIS, but also from UVS (Retherford et al., this collection) and SWI (Hartogh et al., this collection) (see Sect. 3).

The most essential parameter is the topography or relief of impact craters (measurement requirements for Ganymede: GB.1a, GB.1b, GB.2a-b, GD.1a, GD.1b; for Callisto: CA.1a-c, CC.1a, CC.1b, CC.1d, CC.3b, CC.3d, CC.3e; for Europa: EB.1a-c, EB.2a-c) (e.g., Bjonnes et al. 2022). The determination of the erosion state of a crater or basin (e.g., for Ganymede: GD.3a) will support the interpretation of its morphology (e.g., is the present-day relief the result of relaxation or erosion). As impact craters can – at first order – be considered as axisymmetric targets, a topographic or radar profile through the crater centre covering the surroundings, the ejecta, and the crater itself) would in most cases be sufficient (as opposed to a complete coverage of the crater and ejecta area). For larger craters, this could be realised through a series of GALA shots or a RIME radar profile in a single orbit. Accompanying such measurements, monoscopic JANUS images obtained in the same orbit would be helpful to verify that the GALA and/or RIME profile indeed cross the crater centre; moreover, images will provide essential context information.

Although impact craters of diverse morphologies are ubiquitous on both satellites and the centres of many impact craters and basins will be crossed in nadir observation geometry, specific flyby geometries and pointing are required to observe specific impact craters. Close approaches of the spacecraft will help acquire data with high spatial resolution.

### Coverage by JUICE Remote Sensing Instruments

**Fig. 4:**
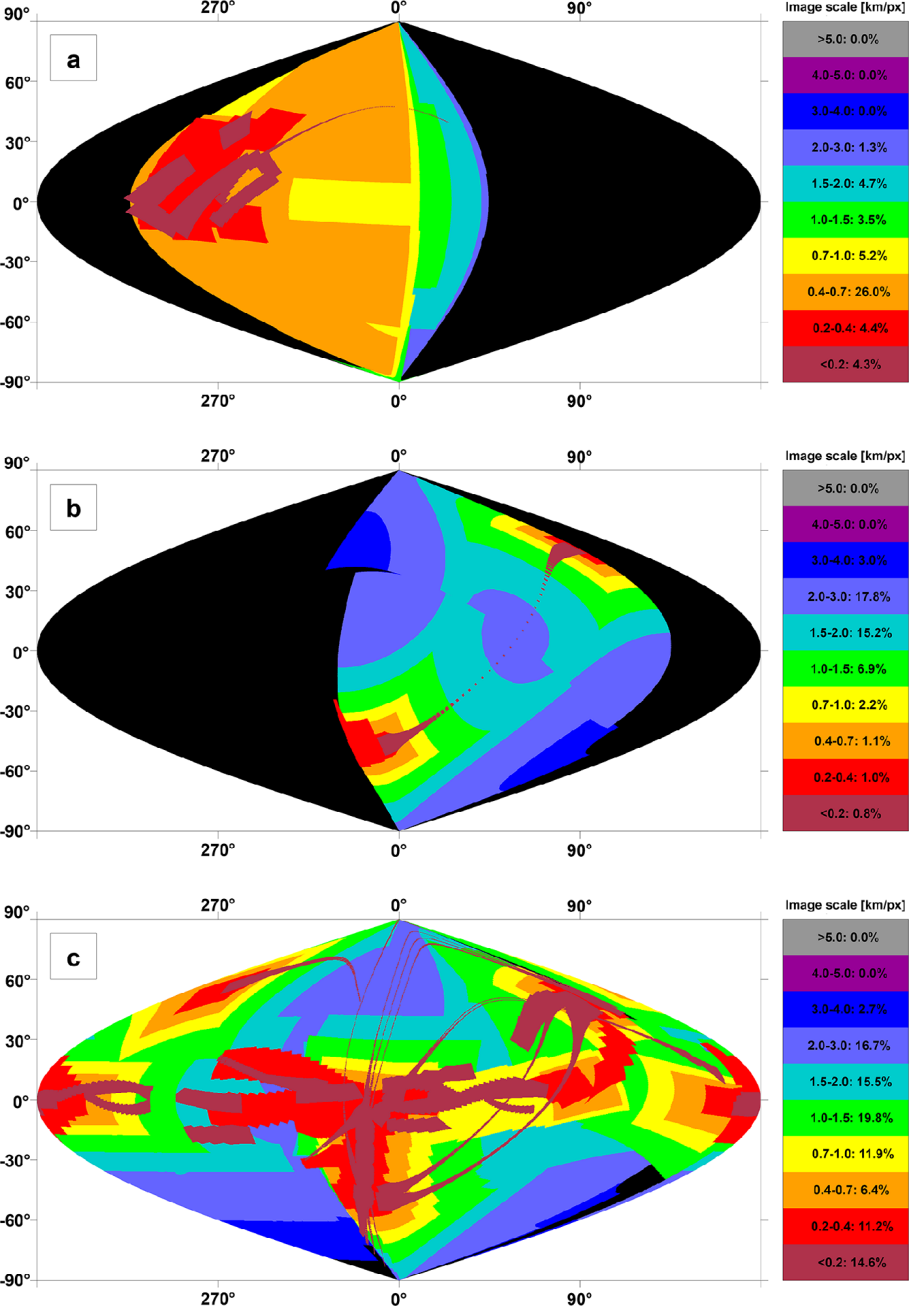
Predicted imaging coverage of the icy Galilean satellites by JANUS as obtained during flybys. (a) Europa flyby 7E1 (2 July 2032) as currently planned. The colours show the image scale, and the percentages as indicated in the colour scale bar to the right represent the surface coverage at the respective scale. The phase angle ranges between 0° and 180°, incidence angle between 0° and 70°, and emission angle between 0° and 75°. (b) Callisto flyby 18C11 (13 March 2033), under a simple assumption of nadir-looking only (no detailed planning being available at the time of writing). The phase angle ranges between 0° and 180°, incidence and emission angles between 0° and 90°. (c) Potential coverage of Callisto as obtained after all of the 21 flybys (21 June 2032 through 24 June 2034), under a simple assumption of nadir-looking only. The phase angle ranges between 0° and 180°, incidence and emission angles between 0° and 90°. All images: DLR

The JUICE mission will enable addressing the above questions using remote sensing instruments in different periods. During flybys, imaging by JANUS can be planned to obtain coverage on hemispheric scale. For example, one of the two Europa flybys (7E1) can cover a substantial part of the satellite’s surface, obviously with widely varying image resolutions (Fig. 4a). Close flybys will also allow GALA and RIME to measure profiles during the closest approach phase (<1500 km). Another example of image planning during flybys is shown in Fig. 4b for the Callisto flyby 18C11, illustrating the change of increasing and decreasing image resolution as the spacecraft approaches and leaves the satellite, respectively. Combined imaging in 21 Callisto flybys in principle could allow JANUS to achieve nearly global coverage (Fig. 4c).

**Fig. 5:**
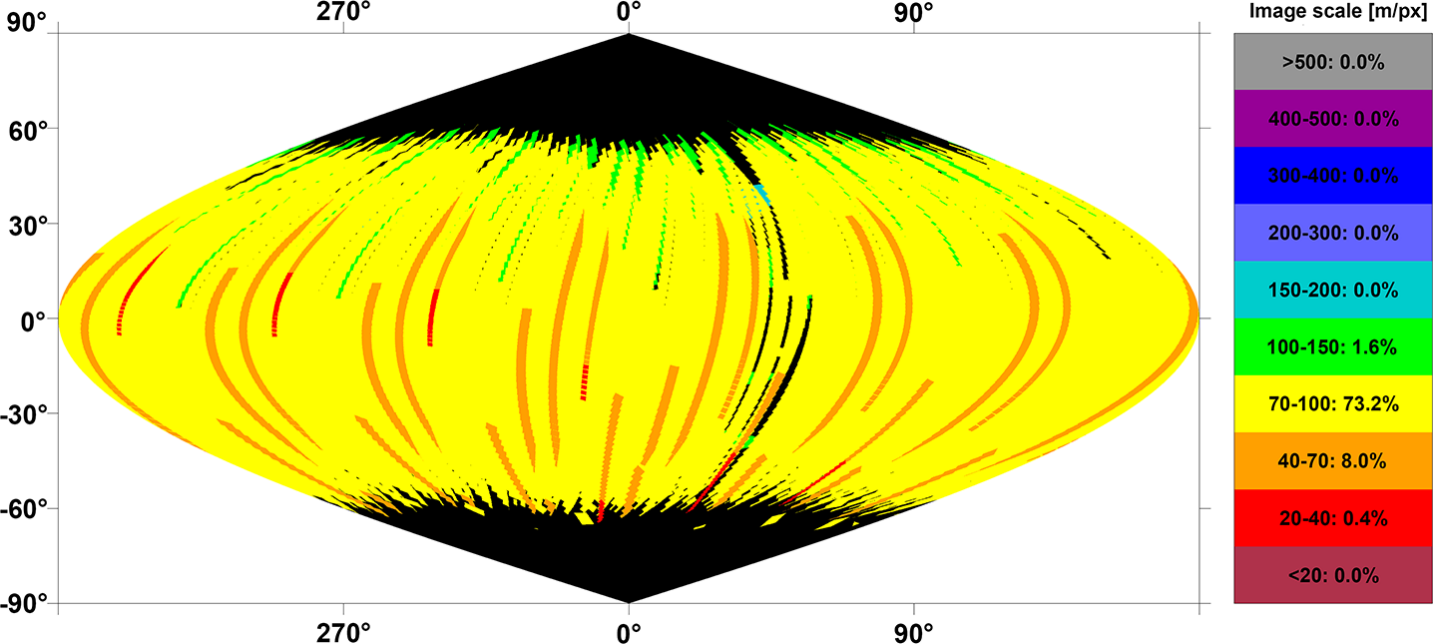
Predicted imaging coverage of Ganymede as achieved during the GCO5000 circular orbit sub-phase, carried out at an average altitude of 5100 km over the surface. Extreme illumination conditions (i.e., incidence and emission angles larger than 70° and 75°, respectively) have been filtered out. Image: DLR

**Fig. 6:**
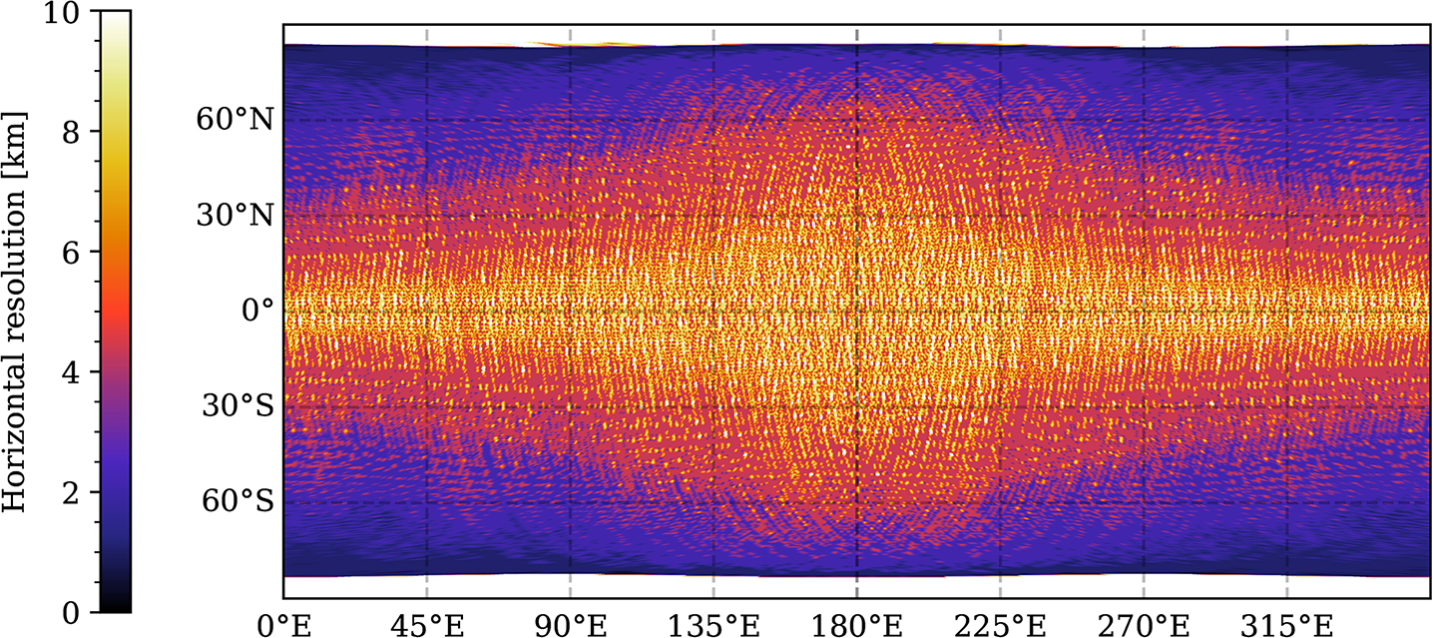
Model of predicted global topographic coverage by laser profiles acquired by GALA in GCO500. For details, see Hussmann et al. (2024, this collection)

For Ganymede, most of the imaging coverage will be obtained during the different orbital sub-phases (GEOa, GCO5000, GEOb, and GCO500, see Boutonnet et al. (2024)). Global coverage at <100 m/px will be achieved especially in the GEOa+GCO5000+GEOb period (20 December 2034 through 21 May 2035), although the solar illumination conditions will not be optimal in the polar regions and will worsen over time in the northern hemisphere (Fig. 5). This global coverage will enable: (i) identifying many of the landforms of interest, (ii) mapping their spatial distribution on a global and regional scale, (iii) establishing their relative stratigraphy by analysing, e.g., cross-cutting relationships, and (iv) determining the absolute model ages of large geological units by measuring their crater size-frequency distributions (e.g., Dones et al. 2009; Stephan et al. 2013; Wagner et al. 2014). The information collected during GCO5000 and previous mission phases will be essential to target selected regions of particular interest (Stephan et al. 2021a) at ground sampling <10 m/px in the GCO500 sub-phase, and acquire stereo image pairs for the quantitative analysis of morphometric properties of landforms. Examples of such targeted image campaigns during GC500 are shown in Stephan et al. (2021a). In GCO500, GALA will measure laser altimetry profiles and obtain global coverage (Fig. 6; for details see Hussmann et al. 2024, this collection, and Van Hoolst et al. 2024, this collection).

## Surface Composition of the Icy Galilean Moons

### Current Knowledge and Future Exploration

While on most icy satellites of the outer Solar System the composition is dominated by water ice, the presence and distribution of non-water-ice materials on the surface is pivotal for understanding the origin and evolution of the surfaces of the Galilean satellites, since the surface material can be at least partly indicative of the composition of the interior and can provide constraints on the environment in which these bodies formed and evolved. In the case of the Galilean satellites of Jupiter, most of the high-resolution composition information available to date has been collected from 1995 to 2003 by the Near-Infrared Mapping Spectrometer (NIMS) onboard the NASA Galileo spacecraft (Carlson et al. 1992), operating in the 0.7–5.2 μm range with an average spectral sampling of 25 nm beyond 1 μm and a spatial resolution varying from >100 km/px to ∼2 km/px. While the NIMS observations were a big improvement over previous ground-based spectroscopic observations, they suffered from Jupiter’s radiation environment, the instrument’s spectral resolution was low compared to present spaceborne imaging spectrometers, and spectral mapping of the satellites was achieved mostly at coarse spatial resolution (>50 km/px) due to limited downlink rate following the failure on the deployment of the spacecraft’s high-gain antenna (Taylor et al. 2002).

#### Water Ice

Since the early ground-based telescopic observations, water ice was known to dominate the surface composition of the Galilean satellites Europa, Ganymede and Callisto (Kuiper 1957; Moroz 1965). However, it was not until the Galileo spacecraft arrived at the Jupiter system that variations in the water ice properties such as abundance, grain size and crystallinity, could be observed and put in relation with surface age, geology, temperature, and radiation environment. Water ice on these bodies could be studied using Galileo/NIMS by means of diagnostic spectral signatures centred at about 0.9, 1.04, 1.25, 1.5, 2.0, and 3 μm (Grundy and Schmitt 1998; McCord et al. 1998a, 1998b). Globally, a gradient of water ice abundance could be observed throughout the surfaces of the icy Galilean satellites. While the geologically young surface of Europa exhibits the highest amount of water ice, the abundance decreases toward Callisto, whose geologically ancient surface is strongly contaminated with visually dark, non-ice materials. Ganymede’s infrared spectra suggest an intermediate global abundance of water ice with more ice in the tectonically resurfaced bright terrain and dark material concentrated in the ancient dark terrain – similar to Callisto’s surface (McCord et al. 1998a, 1998b).

Individual spectral features such as a narrow temperature-dependent band at 1.65 μm and the Fresnel reflection peak at 3.1 μm, indicate a similar trend with respect to variations in the ice crystallinity in the uppermost (<1 mm) surface layer of the icy Galilean satellites. Europa’s surface exhibits more amorphous ice, Callisto is dominated by crystalline water ice, while both types are found on Ganymede (Hansen and McCord 2004; Bockelée-Morvan et al. 2024).

The crystallinity of the surface ice is believed to reflect the radiation and temperature environment on these bodies, with radiolytic fluxes that generally increase in the trailing hemisphere and towards the polar regions. The radiation environment of Ganymede is intermediate between that of Callisto and Europa, as it has 10 times higher radiation density than Callisto, and 32 times lower than that of Europa (Cooper et al. 2001). While charged particles continuously impact the surface of Europa and destroy the structure of ice crystals, diurnal temperature variations between ∼80 and ∼165 K on Callisto (Hanel et al. 1979; Spencer 1987) cause a continuous crystallisation of the surface ice. Although Ganymede is also affected by a strong radiation environment, its surface is partly shielded from radiation due to the configuration of its unique magnetic field and influenced by similar variations in surface temperature like Callisto (Moore et al. 2004; Pappalardo et al. 2004). The global pattern of ice present on Ganymede’s surface, with amorphous ice detected in the polar regions and crystalline ice dominating the equatorial region, has been interpreted to indicate a balance between the crystallisation and disruption processes (Hansen and McCord 2004). However, grain size and temperature alone also affect the spectral signature and can add up or mimic spectral signatures of different crystallinity, which can complicate mapping physical ice properties on icy bodies (Stephan et al. 2021b). Very small ice grains (<70 μm) could explain the spectral signatures in Ganymede’s polar regions, previously interpreted as amorphous ice.

NIMS data also indicate that ice grain sizes globally vary depending on the latitude for both Ganymede and Callisto with ice grain sizes being the largest in the equatorial region and decreasing toward the poles (Stephan et al. 2020). The fact that more water ice exists on Ganymede could explain that thin polar caps could be or are formed on Ganymede and not on Callisto.

The formation of Ganymede’s polar caps has been often related to the existence and configuration of Ganymede’s magnetic field, which results in a region of closed magnetic field lines around the moon’s equator, i.e., a small magnetosphere around the satellite located within Jupiter’s magnetosphere. While the equatorial region of Ganymede is largely protected from the impacting Jovian plasma, in the polar regions the surface can easily be accessed by impacting particles causing brightening effects due to sputtering and local re-deposition of water ice molecules as fine frost (Johnson 1997; Khurana et al. 2007). This is also consistent with electron impact patterns as revealed by auroral morphology (McGrath et al. 2013; Musacchio et al. 2017). However, Callisto, which does not have an intrinsic magnetic field, shows a similar trend. The simplest explanation is that surface temperature variations likely dominate the distribution of surficial water ice on Callisto (Moore et al. 2004).

#### Non-ice Materials: Salts and Hydrates

**Fig. 7:**
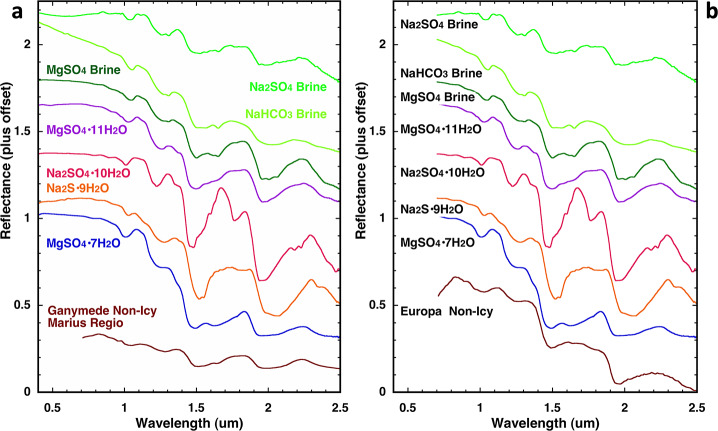
Spectra of several hydrates and brines, measured at 100 K in the range from 0.7 to 2.5 μm, compared with a NIMS spectrum of non-ice material of Ganymede’s dark terrain sampled in Marius Regio (panel a) and Europa’s non-icy terrain (panel b). Credits: J.B. Dalton

In NIMS data, non-ice materials were identified based on their spectral profiles, showing low reflectance values at wavelengths below 3 μm combined with shallow and particularly asymmetrical (distorted) water ice bands at 1.5 and 2.0 μm, as well as a relatively high reflectance in the 3–5 μm range compared to pure water ice (McCord et al. 1998a, 1998b, 1999). On Europa, these spectral characteristics are generally associated with visually dark and reddened areas, such as the *lineae* and the *chaos terrains*, and were initially interpreted as due to the presence of endogenous hydrated minerals, particularly Mg- and Na-sulphates such as hexahydrite ($\text{MgSO}_{4}\cdot 6\text{H}_{2}\text{O}$), epsomite ($\text{MgSO}_{4}\cdot 7\text{H}_{2}\text{O}$), mirabilite ($\text{Na}_{2}\text{SO}_{4}\cdot 10\text{H}_{2}\text{O}$), and bloedite ($\text{Na}_{2}\text{Mg}(\text{SO}_{4})_{2}\cdot 4\text{H}_{2}\text{O}$), which can form by crystallisation of brines erupted from below the surface and were predicted by formation models of Europa (McCord et al. 1998b, 1999, 2001a, 2001b, 2002; Dalton 2003; Dalton et al. 2005) (Fig. 7). Alternatively, it was proposed that the optically reddish material of Europa was due to hydrated sulfuric acid ($\text{H}_{2}\text{SO}_{4}\cdot n\text{H}_{2}\text{O}$), resulting from the radiolysis of water and sulphur species, or from the decomposition of sulphate salts (Carlson et al. 1999a, 1999b). Grain size and temperature affect the spectral features of hydrated salt minerals so that generally low temperatures and fine grains produce finer spectral signatures (e.g., Dalton 2003; Dalton et al. 2005; De Angelis et al. 2017, 2019, 2021, 2022). Determining the composition of dark lineaments in Europa’s trailing anti-Jovian hemisphere is non-trivial using NIMS data, which have a typical signal-to-noise ratio (SNR) ranging between 5 and 50 (Greeley et al. 2009): spectral modelling carried out between 1 and 2.5 μm reveals that water ice and hydrated sulfuric acid are essential endmembers, but it is difficult to separate hydrated sulphates from other endogenic species (Cruz Mermy et al. 2023).

In recent years, space-based and large Earth-based telescopes such as the Hubble Space Telescope (HST) and the Very Large Telescope (VLT) have been used to obtain spectra of Europa and Ganymede. Modern telescopic data have higher spectral resolution and lower noise than NIMS, and an intermediate spatial resolution of tens of km which is suited to highlight regional compositional trends. These data made it possible to identify irradiated sodium chloride (NaCl) on Europa (Trumbo et al. 2019a) and to suggest that both on Europa and on Ganymede the contribution of chlorinated salts, ultimately sourced from the interior, could be larger than other endogenous chemical species such as sulphates and carbonates (Ligier et al. 2016, 2019; King et al. 2022; King and Fletcher 2022). Recent laboratory experiments suggest that “hyperhydrated” sodium chloride hydrates such as 2NaCl⋅17H_2_O and NaCl⋅13H_2_O may form in the hydrosphere of Ganymede and possibly Europa and could be transferred to the surface through convective processes (Journaux et al. 2023). If the convective transport through the outer ice shell is efficient enough, disodium chloride decaheptahydrate (2NaCl⋅17H_2_O) is stable at ambient pressure below 235 K and may be the most abundant NaCl hydrate on the surfaces of active icy satellites (Journaux et al. 2023). The possible detection of 2NaCl⋅17H_2_O, for which infrared spectra still need to be acquired in the laboratory, may reveal areas where material recently upwelled from deep in the ice shell and ocean.

McCord et al. (2001a, 2002) and Dalton (2007) pointed out that the Europa spectra are best matched by mixtures of hydrated mineral salts and hydrated sulfuric acid in varying proportions. McCord et al. (2001a, 2002) suggested that the mechanism could be that the Na associated with some salts could be easily swept out and that abundant H^+^ could take its place, forming sulfuric acid. Shirley et al. (2010) and Dalton et al. (2012, 2013) showed that on Europa the abundance of hydrated sulfuric acid on a regional scale is dominated by the energy flux of charged magnetospheric particles. Shirley et al. (2010) also showed that the signature of the hydrated material, or dark terrain, varies according to the type of geological unit, which means that a given unit has the same relative abundances of hydrates in terms of mixtures of mineral salts and sulfuric acid (e.g., ridged plains give one set of abundances, lenticulae give another, while chaos regions have a different composition). McCord et al. (2010) confirmed this evidence, noting that the abundance of hydrates increases going from the periphery towards the centre of a given geological feature, proving that the formation mechanism seems to dominate the composition. NaCl on Europa is spatially correlated with leading-hemisphere chaos terrain, which are geologically young regions (Trumbo et al. 2022), although this portion of the satellite also displays hydrogen peroxide (H_2_O_2_), an exogenous compound resulting from radiolysis of water ice (Trumbo et al. 2019b).

On Ganymede, the surface composition as inferred from near-IR spectroscopy is similar to that suggested for Europa, which implies that also there, despite the outer ice crust being much thicker, liquid brines may have reached the surface at some time. Ganymede’s dark terrain that covers 1/3 of Ganymede’s surface is suggested to be non-ice material with endogenic origin overlying brighter icy material with the possible presence of ammonia-rich fluids in its mixture (Murchie et al. 1990; Prockter et al. 2000; Patterson et al. 2010). Ultraviolet spectroscopy data obtained during Juno’s close flyby of Ganymede in June 2021 also suggest an ammonia-like contaminant at low latitudes (Molyneux et al. 2022). Data collected by the Juno/JIRAM instrument during the same flyby at an unprecedented spatial scale of <1 km/px, covering the sub-Jovian hemisphere at low northern latitudes, revealed that Ganymede exhibits local-scale variations in the composition of geological units that were formed by different mechanisms or at very different times (Tosi et al. 2024). Mixtures of chloride salts, bloedite and possibly carbonates may be the result of extensive aqueous alteration of silicates that occurred at some point in the history of the satellite, perhaps combined with hydrothermal activity in its depths (Tosi et al. 2024).

As it was proposed for Europa, there could be an exogenous contribution produced by radiolysis and chemical reactions that take place in the first few centimetres of surface thickness. However, the pattern observed on Ganymede is substantially different due to the existence of an intrinsic magnetic field that shields the surface from the impact of electrons and heavy ions at latitudes below ∼40° (e.g., Fatemi et al. 2016; Poppe et al. 2018; Liuzzo et al. 2020; Plainaki et al. 2020a, 2020b). In this case, endogenic non-ice materials would be concentrated at low latitudes, as also suggested by the Galileo magnetometer measurements for the presence of a conductive fluid layer (McCord et al. 2001a, 2001b; Pappalardo et al. 2004), while hydrated sulfuric acid and bloedite are more abundant at high latitudes (Ligier et al. 2019; King and Fletcher 2022). Infrared spectra returned by the James Webb Space Telescope (JWST) revealed that charged-particle radiation directed by Ganymede’s intrinsic magnetic field creates H_2_O_2_ at its polar caps (Trumbo et al. 2023). Another exogenous contamination comes from the impact of micrometeorites and dust from the outer region of the Jupiter system; but there is no clear compositional distinction between the leading and trailing hemispheres as it should be in this case, while the surface composition appears dominated by the processes that define the different geological provinces.

On Callisto, a significant absorption of OH centred at 2.7 μm suggests that at least part of the dark material that mantles its surface is hydroxylated (Roush et al. 1990; Calvin and Clark 1991; Hibbitts and Hansen 2001). Also on Ganymede, a component of the non-ice, dark material might be due to phyllosilicate minerals such as smectites. Similar to hydrated mineral salts, the spectra of hydroxylated minerals are temperature-sensitive and have a different appearance at surface temperature values representative of the Galilean satellites (80–165 K) than at room temperature, showing in some cases a finer structure and sharper characteristics.

#### Non-ice Materials: Organics, Volatiles, and Clathrates

**Fig. 8:**
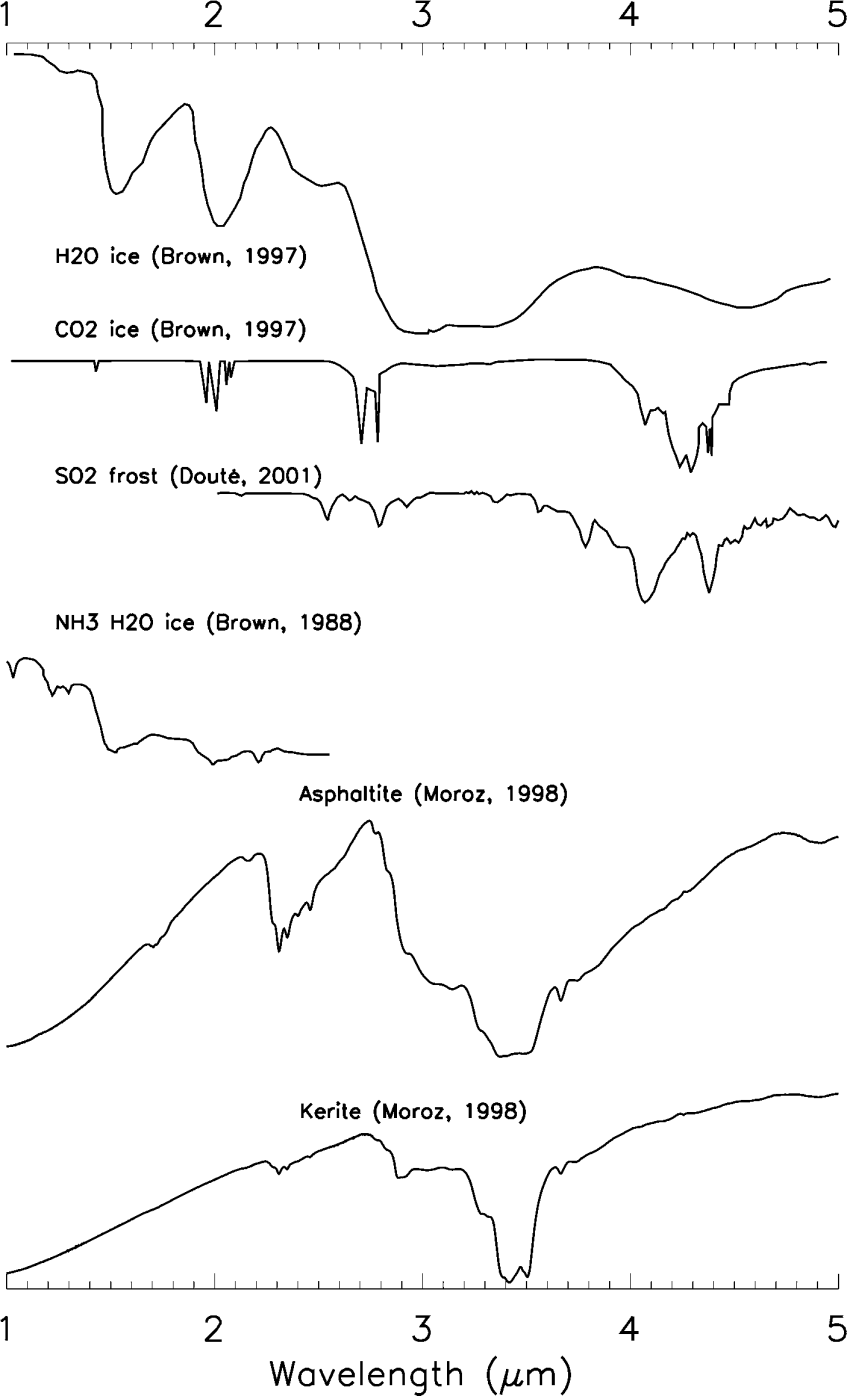
Reflectance spectral profiles of volatiles and organics relevant to the surfaces of the icy Galilean satellites, as measured in the laboratory in the near-infrared range 1–5 μm. From top to bottom: H_2_O ice (Brown and Cruikshank 1997), CO_2_ ice (Brown and Cruikshank 1997), SO_2_ frost (Douté et al. 2001), NH$_{3}\cdot $H_2_O (Brown et al. 1988), asphaltite (Moroz et al. 1998), and kerite (Moroz et al. 1998). See Poulet et al. (2024, this collection) for more spectral profiles relevant to MAJIS spectroscopic investigation

On both Ganymede and Callisto, in addition to spectral profiles attributable to mineral salts and hydrated sulfuric acid, NIMS also identified spectral signatures centred at 3.4, 3.88, 4.05, 4.26 and 4.57 μm, respectively interpreted as the spectral counterpart of the C–H bond in aliphatic organic compounds, of the S–H bond (possibly due to SO_2_–H_2_S mixtures), sulphur dioxide (SO_2_), carbon dioxide (CO_2_), and tholins, i.e. nitrogen-rich organic compounds that can form as a result of the prolonged action of charged particles on icy surfaces originally rich in organics (McCord et al. 1997, 1998a; Hibbitts et al. 2003). These features are weaker on Ganymede than on Callisto. Recent JWST/NIRSpec observations revealed that on Callisto, the absorption band at 4.57 μm is significantly stronger in the leading hemisphere unlike CO_2_, suggesting that these two spectral features are spatially anti-associated (Cartwright et al. 2024). The distribution of the 4.57-μm band is more consistent with native origin and/or accumulation of dust from Jupiter’s irregular satellites (Cartwright et al. 2024). Other weaker absorption characteristics could arise from organics containing CH, CO, carbonyl sulphide (OCS), and Na-bearing minerals. Recent observations by Juno/JIRAM also suggest the existence of aliphatic organics on Ganymede, possibly endogenic in origin (Mura et al. 2020; Tosi et al. 2024). While there is yet no reliable detection of organics on Europa, a prime candidate for exobiology, the detection, characterization and mapping of organic compounds and related products (e.g., carbonates, tholins) is a key objective of the JUICE mission (Fig. 8). In this regard, multiwavelength spectroscopy will shed light on the nature the dark material mantling Callisto and confirm whether there is any degree of contamination induced by the irregular satellites, which should concern Callisto more than Ganymede and Europa, similar to what occurs for Iapetus in the Saturn system (Tosi et al. 2010; Bottke et al. 2013).

CO_2_ of varying concentrations appears to exist everywhere on Callisto, except at high latitudes. CO_2_ is most abundant in the trailing hemisphere and in the floor, rim and ejecta of the major impact basins and impact craters, with younger craters showing larger abundance of this compound (Hibbitts et al. 2002). Impactors cannot be the source of CO_2_, as this compound sublimates rapidly at the temperatures typical of the dayside of the satellite (∼165 K at the subsolar point). Therefore, some trapping mechanisms (e.g., ice clathrates, physisorption) are thought to favour the creation of a stable underground CO_2_ deposit. JWST/NIRSpec data confirmed that the 4.25-μm band diagnostic of complexed CO_2_ is stronger at low latitudes near the apex of the trailing hemisphere, consistent with magnetospheric plasma-stimulated radiolytic production of CO_2_ (Cartwright et al. 2024). A weak absorption band of 4.38 μm probably arises from ^13^CO_2_ in the solid state. The ^13^CO_2_/^12^CO_2_ band ratios measured using Io-subtracted data indicate that carbon isotope abundances on Callisto are similar to Iapetus and other bodies that exhibit terrestrial-like values, suggesting that Callisto’s surface is not enriched in ^13^C, while a possible enhancement of ^13^C could result from delivery of irregular satellite dust (Cartwright et al. 2024).

Also based on recent JWST near-infrared observations, on Ganymede the 4.26-μm feature diagnostic of trapped or complexed CO_2_ displays variations in band centre and band shape over the leading and trailing hemispheres, which indicate that CO_2_ is present in different physical states on the surface (Bockelée-Morvan et al. 2024). However, the band depth is not correlated with Bond albedo and ice content in the equatorial regions. On the other hand, in the polar region of the leading hemisphere CO_2_ is revealed at 4.27 μm, which is consistent with CO_2_ trapped in amorphous water ice (Bockelée-Morvan et al. 2024). Unlike Callisto, impact craters are not enriched in CO_2_, whose abundance on Ganymede correlates with moderately hydrated non-ice material and appears to be dominated by local geological processes (Hibbitts et al. 2003, 2009; Tosi et al. 2023, 2024).

Unique among the Galilean satellites, Ganymede shows evidence of the presence of oxygen species trapped in the uppermost mm-thick surface layer, such as molecular oxygen (O_2_) detected at visible wavelengths (Spencer et al. 1995; Migliorini et al. 2022) and ozone (O_3_) detected in the near-UV (Noll et al. 1996). These species are most abundant in the trailing hemisphere, consistent with the preferential orientation of that side of the satellite with Jupiter’s magnetosphere, which reveals they probably arise from ionic bombardment of the ice surface. Ganymede’s Galileo/UVS measurements confirmed the presence of O_3_, whose abundance increases with increasing latitude (Hendrix et al. 1999). This has been interpreted as the result of an ozone cycle, with plasma bombardment creating O_3_ in the ice matrix and photodissociation destroying it. Furthermore, it is hypothesised that the hydrosphere of Ganymede and Callisto could host gas clathrate hydrates at temperatures ranging between 250 and 300 K (Choukroun and Grasset 2010; Journaux et al. 2020).

While Galileo/NIMS data could not safely detect CO_2_ at Europa mostly due to the coarse spectral resolution combined with very low SNR longward of 2.7 μm, Trumbo and Brown (2023) mapped the regional distribution of CO_2_ on Europa using observations obtained with JWST/NIRSpec, finding an unusual double-minima CO_2_ feature concentrated at low latitudes in Tara Regio (10°S, 75°W), a young chaos terrain, which could indicate an internal carbon source, possibly the internal ocean. Based on the same dataset, Villanueva et al. (2023) also identified this CO_2_-rich feature and measured its ^12^C/^13^C isotope ratio, confirming an internal origin.

On the surface of the icy Galilean satellites, SO_2_ is a typical exogenic compound believed to originate from implantation of sulphur ions, coming from Io, into an ice-rich surface. The presence of small amounts of SO_2_ is consistent with other exogenic species such as hydrated sulfuric acid. On Callisto, the distribution of SO_2_ is generally mottled, with some areas of high concentrations correlated with ice-rich impact craters (Hibbitts et al. 2000). Large-scale patterns include the depletion of SO_2_ in the polar regions; and a depletion of SO_2_ on the trailing side relative to the leading side is observed. High-resolution data from Galileo/UVS (Hord et al. 1992) allowed the SO_2_ absorption to be mapped out also across the surface of Europa (Hendrix et al. 1998, 2011), with a peak concentration near the apex of the trailing hemisphere. NIMS-measured SO_2_ shows that it is not strongly correlated with CO_2_ but has a similar sparse distribution (Hansen and McCord 2008). Unlike Callisto and Europa, Ganymede has a much more tenuous and time-variable SO_2_ signature (Domingue et al. 1998), likely as a consequence of the effective shielding operated by its magnetosphere against sulphur ions. In support of this hypothesis, Juno/JIRAM did not detect any SO_2_ on Ganymede at low latitudes and at the local scale (Tosi et al. 2024).

### Characterization and Mapping of Non-water-Ice Materials

In the identification and mapping of non-ice compounds, one of the most critical aspects is the resolution required to resolve the most diagnostic spectral signatures that are observed in the laboratory spectra of known or expected materials on the surface of the icy Galilean satellites, in particular hydrated materials and organics, in a broad range of spatial scales. This ability proves crucial to separate endogenous compounds, such as hydrated mineral salts, and exogenous compounds such as sulphur dioxide, hydrated sulfuric acid, and hydrogen peroxide.

The experience gained with the Galileo mission teaches that the ideal situation is when small regions of interest or specific geological formations can be observed with imaging spectroscopy at the maximum possible spatial and spectral resolution and in good conditions of solar illumination and observation, providing an adequate signal-to-noise ratio (SNR). This objective is fully complementary to the global spectral mapping obtained at a lower spatial resolution, which is needed to provide a robust compositional context.

**Fig. 9:**
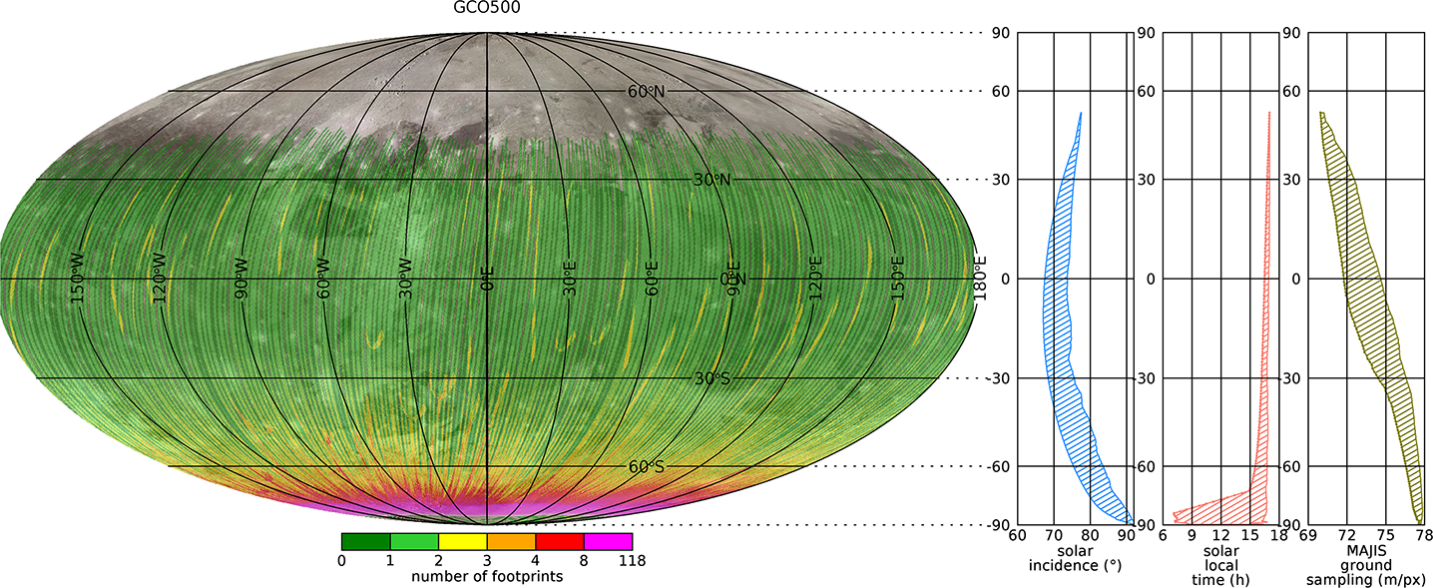
Potential coverage of Ganymede achievable by MAJIS during the circular orbital sub-phase GCO500. MAJIS footprints are counted whenever the local solar time is between 7 h and 17 h and are shown with a colour code related to the coverage redundancy (green to magenta). The right panels illustrate the corresponding coverage of solar incidence angles, local solar time and spatial resolution wrt latitude. The northern polar region will be largely in shadow by the time JUICE will enter the GCO500 circular orbit sub-phase. This estimation does not consider the available data volume; therefore, it must be understood as a maximum theoretical coverage

The mapping of the surface composition of the icy Galilean moons at different spatial scales, with particular emphasis on the detection and mapping of non-ice compounds, is the main objective of the MAJIS imaging spectrometer onboard JUICE. MAJIS operates in the overall spectral range 0.49–5.55 μm by means of two channels covering respectively the VIS-NIR interval (0.49–2.34 μm, average sampling step 3.66 ± 0.17 nm) and the IR interval (2.27–5.55 μm, average sampling step 6.47 ± 0.38 nm) (Poulet et al. 2024, this collection; Haffoud et al. 2024). At Ganymede, MAJIS will be used to record a global context, first by using flybys that have a variable spatial resolution along the trajectory, and especially during the dedicated GCO5000 orbital sub-phase. MAJIS is expected to cover at least 50% of the surface with a spatial resolution between 1 and 5 km/px (3 km/px on average) to derive compositional trends at a spatial scale that matches the best NIMS observations of Ganymede and is intermediate between the largely unexplored local scale and the regional scale already known from past NIMS and telescopic observations, highlighting in more detail both longitudinal and latitudinal gradients produced by weathering agents. During the following GCO500 orbital sub-phase, MAJIS will observe as many regions of interest as possible at the unexplored spatial resolution <100 m/px, following the order of priority dictated by both scientific relevance and by the changing solar illumination, as evidenced in the work of Stephan et al. (2021a) (Fig. 9). Correlation with detailed morphology analysis by JANUS will allow testing the origin of non-water-ice materials and distinguishing between exogenous and endogenous sources.

**Fig. 10:**
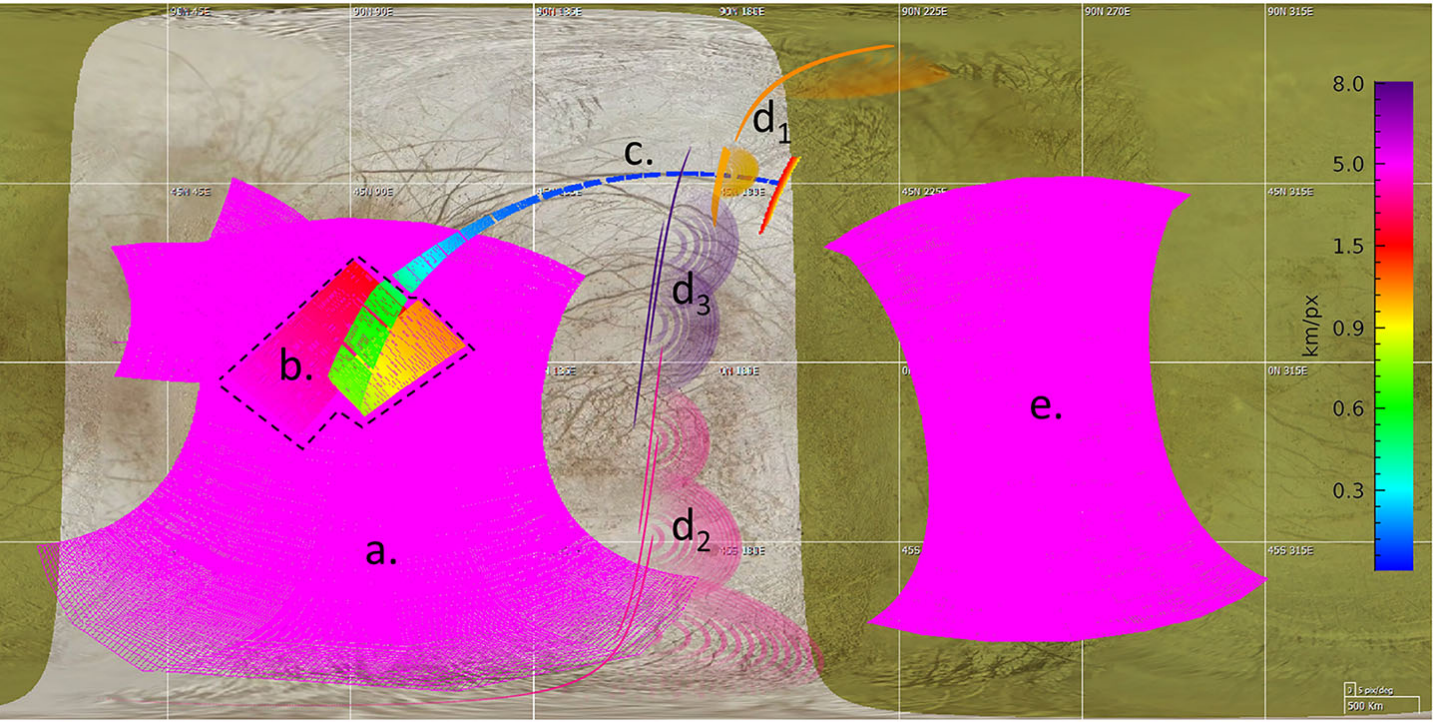
Acquisition sequence of MAJIS during the first flyby of Europa (flyby 7E1). Colours are related to the spatial resolutions achievable by MAJIS in the different observing sections, indicated by the side colour bar. Surface area shaded in dark khaki colour represents Europa’s nightside hemisphere. The observing sections composing the sequence are as follows. a. inbound dayside global scans at low-mid resolution (3–5 km/px). b. inbound dayside regional scans at mid-high res (0.5–3 km/px). c. Pushbroom dayside local scans at very high resolution (0.06–0.5 km/px). d. (1, 2, 3) outbound sequences of limb scans for the exosphere at variable resolution (1–2 km/px for d1, 5–7 km/px for d2, 8–9 km/px for d3). e. outbound nightside global scans for hotspots search at mid resolution (∼3–5 km/px)

**Fig. 11:**
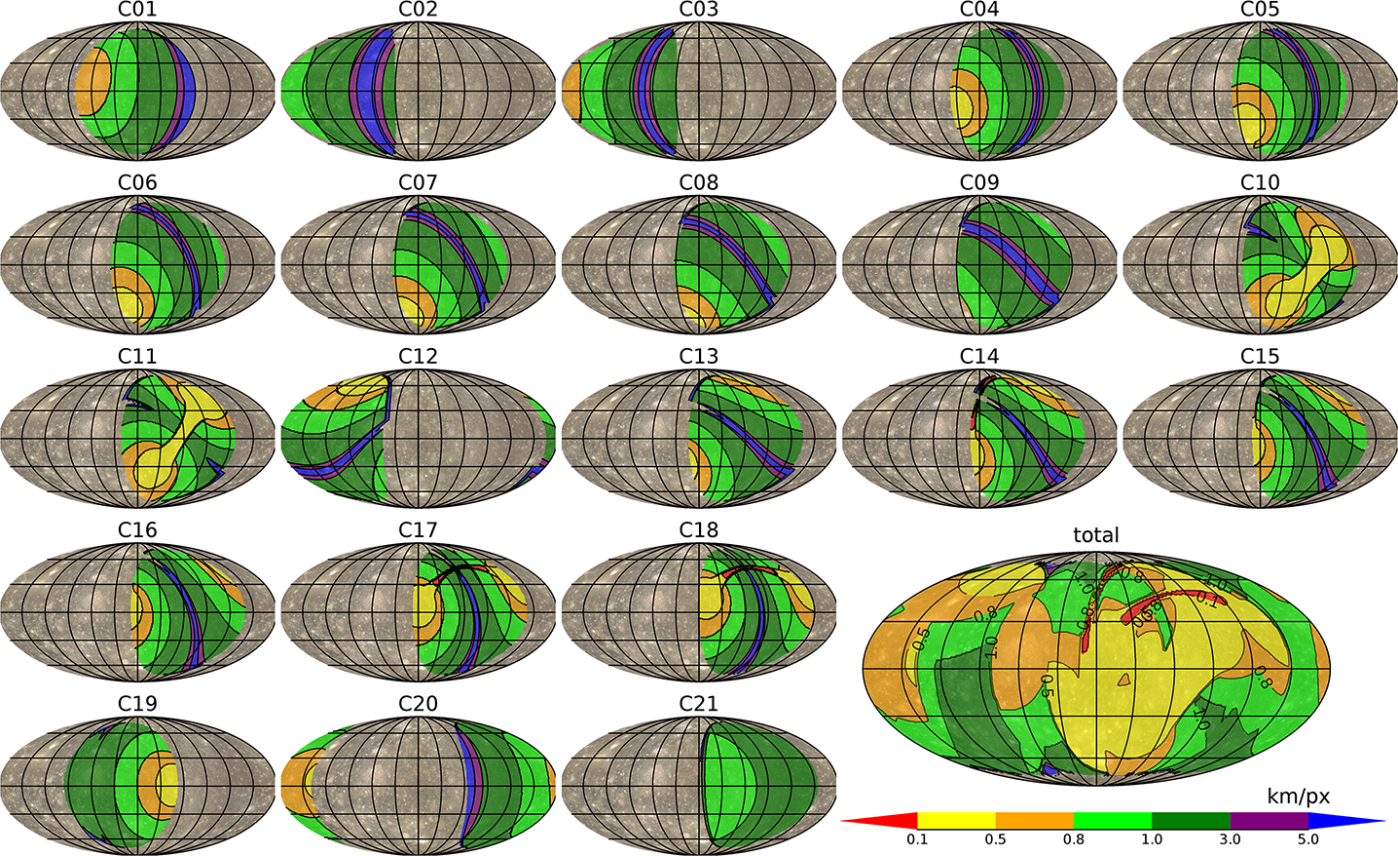
Plots of maximum diurnal coverage obtainable by MAJIS during the Callisto flybys, labelled C01 to C21, assuming a recently predicted JUICE mission profile (CReMA 5.0). Solar incidence angle is imposed <75° and viewing geometry in near-nadir direction (within 15° from nadir). The colour scale in each plot represents the best spatial resolution achievable by MAJIS. The larger map in the lower-right panel displays the potential coverage achievable by merging the coverage of all flybys with the best spatial resolution

On Europa, MAJIS will acquire medium spatial resolution data (between 3 and 5 km/px) in the inbound and outbound legs of the two flybys, mapping large areas both to characterise the largest possible number of sites of high interest and to reveal asymmetries between the leading and trailing hemispheres due to contamination from exogenous material. During the closest approach phase, MAJIS data will achieve a spatial resolution <1 km emphasising the search for organic materials, not yet identified and whose diagnostic signatures occur in the range between 3.0 and 3.7 μm, where the low reflectance of the surface creates a remarkable challenge for SNR (EA.1a, EA.2a, EA.3h, EC.3c) (Fig. 10). Callisto’s scientific objectives are similar to those for Ganymede and Europa, but with more emphasis on the distribution of volatiles (Hibbitts et al. 2000, 2002), with the aim of shedding light on the mechanisms that allow for the continuous reintegration of CO_2_ on the surface (CB.1a, CB.1c, CB.1f, CB.2c, CC.1c, CC.3c) (Fig. 11).

Mapping the distribution of volatiles across the surfaces of the icy moons is also a key goal of the JUICE UVS imaging spectrograph. Important surface materials, including O_2_, O_3_, CO_2_, SO_2_ and H_2_O_2_, have absorption features that fall partially or entirely within the 50–204 nm UVS bandpass (Retherford et al., this collection). An absorption feature at ∼180 nm, potentially related to NH_3_ and recently detected on Ganymede by Juno/UVS (Molyneux et al. 2022), will also be observable by JUICE UVS. Several organic materials additionally have distinctive spectral features in the far UV; for example, benzene absorbs strongly around 180 nm, and methane ice absorbs at wavelengths <140 nm. The presence of other non-ice materials, including tholins and silicates, may also be inferred from the observed UV spectral slope at wavelengths >170 nm (Molyneux et al. 2020). UVS will map at least 50% of Ganymede’s surface with spectral resolution ≤2 nm, to discriminate between signatures of different non-ice materials, and with spatial resolution ≤3 km, to investigate correlations with geology and/or surface processing by the Jovian magnetospheric plasma (GE.1b, GE.2f, GE.3a, GE.4c). Similar mapping will be performed across large areas of the surfaces of Callisto (CB.1b, CB.2d, CC.3c) and Europa (EA.1b, EA.2b, EA.3e, EC.3b), with spatial resolution ranging from ∼10 km, to study compositional asymmetry between the leading and trailing hemispheres, to ∼1 km or better for selected targets of highest scientific interest on Europa.

### Characterization of Physical Properties of the Surface

To spectrally characterise the physical properties of water ice, a high spectral resolution combined with good-to-adequate SNR in the spectral range 1.0–3.6 μm are mandatory. Particularly, the weak and narrow absorption bands at 1.31, 1.57 and 1.65 μm, superimposed on the deeper bands (Grundy and Schmitt 1998; Stephan et al. 2021b) as well as the reflection peak at 3.1 μm, are needed to distinguish between abundance, crystallinity, grain size, micro-porosity, and temperature variations of water ice on the surfaces of the icy Galilean satellites. The best possible knowledge of variations in these properties is also essential to correctly interpret the non-ice spectral signatures, particularly in case of hydroxylated and hydrated minerals.

The spectral characterization of the ice properties is one of the main objectives of MAJIS. While the visible spectral range, where water ice is highly transparent, can be used to characterise the abundance of water ice on their surfaces, the NIR is essential to characterise the physical properties of water ice (GC.4b, GE.1a, GE.3b, CB.1a). The spatial resolution of the MAJIS observations carried out during the GCO5000 orbital sub-phase will enable the study of the global variations in abundance, grain size, and crystallinity similar to the available Galileo/NIMS observations. However, MAJIS will provide the first global spectral coverage of Ganymede’s surface. Together with the knowledge of surface temperature and the distribution of exogenous products, the characterization of water ice will help decipher the mystery of Ganymede’s polar caps. As the north polar region of Ganymede will be in shadow during GCO500, it will be essential to observe it in previous orbital subphases to allow studying local ice properties, such as grain size and crystallinity, as a function of latitude and given local topography and geological features such as fresh impact craters (Stephan et al. 2020, 2021a). JANUS can highlight local variations in water ice abundance. In this regard, special care must be taken when patches with both strongly ice-ich and highly concentrated dark non-ice materials are close to each other and captured in the same image, resulting in a very high albedo contrast.

On Ganymede and particularly on Europa, grain size and crystallinity could also provide evidence for local surface features of recent cryovolcanic origin with possible erupted subsurface liquids. For example, on Enceladus, large crystalline ice grains have been found to dominate the ‘tiger stripes’ in the southern polar region, where ice grains crystallise from a warm liquid (Jaumann et al. 2008; Brown et al. 2006) and geysers continuously emanate smaller water ice particles and more into space (Postberg et al. 2009, 2011a).

On Callisto, MAJIS can verify the global grain size variations indicated by NIMS (Stephan et al. 2020) in comparison with Ganymede, to characterise how the competition between diurnal temperature variations and the plasma bombardment influences the surface ice properties at global and local scale. Further deciphering the local effects of temperature variations and irradiation by charged magnetospheric particles onto the surface ice of Callisto in comparison to Ganymede is essential to correctly interpret the composition and the formation and evolution of individual surface features on these bodies.

In the thermal range, diurnal temperature variations constrain thermal inertia of the surface. The 600 and 1200 GHz channels of SWI (Hartogh et al., this collection) will sense depths of about 2–8 mm (10–15 times the wavelength), yielding clues on the vertical variation of thermal inertia. In the Ganymede orbit phase, this will be achieved at spatial scales of respectively about 10 km (600 GHz) and 5 km (1200 GHz) during GCO5000, and at spatial scales of about 1 km (600 GHz) and 0.5 km (1200 GHz) during GCO500. With these two channels the emissivity properties of the regolith can also be estimated, which in turn encapsulates its physical characteristics such as porosity, grain size, and composition to some degree (GE.4b and CB.1e). In particular, the measurement of polarised thermal emission at a range of emission angles permits the separation of physical temperature and emissivity effects in producing the observed brightness temperatures, and the effective dielectric constant may be determined, bearing information on the surface and near-subsurface density and composition. The first dedicated study of the relevant inverse problem by Ilyushin and Hartogh (2020) demonstrated a reliable retrieval of the single scattering albedo and thermal skin depth from SWI radiometric measurement. Furthermore, thermophysical and dielectric properties of the surfaces will be correlated with the visible and infrared surface and albedo features. Thus, in synergy with properties constrained by other instruments, thermophysical models of the surface of the icy Galilean moons will enable to address a range of scientific questions on the (regolith) surface evolution. Finally, SWI will correlate atmospheric properties as described in Sect. 4.3 with brightness temperature maps. This will permit distinguishing between sputtering and sublimation processes, indicate potential cryovolcanic activity, and assess sublimation mechanisms at the surface or the shallow subsurface.

In the framework of the JUICE mission, quasi-specular bistatic radar (BSR) observations of Ganymede can be planned by using the 3GM radio science experiment in a downlink configuration such that JUICE is the transmitter (in either X- or Ka-band), Earth is the receiver in the specular direction of reflection, and the icy satellite is the target reflecting the radio waves.

When a polarised radio signal impacts a natural surface, reflections are altered by its statistical and electrical properties. The roughness, on an effective length-scale of a few hundreds of wavelengths, produces a frequency broadening of the echo. The terrain’s permittivity determines how the incoming polarised power is partially reflected with a same-sense and orthogonal-sense of polarisation compared to transmission. Therefore, from the reflected signal it is possible to infer physical properties of the uppermost surface layer complementary to those derived by other instruments at different wavelengths (MAJIS, GALA, RIME, SWI), such as average surface slope (roughness), near-surface dielectric constant and, with some knowledge of surface composition and porosity (Simpson et al. 1993).

The near-surface material of the Galilean moons is expected to be mostly water ice, with a various fraction of non-ice contaminants (Black et al. 2001). The relative permittivity of pure water ice is 3.1 (Thompson and Squyres 1990). Any observed deviation from that value can be explained with the presence of different surface materials, or with a different porosity or purity of the water-ice layer (Heggy et al. 2017). If specular BSR experiments are carried out at an incident angle close to the Brewster’s angle (for pure water ice roughly 60 degrees), the circular power ratio equals 1. When far from this value, a polarised component of the two will be weaker, and the computation of polarised reflected power in that sense will be more uncertain (Simpson et al. 2011). In addition, large angles of incidence are always dangerous for potential shadowing effects and for the failure of the traditional model relating surface properties to echoes’ spectra (Simpson and Tyler 1981).

Before entering the Ganymede orbital phase (20 December 2034), JUICE will perform 9 flybys of Ganymede spanning equatorial regions at observation angles that rarely get close to Brewster for water ice. The >9-month long Ganymede orbital phase is more suitable for specular BSR observations of the moon. The coverage is longitudinally complete, and spans between 60° North and South, with an incidence angle between 50° and 70° the 50% of the time.

Ground-based observations of the Galilean moons suggest that surface echoes may be dominated by an enhanced backscattering component resulting from multiple subsurface scattering mechanisms (Black et al. 2001). Alongside traditional oblique observations, BSR experiments may be also carried out in a near-backscatter geometry to derive information about the geometric structure of the regolith from features of the backscatter enhancement of the icy surface of Ganymede, such as angular width and its variation with wavelength (Hapke 1990). When in this geometry, it is possible to distinguish enhanced backscattering from ice from specular reflections thanks to their different polarisation properties (Black et al. 2001; Simpson and Tyler 1991).

For BSR experiments, ground antennas in the 64–70 m diameter range, with the ability to receive in both X- and Ka-band, would help increase the detectability of echoes from Ganymede, improving the accuracy and spatial resolution of the retrieved surface properties.

### Connections Between Surface and Subsurface Processes

The purpose of a combination of data from several remote sensing instruments, for specific regions of interest on the icy Galilean satellites, is to return a three-dimensional view of those regions, impossible to achieve from individual datasets. In the case of Europa and Ganymede, this data fusion could reveal locations where the exchange of liquid material between the shallow subsurface and the surface was more frequent and intense in the past. The potential deriving from such a multidisciplinary analysis is remarkable (e.g. Tosi et al. 2023).

The RIME ice penetrating radar is the key instrument to characterise the near-subsurface of the ice crust, detecting subsurface horizons and structures with differing dielectric constants up to a depth of a few km. RIME will provide constraints on the distribution and emplacement of subsurface materials having contrasting dielectric constants, which will be key to understanding the formation of various surface features. Combined with surface composition as derived by MAJIS and UVS, this could confirm recent activity and the possible presence of pockets of liquid water on Europa essential to habitability. RIME observations relate to different phenomena and surface and subsurface features depending on the icy moon under consideration. This prospecting will be supported by JANUS and GALA, which will provide topographic information required to properly model the off-nadir reflections (*clutter*) (see Bruzzone et al., this collection; Van Hoolst et al. 2024, this collection for more details).

At Ganymede, RIME will characterise the ice shell (GB.1, GB.2), explore the formation of surface features, search for past and present activity (GD.1, GD.2) and determine the global composition, distribution, and evolution of surface materials (GE.2). In detail, RIME will measure composition interfaces being generated by: the thermal segregation of impurities (e.g., Pappalardo and Barr 2004) and/or abrupt changes in crystal fabric (Barr and Stillman 2011) associated with relict Brittle-Ductile Transitions (BDT) within the ice shell and considered as a physical indicator of heat flow causing spatial and temporal variations in the thickness of the ice shell; the dust and insoluble impurity laden lag deposit and the solid, cleaner ice beneath it; cryovolcanic flows (Golombek and Allison 1981; Collins et al. 1998; Pappalardo et al. 2004) and/or absorption associated with subsurface cryomagmatic source regions (Schenk et al. 2001; Pappalardo et al. 2004); faulting within grooved terrain by searching for offsets horst-and-graben structures and/or domino-style tilt blocks (Golombek and Allison 1981; Collins et al. 1998); manifestation of the impact process (Schenk et al. 2004) by observing composition interfaces, structural interfaces, offsets, and distributed subsurface scatterers associated with the deposition of impact ejecta and/or impact melt; fracturing and injection of melt; and abrupt changes in crystal size and/or fabric associated with crater formation.

At Europa, RIME will contribute to determining the composition of the non-ice material especially as related to habitability, and to look for liquid water under the most active sites. Subsurface exploration will provide information about the complex geological activity of young surfaces and the relationship with a subsurface ocean (e.g., Carr et al. 1998; Greenberg et al. 1999), processes internal to the ice shell (Pappalardo et al. 1998; McKinnon 1999; Pappalardo and Barr 2004; Mitri and Showman 2005; Schmidt et al. 2011) and/or arising from tidal interactions with Jupiter (McEwen 1986; Hoppa et al. 1999a,b; Kattenhorn 2004; Hurford et al. 2005; Sotin et al. 2002; Mitri and Showman 2008). A key element to explore is the relationship between the distribution of non-ice material to geological features and processes, especially material exchange with the interior (EA.2), combining JANUS, MAJIS, UVS, and SWI observations. The search for liquid water being related to young surfaces (Collins and Nimmo 2009), RIME, in conjunction with other remote sensing instruments (JANUS, MAJIS, UVS, SWI), will determine the location of active sites and their relationship to subsurface water by detecting possible water interfaces and provide information about the rate of material exchange between the surface and the ocean as a function of the ice thickness (EB.1, EB.2, EB.3).

At Callisto, RIME will characterise the upper kilometres of the ice shell (CA.1), determine the composition of the non-ice material (CB.2) and search for past and potentially recent activity (CC.1, CC.3). Subsurface radar sounding will probe the icy shell providing a unique way to evaluate the origin and composition of the crust and the nature of the icy mantle beneath, and insights into the processes that have contributed to the formation of geological features (predominantly impacts and to a much lesser extent faulting). Subsurface sounding determines the regolith thickness at the surface and measures the depths to relict brittle-ductile transitions within the shell. It also contributes to constraining the nature and distribution of surface materials (e.g., primordial, accreted rock, or later meteoritic debris) providing scattering measurements complementary to those returned by JUICE’s spectroscopic instruments. These materials and their distributions will then be related to geological processes (e.g. impacts, see Greeley et al. 2000; Moore et al. 2004; Prockter et al. 2010), which will provide powerful synergy by extending compositional information from the surface to the ice shell via detection of composition interfaces. The RIME observations, together with JANUS-derived DEMs and GALA data, will be used to determine the formation and characteristics of tectonic and impact landforms (e.g., bombardment history and faulting characteristics); to constrain global and regional surface ages; identify and locally characterise compositional or structural interfaces, distributed subsurface scatterers, as well as offsets associated with impact facies. RIME detections of up-warped subsurface reflectors will place limits on the depths of impact excavation and post-impact deformation.

## Near-Surface Atmospheres

### Current Knowledge of the Satellites’ Atmosphere Properties from Previous Observations and Modelling

The earliest models of the atmospheres of Jupiter’s icy moons were produced long before the direct detection of any specific atmospheric species there. Motivated by a stellar occultation measurement of a ∼1 bar atmosphere at Ganymede by Carlson et al. (1973) (later found to have been an incorrect interpretation of the data), Yung and McElroy (1977) developed a photochemical model of a sublimation-driven atmosphere comprising water group species. They suggested that photolysis of water vapour and subsequent escape of hydrogen should lead to a stable oxygen atmosphere at Ganymede. A similar atmosphere was also predicted to exist at Callisto, although an oxygen atmosphere at Europa was considered less likely due to the higher albedo there inhibiting sublimation.

The importance of sputtering to the generation of the satellite atmospheres was first demonstrated through laboratory studies described by Lanzerotti et al. (1978). They found that the H_2_O partial pressure assumed by Yung and McElroy (1977) in their Ganymede models could be supported entirely by charged particle sputtering of H_2_O from Ganymede’s surface. Sputtering rates were expected to be higher at Europa and lower at Callisto. Further experiments by Johnson et al. (1981), combined with analysis of plasma ion flux measurements from within the Jovian magnetosphere by Voyager, confirmed that production of gas phase species at Jupiter’s icy moons by sputtering could dominate over sublimation, depending on the local surface temperature. Brown et al. (1982) then showed that the primary species ejected from icy surfaces are H_2_O, O_2_, and H_2_, leading to O_2_-dominated atmospheres as H_2_ readily escapes and H_2_O condenses back onto the surface.

Confirmation of O_2_ atmospheres at Europa and Ganymede was finally provided by HST in the 1990s (Hall et al. 1995, 1998). Callisto’s O_2_ atmosphere, although denser than Europa’s and Ganymede’s, was not successfully detected until 2015 (Cunningham et al. 2015). Other water group species have also been observed – H_2_O vapour at Ganymede and Europa (Roth et al. 2021; Roth 2021); H coronas at all three moons (Barth et al. 1997; Alday et al. 2017; Roth et al. 2017a, 2017b) – as well as CO_2_ at Callisto (Carlson 1999) and Na and K at Europa (Brown and Hill 1996; Brown 2001). The following subsections summarise our current understanding of the atmospheres of Europa (4.1.1), Ganymede (4.1.2), and Callisto (4.1.3).

#### Europa

Europa’s atmosphere was first observed by Hall et al. (1995) via HST Goddard High Resolution Spectrograph (HST/GHRS) observations of neutral atomic oxygen emissions at 135.6 nm and 130.4 nm. Hall et al. (1995) showed that the emissions were too bright to have been caused by resonant scattering of UV sunlight and were primarily due to electron impact excitation of an oxygen atmosphere. From the OI 135.6 nm / 130.4 nm intensity ratio of 1.9 it was inferred that the atmosphere is dominated by O_2_ since electron impact dissociative excitation of O_2_ produces an intensity ratio of >2.2 for electron temperatures (T_e_) in the range 5–500 eV (Kanik et al. 2003), while electron impact excitation of O and H_2_O lead to smaller emission ratios of <0.4 (Kanik et al. 2001) and ∼0.2 (Makarov et al. 2004), respectively. Subsequent observations by Cassini UVIS detected an extended atomic oxygen corona around Europa, in addition to the bound O_2_ atmosphere (Hansen et al. 2005).

The emission intensities measured by Hall et al. (1995) are consistent with an O_2_ column density of $(1.5 \pm 0.5) \times 10^{15}\text{ cm}^{-2}$ and a maximum O column density of $2 \times 10^{14}\text{ cm}^{-2}$. Further observations using HST/GHRS (Hall et al. 1998) and the Space Telescope Imaging Spectrograph (HST/STIS – McGrath et al. 2009; Roth et al. 2014a, 2014b) similarly indicated that O_2_ is the dominant atmospheric species at low altitudes and demonstrated that the OI 135.6 nm / 130.4 nm emission ratio is consistently lower on the orbital trailing hemisphere than on the leading hemisphere. Roth et al. (2016) performed a comprehensive analysis of STIS images obtained over 20 visits between 1999 and 2015, measuring average intensity ratios of 2.3 ± 0.3 and 1.6 ± 0.1 on the leading and trailing hemispheres, respectively. They attributed this to different atmospheric O/O_2_ mixing ratios of ∼0.05 on the trailing hemisphere and ≤0.01 on the leading hemisphere. However, further work by Roth (2021) demonstrated that the reduced 135.6 nm / 130.4 nm ratio on Europa’s trailing hemisphere cannot be fully explained by atomic oxygen but instead requires the presence of a persistent H_2_O atmosphere above the trailing hemisphere, with H_2_O/O_2_ mixing ratios between 12 and 22.

Before the inference of a persistent H_2_O atmosphere at Europa, there had been several tentative indications of transient H_2_O vapour, suggestive of active plumes. Roth et al. (2014a) observed excess oxygen 130.4 nm and hydrogen Lyman-$\alpha $ (121.6 nm) emissions above Europa’s southern limb, with no corresponding increase in the 135.6 nm oxygen emission, which is consistent with the emissions expected from electron impact dissociation of H_2_O. The observed emissions were consistent with water vapour plumes 200 km high, with line-of-sight column densities of $\sim 10^{20}\text{ m}^{-2}$. Additional plume candidates with similar column densities were identified via ultraviolet observations of Europa transiting Jupiter, during which regions of increased absorption above Europa’s limb were detected (Sparks et al. 2016, 2017), although these apparent detections may also result from statistical fluctuations (Giono et al. 2020). Attempts to detect water vapour at infrared wavelengths have proven similarly difficult, yielding only upper limits (Sparks et al. 2019) and a single tentative detection that may originate from general outgassing (Paganini et al. 2020).

Further constraints on the density and composition of water group species may be derived from measurements of absorption by Europa’s atmosphere. HST/STIS observations of Europa transiting Jupiter in 2014 and 2015 revealed the presence of an atomic hydrogen corona, which was seen to absorb Jupiter’s Lyman-$\alpha $ dayglow (Roth et al. 2017a). The observations were best fit using surface H densities of $(1.50\text{--}2.25) \times 10^{3}\text{ cm}^{-3}$ and a line-of-sight 1/$r$ profile, consistent with the results of Monte Carlo simulations by Smyth and Marconi (2006), who predicted a surface density of $\sim 2 \times 10^{3}\text{ cm}^{-3}$.

In addition to water group species, extended coronas of the minor species Na and K have been detected thanks to their bright visible emissions (Brown and Hill 1996; Brown 2001). Brown and Hill (1996) observed atomic sodium emissions at 589.592 nm and 588.995 nm extending to at least 25 R_E_ from Europa’s surface. Subsequent observations of a potassium emission line at 769.896 nm indicated that the Na/K ratio at Europa is a factor of >2.5× larger than expected if the source of the material is Io (Brown 2001), as initially suggested by Brown and Hill (1996). However, Carlson et al. (2009) note that differing sputtering yields and escape fractions for K and Na may lead to escape flux ratios that vary from the source Na/K ratio, and the endogenic or exogenic nature of alkalis in Europa’s extended corona remains an open question. The maximum column densities observed (at 5 R_E_) were $\sim 4 \times 10^{9}\text{ cm}^{-2}$ and $\sim 1.4 \times 10^{8}\text{ cm}^{-2}$ for Na and K, respectively (Brown 2001).

Models of Europa’s atmosphere are consistent with sputtering of water ice as the primary source of the neutral gases (e.g., Shematovich et al. 2005; Smyth and Marconi 2006; Plainaki et al. 2018). Molecular oxygen is expected to be the globally dominant constituent at low altitudes since sputtered H_2_O readily sticks to the surface on impact whereas O_2_ does not. Sublimation of H_2_O is generally unimportant except near the subsolar point (Smyth and Marconi 2006). More recent models accounting for Europa’s rotation and diurnal surface temperature variations predict a dawn-dusk asymmetry in Europa’s O_2_ atmosphere, with O_2_ accumulating on the dusk hemisphere (Oza et al. 2019). This result is consistent with brighter aurora near dusk local time observed by Roth et al. (2016), who note that asymmetric plasma flow could also lead to brightness asymmetries. Improved mapping of the atmospheric emissions and characterization of the local plasma environment by JUICE will address this question.

At higher altitudes, lighter H_2_ molecules dominate Europa’s atmosphere. The efficient escape of H_2_ means this species is predicted to be the dominant constituent of a toroidal cloud of neutrals distributed around Europa’s orbit (Smyth and Marconi 2006; Smith et al. 2019). Electron impact ionisation of the H_2_ neutrals within this cloud produces $\text{H}_{2}^{+}$ pickup ions, which were recently detected in the region between Europa and Ganymede by the Juno JADE instrument (Szalay et al. 2022). The observed ion density distribution can be used to estimate H_2_ loss rates from Europa, which were found to be smaller than the original Smyth and Marconi (2006) estimates but consistent with more recent models (Cassidy et al. 2013; Dols et al. 2016; Plainaki et al. 2012; Vorburger and Wurz 2018). Dissociation of O_2_ within Europa’s atmosphere by magnetospheric electron impact or ion-neutral collisions produces excited O atoms which are similarly expected to escape into the neutral cloud, but this component of the cloud has not yet been detected.

Juno performed a close flyby of Europa on 29 September 2022, during which JADE directly detected $\text{H}_{2}^{+}$ and $\text{O}_{2}^{+}$ pickup ions down to ∼1.2 R_E_, allowing atmospheric neutral densities to be inferred (Szalay et al. 2024). The derived O_2_ density is a factor of ∼2–4 lower than previous estimates based on UV observations, and the source rate of O_2_ produced within Europa’s surface can be constrained to < 12±6 kg s^−1^, at the low end of previous estimates. While the H_2_ column density of $1.8\pm 0.1\times 10^{13}\text{ cm}^{-2}$ falls within the range of previous models (Smyth and Marconi 2006; Teolis et al. 2017a; Vorburger and Wurz 2018), the observed $\text{H}_{2}^{+}$ altitude profile indicates that the neutral hydrogen atmosphere is dominated by non-thermal escaping H_2_, in contrast to the expected thermalized population.

#### Ganymede

As at Europa, water species dominate Ganymede’s exosphere: O_2_, H, and H_2_O have been directly observed through ultraviolet emission and absorption measurements (Barth et al. 1997; Hall et al. 1998; Feldman et al. 2000; Alday et al. 2017; Roth et al. 2021). The first direct detection of any atmospheric species at Ganymede was achieved by the Galileo UVS instrument, which observed Lyman-$\alpha $ emissions that gradually decreased in density with increasing altitude from the surface, consistent with an atomic hydrogen corona with maximum surface density $1.5 \times 10^{4}\text{ cm}^{-3}$ (Barth et al. 1997). The hydrogen corona was confirmed using HST/STIS observations (Feldman et al. 2000), and the surface density further constrained to the range $(5\text{--}8) \times 10^{3}\text{ cm}^{-3}$ (Alday et al. 2017). The H may be produced through dissociation of both H_2_O (Barth et al. 1997) and H_2_ (Alday et al. 2017; Marconi 2007).

Ganymede’s oxygen atmosphere was initially reported by Hall et al. (1998) using HST/GHRS measurements. The observed neutral atomic oxygen 135.6 nm emission had a double peaked spatial profile not observed at Europa, which was interpreted as evidence of two distinct emission regions near Ganymede’s poles. Images at 135.6 nm obtained using HST/STIS confirmed that Ganymede’s oxygen emissions are concentrated in two auroral ovals (Feldman et al. 2000). The observed location of the auroral ovals coincides with the position of the boundary between the open and closed field lines of Ganymede’s mini magnetosphere (McGrath et al. 2013; Greathouse et al. 2022). The observed 135.6 nm and 130.4 nm emission intensities are consistent with an O_2_ column density of $(0.3\text{--}5) \times 10^{14}\text{ cm}^{-2}$.

Ganymede exhibits a similar asymmetry between the leading and trailing hemisphere in the OI 135.6 nm / 130.4 nm intensity ratio to Europa, with smaller ratios consistently observed on the trailing hemisphere (Molyneux et al. 2018; Roth et al. 2021). This was initially interpreted as evidence of a significant atomic oxygen component (O/O_2_ ratio of ∼0.1–0.15) within the trailing hemisphere atmosphere (Molyneux et al. 2018). However, Roth et al. (2021) subsequently used disk-resolved UV images to show that the low trailing hemisphere 135.6 nm / 130.4 nm ratio is instead due to a substantial H_2_O atmosphere, with $\text{H}_{2}\text{O}/\text{O}_{2} = 22\pm 10$ on the centre of the sunlit trailing hemisphere. The intensity ratio on the leading hemisphere was also found to be reduced in the disk centre, requiring a H_2_O/O_2_ ratio of 3.5±1.5 near the subsolar point.

In contrast to Europa, where the H_2_O atmosphere may feasibly be produced by either sputtering or sublimation of surface H_2_O, Ganymede’s water vapour atmosphere is more consistent with a sublimation source, as predicted by models (Marconi 2007; Plainaki et al. 2015; Leblanc et al. 2017). Vorburger et al. (2023) showed that sublimation is the most important process to populate Ganymede’s atmosphere, and electrons impinging on the surface release H_2_ and O_2_ via radiolysis. Atomic O and H, which are the observed species in the UV spectra, are mainly added to the atmosphere through the dissociation of O_2_ and H_2_ by mostly auroral electrons. Interestingly, unpublished observations by the Herschel Space Observatory’s Heterodyne Instrument for the Far Infrared (HIFI) observed a 557 GHz signature of water on Ganymede’s leading hemisphere only (Hartogh et al. 2013), in contrast with the Roth et al. (2021) results.

Recently, optical auroral emissions of neutral oxygen at 630.0 nm, 636.4 nm, 557.7 nm, 777.74 nm and 844.46 nm were detected on Ganymede’s sub-Jovian hemisphere in eclipse (de Kleer et al. 2023). From these emissions, de Kleer et al. (2023) derive an O_2_ column density of $(3.2\text{--}4.8) \times 10^{14}\text{ cm}^{-2}$ and upper limits of $3 \times 10^{13}\text{ cm}^{-2}$ and $2 \times 10^{12}\text{ cm}^{-2}$ for O and H_2_O, respectively. The authors argue that if this low H_2_O abundance were due to the collapse of a sublimated H_2_O atmosphere in eclipse, the UV aurora would be reduced in eclipse, whereas the UV emission intensity has been observed to remain similar in sunlight and shadow (Roth et al. 2021); they suggest that an alternative explanation for the low 135.6 nm / 130.4 nm emission ratio may be required. However, Roth et al. (2021) show that the H_2_O component of Ganymede’s atmosphere contributes a negligible fraction of the disk-averaged 135.6 nm emission and only ∼15% of the 130.4 nm emission, which is on the order of the measurement uncertainty, and therefore any change in the UV oxygen emissions in eclipse would be difficult to identify in current datasets. JUICE will observe the UV emissions on both the dayside and nightside of Ganymede and provide in situ measurements of key atmospheric species including H_2_O and O_2_, constraining the relative contributions of sublimation and sputtering to the generation of the atmosphere.

While observations of atmospheric emissions can place constraints on atmospheric composition and provide some insight into expected variability, modelling work is required to understand the global distribution and temporal evolution of the atmosphere. In general, models agree that O_2_ is globally abundant near the surface, with a scale height of a few tens of km, since O_2_ is heavy enough to be gravitationally bound and does not condense at Ganymede’s surface temperatures. At higher altitudes, lighter H_2_ molecules are the primary constituent. Near the subsolar point, H_2_O dominates; the atmosphere is collisional or quasi-collisional in this region at low altitudes (<50 km) and collisionless elsewhere (Marconi 2007; Shematovich 2016; Leblanc et al. 2017). Models of the varying interaction between the magnetospheres of Ganymede and Jupiter over Jupiter’s ∼10-hour rotation period (e.g. Carnielli et al. 2020; Liuzzo et al. 2020; Plainaki et al. 2015, 2020a; Poppe et al. 2018) are essential for understanding which surface regions are accessible to charged particles capable of contributing to the sputter-generated fraction of the atmosphere. These simulations show that the trailing hemisphere is shielded by Ganymede’s intrinsic magnetic field, whereas energetic ions can access the surface at low latitudes on the leading hemisphere and in regions of open field lines in the polar caps (Khurana et al. 2007). These variations in charged particle access to the surface, along with changes in surface temperature over Ganymede’s orbital period, are predicted to result in atmospheric asymmetries, including a dusk/dawn asymmetry in O_2_ (Leblanc et al. 2017; Oza et al. 2018), which will be detectable by JUICE if present.

#### Callisto

Callisto is expected to possess the most substantial atmosphere of Jupiter’s icy moons, but direct detection of water group species has proven difficult. The earliest atmospheric measurement at Callisto was instead the detection of CO_2_ by the Galileo NIMS instrument. Carlson (1999) reported a NIMS observation of infrared limb emission in the 4.26 μm band of CO_2_ up to 100 km altitude above the surface, estimating a vertical CO_2_ column density of $\sim 8 \times 10^{14}\text{ cm}^{-2}$. This CO_2_ atmospheric profile could be explained by sublimation of water ice with trapped CO_2_ (Vorburger et al. 2015). Recent JWST/NIRSpec observations have detected CO_2_ vibrational emission lines between 4.2 and 4.3 μm on both hemispheres of Callisto (for the first time on the trailing side), confirming the global presence of CO_2_ gas in the atmosphere. The distribution of CO_2_ gas is offset from the subsolar region in both hemispheres, suggesting that sputtering, radiolysis and geological processes help sustain Callisto’s atmosphere (Cartwright et al. 2024). The Galileo Callisto flybys also detected a dense ionosphere, which Kliore et al. (2002) argued to be consistent with the presence of an O_2_ atmosphere with column density $\sim 3 \times 10^{16}\text{ cm}^{-2}$ – approximately 100× denser than the O_2_ atmospheres of Ganymede and Europa.

Strobel et al. (2002) attempted to detect UV OI (130.4 nm and 135.6 nm), CI (156.1 nm), CII (133.5 nm) and CO fourth positive band emissions at Callisto using the same HST/STIS observation mode successfully used to map the 130.4 nm and 135.6 nm emissions at Ganymede and Europa but found no emissions above the 15 R detection limit. The lack of UV emissions was interpreted as a result of a strong electromagnetic interaction with Jupiter’s magnetosphere driving $\sim 1.5 \times 10^{5}$ A through Callisto’s ionosphere. Strobel et al. (2002) demonstrated that this interaction reduces the electron impact emission rate by a factor of ∼1500.

A more recent analysis of the Callisto STIS images found faint Lyman-$\alpha $ emissions consistent with a hydrogen corona with vertical column density in the range $(6\text{--}12) \times 10^{12}\text{ cm}^{-2}$ extending several Callisto radii from the surface (Roth et al. 2017b). The inferred hydrogen abundance was ∼2× higher when Callisto’s leading hemisphere was illuminated than when the trailing hemisphere was in sunlight, which was interpreted as due to increased H_2_O sublimation on the visibly darker leading hemisphere. However, similar observations of Ganymede’s hydrogen corona by Alday et al. (2017) found no hemispheric asymmetry there, despite the increased concentration of sublimated H_2_O on Ganymede’s trailing hemisphere (Roth et al. 2021). Direct Simulation Monte Carlo (DSMC) models by Carberry Mogan et al. (2022) show that the observed structure of Callisto’s hydrogen corona may be more consistent with radiolytically produced H_2_ as a primary source, suggesting that the role of H_2_ versus H_2_O in the Europa and Ganymede coronae should also be re-examined.

In 2015, the presence of an O_2_ atmosphere at Callisto was finally deduced from observations of the UV atomic oxygen multiplets using the HST/COS instrument. Cunningham et al. (2015) observed faint 130.4 nm and 135.6 nm emissions of atomic oxygen on the leading hemisphere and constrained the contributions from electron impact dissociation of O_2_ and resonance scattering of solar 130.4 nm. The observed intensities were consistent with excitation by electrons from solar photoionization rather than the Jovian magnetospheric electrons that produce the emissions at Ganymede and Europa. From the UV emission intensities, Cunningham et al. (2015) derived a leading hemisphere O_2_ column density of $\sim 4 \times 10^{15}\text{ cm}^{-2}$ and suggested that a denser O_2_ atmosphere may exist on the trailing hemisphere. Recent detections of OI 630.0 nm and 636.4 nm emissions on Callisto’s sub-Jovian hemisphere in eclipse are similarly consistent with an O_2_ column density of $(4.0\pm 0.9) \times 10^{15}\text{ cm}^{-2}$ (de Kleer et al. 2023).

**Fig. 12:**
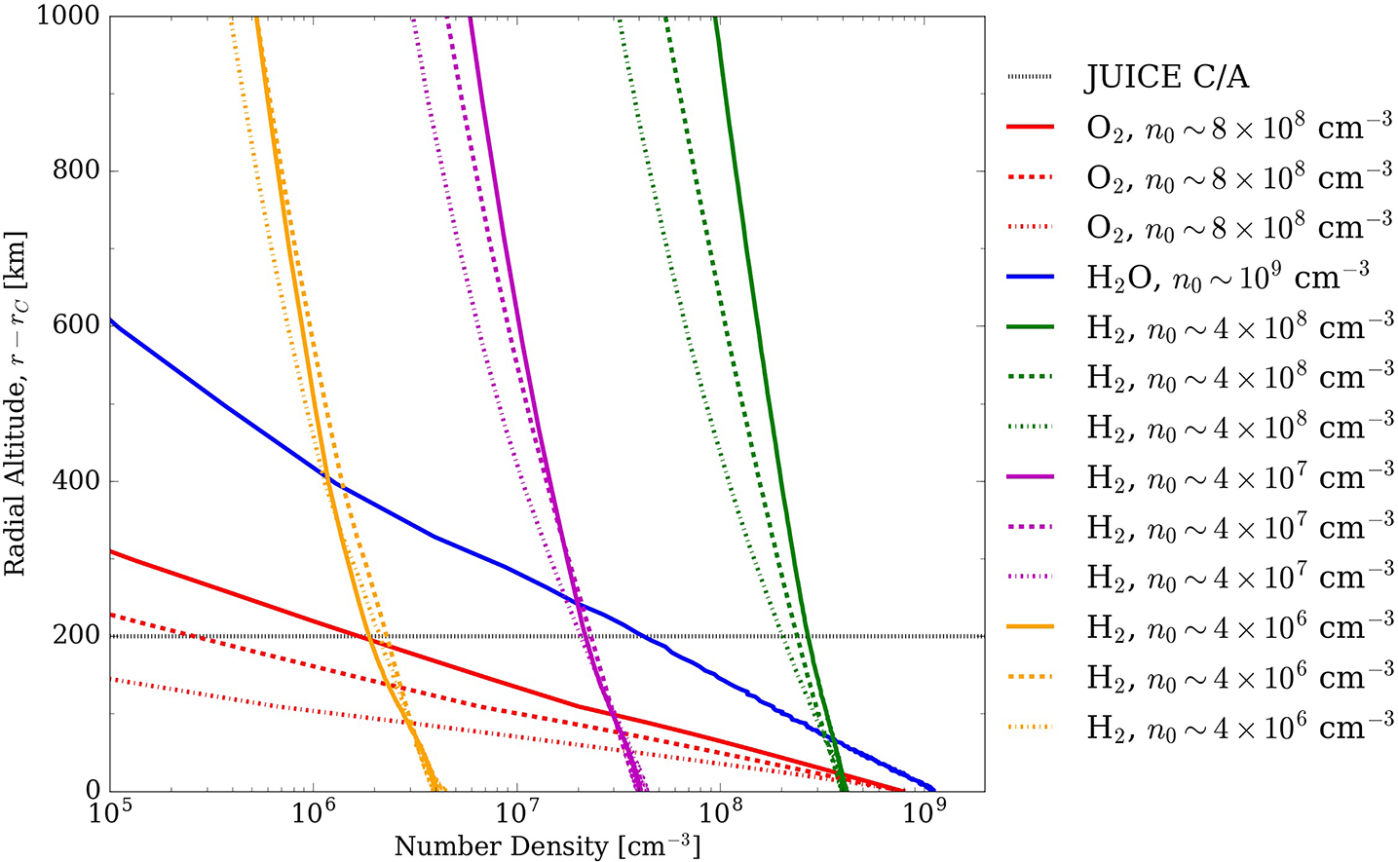
Predicted height profiles of densities for sublimated H_2_O (blue) and radiolytically produced O_2_ (red) and H_2_ (green, magenta, orange) at Callisto for various solar zenith angles (solid lines: 0°, dashed lines: 90°, dotted lines: 180°). Figure taken from Galli et al. (2022), based on Carberry Mogan et al. (2021)

Due to the difficulties in directly observing Callisto’s atmosphere, absolute column densities remain somewhat controversial (see recent publications by Galli et al. 2022; Carberry Mogan et al. 2021, 2022; Vorburger et al. 2015, 2019). The atmosphere of Callisto is not a pure exosphere everywhere: above the subsolar regions with their increased water sublimation rates, atmospheric densities are high enough that collisions must be taken into account for modelling (Carberry Mogan et al. 2021). The CO_2_ component is generally assumed to be spherically symmetric (e.g., Carlson 1999), while H_2_O is expected to exhibit a strong day-night asymmetry due to the effects of surface temperature on the H_2_O vapour pressure (Hartkorn et al. 2017). Hartkorn et al. (2017) compared O_2_ densities inferred from Galileo radio occultation observations at the terminator and HST/COS observations of the oxygen UV emissions on the dayside leading hemisphere and demonstrated that a similar day-night asymmetry in the O_2_ column density is likely also present, potentially resulting from the temperature-dependent sputtering yield of O_2_ molecules. The contribution of sputtering to the generation of Callisto’s atmosphere in general is complicated by the presence of a substantial ionosphere, which is expected to shield the surface from cold plasma ion sputtering while the hot plasma precipitation remains unaffected (e.g., Vorburger et al. 2019). The degree of shielding may vary considerably based on Callisto’s location relative to the centre of Jupiter’s current sheet (Galli et al. 2022; Liuzzo et al. 2019a, 2019b). The predicted JUICE trajectory used for these simulations (CReMA 5.0) includes Callisto flybys at a range of magnetic latitudes, sampling both the upstream (trailing hemisphere) and downstream (leading hemisphere) plasma environments, providing the opportunity to constrain the varying plasma-moon interaction (e.g., Galli et al. 2022). Predicted column densities for major atmospheric species at the JUICE closest approach altitude are shown in Fig. 12.

While it is up to the JUICE mission to clarify whether Callisto is a fully differentiated body, about half of the material on the surface is of mineral composition, typically assumed to be compositions of CI as well as L type chondrites. Therefore, because of sputtering by magnetospheric ions, many different species related to these minerals are expected in Callisto’s atmosphere (Vorburger et al. 2019).

### Connections Between Surface Composition and Atmosphere

**Fig. 13:**
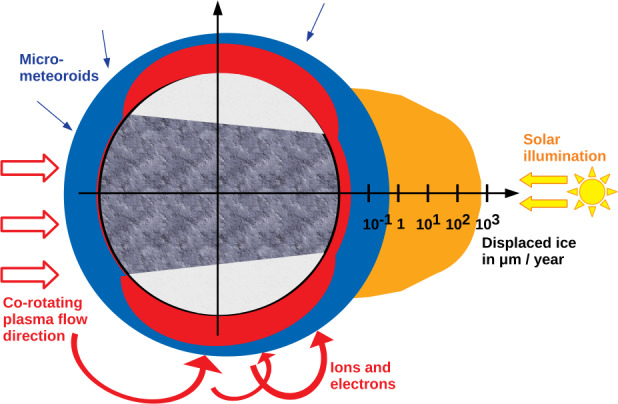
Sketch of water ice removal or turnover rates on Ganymede’s surface in units of μm yr^−1^. Orange shape: effect of thermal sublimation, blue: micrometeoroid impacts, red: irradiation-related rates (dominated by ion sputtering). Note the logarithmic scale of the rates (going from 0.1 to 1000 μm yr^−1^). The sketch is centred on the equatorial plane of the anti-Jovian side. Ganymede’s leading hemisphere with respect to Jupiter, the Jovian plasma is on the dayside to the right. The grey band and the white pole caps symbolise the surface areas of low and high albedo. Figure reproduced from Galli et al. (2024)

The surfaces of Jupiter’s icy moons act as both sources and sinks of atmospheric species. Each satellite experiences: 1) sputtering and radiolysis caused by charged particles, 2) sublimation of volatile surface species, 3) micrometeoroid impact vaporisation (MIV), and 4) photo-stimulated desorption. The latter process is usually assumed to be negligible at the Galilean moons (see e.g. Plainaki et al. 2010), but the first three processes are relevant to varying degrees across the surfaces of Europa, Ganymede, and Callisto as mechanisms for the release of materials into the atmosphere and/or chemical and physical alteration of the surface (Galli et al. 2024 and references therein). Figure 13 illustrates the relative importance of sputtering, sublimation, and MIV for the case of water ice on Ganymede’s surface. In the following subsections we will briefly characterise these surface interaction processes and summarise how their relative importance varies for each of the icy moons.

#### Sputtering of Surface Materials

Precipitation of ions and electrons onto the moon surfaces, or charged particle surface irradiation, is a major contributor to the generation of the tenuous atmosphere of Europa (e.g., Ip 1996; Shematovich and Johnson 2001; Shematovich et al. 2005; Plainaki et al. 2018), Ganymede (Marconi 2007; Shematovich 2016), and Callisto (Vorburger et al. 2019). Irradiation processes fall into two broad categories: sputtering and radiolysis. Sputtering describes the ejection of surface species by an impacting energetic particle (Brown et al. 1978). Radiolysis describes the chemical alteration of surface species induced by the deposited energy of the particles. The particles sputtered from the surface may be the original molecules (e.g., H_2_O), molecule or atomic fragments (e.g., OH, O), or radiolysis products such as O_2_ and O_3_ (Teolis et al. 2017a; Galli et al. 2018). The detection of O_2_ (Spencer et al. 1995; Migliorini et al. 2022) and O_3_ (Noll et al. 1996) in the surface of Ganymede, for example, indicates radiolysis of water ice. Dark material on Europa’s trailing hemisphere is thought to comprise radiolytically processed sulphur compounds (Carlson et al. 1999a, 1999b), and a possible UV signature of H_2_O_2_ on Callisto may similarly be a result of radiolysis (Hendrix and Johnson 2008). As well as being directly sputtered from the surfaces, radiolytic products produced in the icy surfaces of the Galilean moons can diffuse through the ice and escape to the atmospheres.

The sputtering yield (i.e., the number of ejected neutrals per charged particle incident on the surface) depends on the energy and mass of the impacting electron or ion, the angle of impact, and the surface temperature (e.g., Famá et al. 2008). Hence, variations in the sputtering yield between different surface regions on each icy satellite are expected, as well as differences between moons. Although Europa has the coldest surface, it has the highest sputtering yield since it orbits in a region where the Jovian magnetospheric plasma density and magnetic field strength are increased relative to the environment experienced by Ganymede and Callisto. At Ganymede, the satellite’s mini magnetosphere restricts charged particle access to the trailing hemisphere mid-latitudes but charged particles can impact the surface on the leading hemisphere in the open field line regions near the poles (e.g., Poppe et al. 2018). The ∼10° offset between Jupiter’s magnetic dipole axis and its rotation axis means that all of the Galilean moons experience periodically varying plasma environments as Jupiter rotates. JUICE PEP will detect sputtered material in the exospheres of all three moons, determining how the sputtering yield varies temporally and as a function of surface position.

#### Sublimation of Surface Volatiles

Solar irradiation leads to sublimation of H_2_O and thermal desorption of non-condensable volatiles trapped in the regolith of the icy Galilean satellites. This process is straightforward to quantify in terms of theory and laboratory experiments. On Ganymede, thermal sublimation is the dominant source of atmospheric H_2_O (Marconi 2007; Plainaki et al. 2015; Shematovich 2016). Sublimation rates are highest at the equatorial regions because they encounter the most solar illumination, and their surface is darker compared to the polar regions. Dayside surface temperatures on Ganymede range from ∼100 K near the poles to ∼150 K at the subsolar point (Orton et al. 1996; Ligier et al. 2019; Galli et al. 2024). Sublimation of H_2_O is strongly temperature dependent: Roth et al. (2021) found that the temperature difference between Ganymede’s dayside trailing (∼148 K at the subsolar point) and leading (∼142 K) hemispheres was sufficient to explain a factor of five H_2_O density difference observed in the atmospheres above the centres of the two hemispheres. Since Callisto is darker and presumably warmer than Ganymede, it is likely that sublimation is also the dominant source of atmospheric H_2_O there. At Europa, the relative importance of sputtering and sublimation to the measured H_2_O abundance is less clear. While Roth (2021) detected an H_2_O atmosphere on Europa’s warmer trailing hemisphere, the maximum surface temperature of 130 K is not warm enough to account for the inferred H_2_O density. However, the sputtering yields of H_2_O at 130 K are also too low to sustain the observed atmosphere. Roth (2021) suggested that secondary sublimation of sputtered H_2_O molecules that fall back to the surface (e.g., Teolis et al. 2017a, 2017b) may explain the discrepancy. JUICE will study the contribution of sublimation to the satellite atmospheres by performing in situ measurements and remote observations of the daysides and nightsides during flybys of all three satellites.

#### Dust Exospheres of Europa, Ganymede and Callisto

**Fig. 14:**
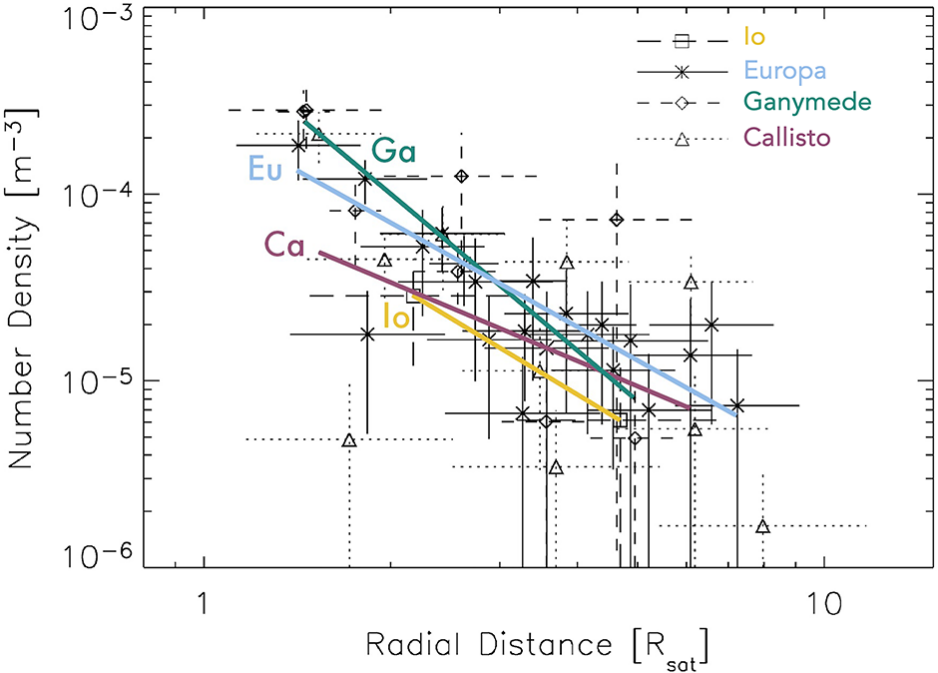
Number density of dust as a function of radial distance from the centre of Ganymede, Europa, Callisto, and Io, measured by the Galileo Dust Detector System. Adapted from Krüger et al. (2003)

Ganymede was the first object in the Solar System where an impact generated ballistic dust exosphere was detected. In 1996, during a series of close flybys of the Galileo spacecraft, the onboard dust detector recorded a series of impact events (Krüger et al. 1999). Later measurements found that all Galilean moons are enshrouded by a dust cloud (Krüger et al. 2003) (Fig. 14). The detected particle size distribution and their dependence on altitude is consistent with impact ejecta models (e.g., Krivov et al. 2003) where the most efficient impactor population consists of micrometeorites. These are relatively large interplanetary dust particles with typical sizes of approximately 10–100 μm (Divine 1993) and typical speeds of a few 10 km/s, which eject orders of magnitude more mass than the impacting particle in the form of mostly smaller dust ejecta at much lower speeds (on the order of 100 m/s). Due to the high escape speed of the Galilean moons nearly all ejected particles eventually fall back to the surface on ballistic trajectories, enshrouding them in a permanent dust exosphere and contributing to the regolith which is assumed to cover the moon’s surface.

Based on Galileo measurements, model predictions can be made to estimate detection rates for in situ instrumentation onboard a spacecraft either during a flyby or in orbit (Postberg et al. 2011b). For example, assuming apex pointing, JUICE PEP-NIM would encounter on the order of 10–30 dust samples lifted from Ganymede’s surface per day in a 500 km orbit, assuming a detection threshold radius of ∼200 nm. In a 200 km orbit this number would go up by about a factor of ∼5. Per flyby with closest approach <500 km, 0–1 dust particles are expected to enter NIM. Upon impact in the NIM antechamber or entrance slit, the volatiles in the dust grain will evaporate and will thus be resolved in a mass spectrum like other neutral gas signals. However, no information on the impact speed can be derived with NIM. The JUICE RPWI instrument can also detect micrometeorite impacts and estimate dust charging. Together, these instruments will provide an improved understanding of interactions between the surfaces of the moons and their surrounding dusty exospheres.

### Atmosphere Characterization with JUICE Instruments

The diverse instruments carried by JUICE will perform complementary in situ and remote sensing studies of the composition, structure, and variability of the atmospheres of Jupiter’s icy moons. In situ detection and characterization of the moon atmospheric species will be achieved by the PEP-NIM mass spectrometer, which will measure ice and mineral species sputtered from the surfaces, as well as sublimating volatiles and any material outgassing from the subsurface or deeper interior (GC.4e, EB.3d, CB.3c) The volatile composition of sporadic dust grains entering NIM during the neutral gas measurements will also be evaluated. Other PEP instruments will provide remote sensing of the neutral atmospheres via Energetic Neutral Atom (ENA) imaging (PEP-JNA at low energy, PEP-JENI at high energies). Charge exchange between an energetic ion and a neutral atom results in a thermal ion and an ENA, the latter of which travels away on a straight trajectory with the energy of the parent ion, allowing remote sensing of the neutral environment around the icy moons by the PEP sensors.

**Fig. 15:**
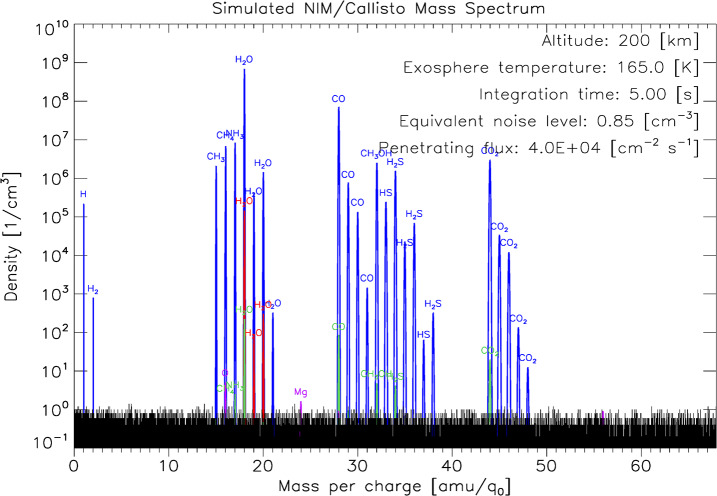
Predicted mass spectrum to be recorded by PEP/NIM at the closest distance of JUICE to Callisto (200 km) for an integration time of 5 s. Shown are the spectra of the sublimated species (blue), the icy sputtered species (green), the mineral sputtered species (magenta), and photon-desorbed H_2_O (red). Also shown is NIM’s expected detection threshold of 1 cm^−3^ (instrument background) in black. Figure taken from Galli et al. (2022)

For Callisto, the PEP measurement opportunities and goals were described by Galli et al. (2022). The baseline predicted trajectory (CReMA 5.0) foresees 21 Callisto flybys, with closest approaches both on the dayside and the nightside. The 13 flybys with a closest approach below 1000 km will be crucial to detect heavy neutrals and ions in-situ (see Fig. 15 for a simulated spectrum at an altitude of 200 km). Nevertheless, neutral measurements will be started at distances >40 R_C_ away from Callisto to better constrain the putative neutral torus and the extended hydrogen corona. At the same time, remote ENA measurements will provide global images of the interaction of Callisto’s surface and atmosphere with the plasma environment and thus give important context. Fortunately, the background rates due to radiation levels in the Jovian magnetosphere at Callisto will be much lower than near Europa or Ganymede (Galli et al. 2022).

The JUICE remote sensing instruments will similarly perform comprehensive studies of the icy satellite atmospheres. The JUICE UVS bandpass of 50–204 nm encompasses emission lines of key atmospheric species including the neutral O (130.4 nm, 135.6 nm) and H (121.6 nm) emissions previously observed at Jupiter’s icy moons by HST. UVS will perform disk scans during the inbound and outbound phases of satellite flybys to map these emissions and study their variability with local time, longitude and latitude, and local magnetic environment (GC.4a, EC.2a, CB.3b). Long stares at the sunlit limbs of the satellites will be used to search for faint emissions of potential minor atmospheric species including N, C, CO (fourth positive band), H_2_ (Lyman and Werner bands), S, Cl, Ca, and Mg. UVS will also perform stellar occultation measurements, during which stellar spectra are collected as the line-of-sight from UVS to the star moves through a satellite atmosphere. The presence of any UV-absorbing species such as H_2_O, O_2_, CO_2_, H_2_, CH_4_, and C_2_H_2_ will modify the observed stellar signal in a predictable way, allowing the concentrations and distributions of these materials to be constrained. Similar but less frequent solar occultation measurements will facilitate searches for species absorbing at wavelengths <100 nm, where stellar flux is low due to extinction by the interstellar medium. Absorption of Jupiter’s dayglow by the satellite atmospheres as the moons transit across Jupiter’s disk will also be performed. These transit observations will be used to search for regions of increased Lyman-$\alpha $ absorption on the satellite limb indicative of localised H_2_O from potential plume activity.

MAJIS will use limb scans to map non-LTE emissions of atmospheric species including H_2_O (2.4–3 μm), CO_2_ (4.26 μm) and O_2_ (1.27 μm), allowing retrieval of density profiles (GC.4b, EC.2b, CB.3a). The instrument is also sensitive to auroral emissions in the visible wavelength region – H$\alpha $ at 656.3 nm and OI at 557.7 nm, 630.0 nm, 636.4 nm (GC.3d). The relative intensities of these emissions and the UV aurora measured by UVS will provide tighter constraints on atmospheric composition and density, as well as the density and energy of the electrons exciting the emissions. Stellar occultation measurements will be used to determine the distribution of atmospheric species that absorb light in the visible to near-infrared spectral region. Where possible, stellar occultations will be performed simultaneously with UVS, although only a small number of stars provide adequate flux at both UV and IR wavelengths (usually unresolved multiple star systems rather than single stars). MAJIS will also search for active plumes by observing forward scattered light from possible plume grains along the satellite limbs at high phase angle (see Sect. 4.4); similar observations by UVS and JANUS may also contribute to this goal. JANUS will contribute to atmospheric studies via global imaging of the sodium exosphere at Europa (EC.1d) and monitoring of optical auroral emissions at all three icy satellites.

SWI will complement the other remote sensing studies with observations of rotational transitions of water and its isotopes, and several other molecular species including CO, HCN, O_2_, SO_2_, and potentially also their main ionic counterparts, in the frequency ranges 530–625 and 1080–1275 GHz at high spectral resolution ($\sim 10^{7}$). SWI will study the atmospheric structure (density, temperature and winds), isotopic composition, and distribution of the water atmospheres of Ganymede, Callisto, and Europa from the surface to altitudes of a few hundred km (GC.4c, EB.3g, EC.2e, CB.3f). From measurements of the exact position and shape of the molecular lines, parameters such as the regions of transition between collision and non-collision dominated parts of the satellite atmospheres, as well as wind and temperature profiles, may be derived. SWI can also measure the ortho-to-para ratio of gaseous water, which serves as a proxy for the conditions under which the water formed. While SWI will map the atmosphere with high spatial resolution maps, limb stares and limb scans during the various flybys and GCO, its scanning mechanism also allows routine observations of the Jovian moons at almost any time from the orbit around Jupiter. On average, SWI will dedicate one hour per day to observations of the different moons. It will be sensitive enough to sample the moon’s atmospheric conditions as a function of solar illumination on the leading and trailing sides. The spatial resolution of this long baseline of observations will vary with time and range from point source observations to moderate resolution maps of surface and atmosphere emissions. In particular, 3D radiative transfer models demonstrate (Wirström et al. 2020) that sets of five-point cross maps of Ganymede in the 557 GHz water transition for a range of phase angles should allow us to determine the dominating source for the water atmosphere, thermal sublimation or sputtering. The long-term monitoring from the Jovian orbit will thus provide a global perspective on the neutral atmospheres around the moons and help to set up synergies with and for other instruments in preparation of the various flybys and GCO, so as to focus on potential regions of interests discovered during these campaigns.

In addition to broad atmospheric studies by the in situ and remote sensing instruments discussed above, other JUICE instruments will contribute to more specific atmospheric science goals. RPWI will measure the mass and size distribution of charged dust particles within the satellite exospheres (GE.2c, EC.1c, CB.1f, CB.2f), studying how they are accelerated towards the surface where they contribute to sputtering of material into the exospheres. JMAG may detect magnetic field perturbations due to atmospheric inhomogeneities: for example, models by Blöcker et al. (2016) show that Alfvén winglets are expected to develop within Europa’s Alfvén wing if plume activity occurs near the north or south pole of Europa.

**Fig. 16:**
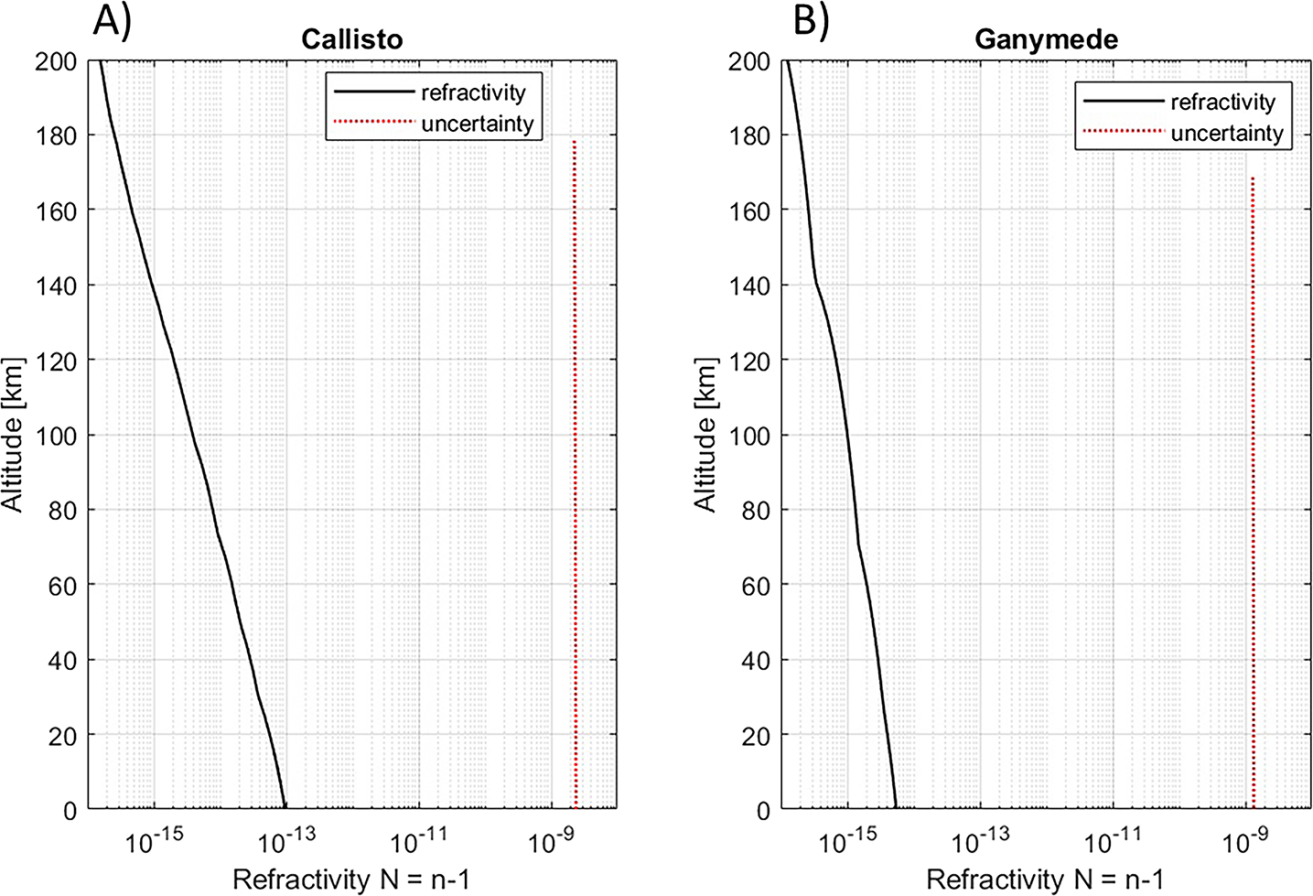
Predicted refractivity height profile of Callisto (panel A) and Ganymede (panel B) (black line), compared to the expected measurement’s uncertainty (red dotted line) of the 3GM radio science experiment (figure courtesy of A. Caruso)

3GM will probably not be able to contribute to measuring the density or temperature profiles of the neutral atmospheres at Callisto, Ganymede, or Europa. This is illustrated by Fig. 16 showing the refractivity profile calculated for radio occultations near Callisto (panel A) and Ganymede (panel B) (black line, evaluated for the number density and the chemical species expected according to Liang et al. 2005 and Marconi (2007) compared with the expected uncertainty (red dotted line) for 3GM measurements. The latter was computed using the algorithm developed by Bourgoin et al. (2022), assuming a radio link stability of $3 \times 10^{13}$ at 1 s integration time. The uncertainty is several orders of magnitude larger than the predicted value for the refractivity. For Europa, assuming CReMA 5.0, suitable radio occultation opportunities were not identified. However, being the expected refractivity (computed from the neutral number density reported in Johnson et al. (2009)) similar to the one of the other two moons, similar results and conclusions are expected.

### Potential Plume Detection and Characterization (Both Direct Detection and Inferred from Surface Features at Europa and Ganymede)

Several pieces of evidence for water plumes at Europa have been reported over the last decade, but conclusive proof of active plumes has not yet been achieved. The question thus remains an active research topic. The first tentative detection came in the form of HST observations of auroral emissions consistent with electron impact dissociation of localised H_2_O vapour (Roth et al. 2014a). Additional HST observations of shadowy features on the limb of Europa as it transited Jupiter were interpreted as absorption of Jupiter dayglow by water plumes (Sparks et al. 2016, 2017, 2019), though this interpretation is disputed (Giono et al. 2020). Furthermore, magnetic field perturbations detected during the E12 and E26 flyby by the Galileo mission have been shown to be consistent with the presence of water plumes (Blöcker et al. 2016; Jia et al. 2018; Arnold et al. 2020). Dropouts of energetic protons during E26 were reported to be caused by the plume from Arnold et al. (2020) by Huybrighs et al. 2020; however, an artefact in the data prevents a definitive conclusion (Jia et al. 2021; Huybrighs et al. 2021). Infrared Keck telescope detections of H_2_O have also been interpreted as a potential plume eruption (Paganini et al. 2020). If plumes are present, their activity appears to be intermittent and unrelated to Europa’s orbital position (Roth et al. 2014b; Paganini et al. 2020). Recently, JWST searched for plumes at Europa but yielded no detection of water, carbon monoxide, methanol, ethane, or methane fluorescence emissions (Villanueva et al. 2023). During the Juno close flyby of Europa occurred in September 2022, data collected by the JunoCam framing camera (Hansen et al. 2024) and by the plasma wave instrument (Kurth et al. 2023) did not find evidence of active plume eruptions.

Any ongoing or very recent plume activity at Europa would be detectable through atmospheric observations by JUICE and may also result in surface features observable by the remote sensing instruments. Freshly deposited water ice will appear brighter than older terrain that has been processed by the impinging Jovian plasma or experienced thermal segregation, where ice sublimating from warmer, darker regions is preferentially deposited in cooler, brighter regions (Spencer 1987). The size distribution of ice grains on the surface could also be an indicator for recent plume activity, but it is also an important and ill-constrained parameter for modelling plumes (Quick and Hedman 2020). Fresh H_2_O deposits will be detectable by UVS and MAJIS via characteristic UV and IR spectral features, as well as appearing as bright regions in JANUS visible images. Belgacem et al. (2020) identified two potential regions of fresh water ice in Voyager and New Horizons images of Europa’s leading hemisphere that may be related to plume activity. Although there are currently no constraints on any non-water constituents of the plumes, by analogy with the Enceladus plumes (Dougherty et al. 2006) it is possible that they would contain darker materials such as salts and organics, which may be detected on the surface. This low-albedo plume material may be responsible for dark deposits observed around lenticulae and along lineae on Europa (Fagents et al. 2000; Quick et al. 2013), the distribution and composition of which will be studied by MAJIS, JANUS and UVS.

Similarly, JUICE will search for evidence of past or present plume activity on Ganymede’s surface, focusing on paterae – small, scalloped-edged depressions potentially linked to cryovolcanism (Lucchitta 1980; Schenk and Moore 1995; Kay and Head 1999; Schenk et al. 2001; Spaun et al. 2001), which are currently the only known surface features indicative of activity. JUICE will study the origin and formation of these features using high-resolution imaging and spectral imaging (JANUS, UVS, MAJIS, SWI) together with laser altimetry (GALA) and subsurface radar (RIME) measurements (Stephan et al. 2020). We note that the recent flyby of Ganymede by Juno (Hansen et al. 2022) identified 10 previously unknown paterae in the region covering Phrygia Sulcus, Sicyon Sulcus, and eastern Perrine Regio (∼10°E–50°W longitude and 10°S–50°N latitudes), bringing the total to 47 known paterae across the full surface (Ravine et al. 2022). This suggests that either the features are atypically concentrated in specific surface regions, potentially providing clues as to their history, or more remain to be discovered by JUICE. While Ganymede’s paterae appear ancient, improved constraints on their ages and formation mechanisms will help us to better understand whether ongoing cryovolcanism is likely. Any ancient plume material present may be identified by correlating potentially endogenic materials such as salts and ammoniated species with the geology, while the age of such material may be constrained by comparing the observed reflectance spectra to pristine and irradiated sample spectra. Salts, for example, develop characteristic colour centres when exposed to charged particle radiation, leading to the identification of NaCl on Europa (Trumbo et al. 2019a). Any ongoing plume activity will be characterised using the same methods described for the Europan plumes in the remainder of this Section. A recent search for active water plumes via attenuation of Jupiter’s Lyman-$\alpha $ airglow as Ganymede transited Jupiter placed an upper limit for localised (plume) H_2_O column densities of $(2\text{--}3) \times 10^{16}\text{ cm}^{-2}$ (Roth et al. 2023), which is similar to the plume column density derived by Roth et al. (2014a) at Europa.

UVS will search active outgassing at plumes using different methods. Emissions from excited atomic oxygen and hydrogen, which might be indicative of electron-impact dissociation of plume molecules, are used as diagnostic in global scans. This method is analogous to the plume aurora observations by HST (Roth et al. 2014a). In addition, UVS will probe continuum absorption by H_2_O (or other gases) during star occultations and Jupiter transits, similar to the Enceladus plume gas observations by Cassini UVIS (e.g., Hansen et al. 2019, 2020).

Ongoing plume activity will also be targeted by SWI with daily monitoring of the moons. SWI will have the sensitivity to detect H_2_O plumes from large distances, and even more so during flybys and GCO phases which may allow detection of other species from confirmed active sources. The clues that a given measurement is dominated by molecules from a transient source come from a) horizontal and/or vertical gradients on the measured densities and b) directly through the resolved line shapes at 600 or 1200 GHz, i.e., their amplitudes and Doppler shifts, as they will be statistically different from the baseline established during quiescent atmospheric conditions. Since SWI targets low excitation rotation lines it may also be able to detect transient sources more directly from observations at the terminator in nadir or limb geometry, and even over the cold night-time surface over the un-illuminated hemisphere.

**Fig. 17:**
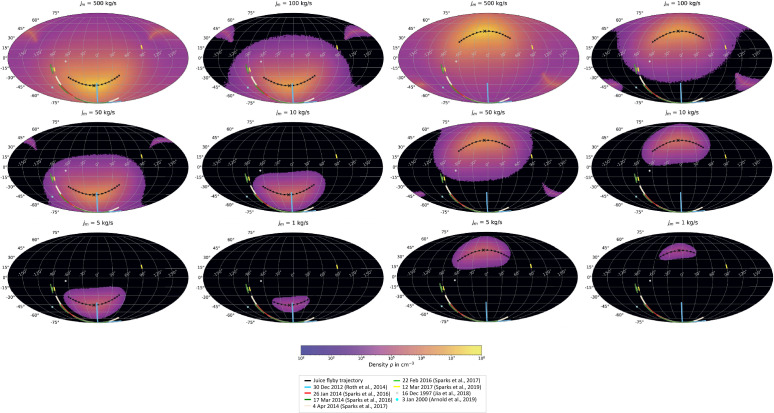
Colour maps expressing the H_2_O density predicted along the JUICE trajectory as a function of plume location on the surface. Left two columns first flyby, second two columns second flyby. Blacked out areas correspond to regions where the plume density along the trajectory does not exceed the atmospheric H_2_O density using an atmospheric profile from Shematovich et al. 2005. Black line indicates the spacecraft trajectory. Presumptive plume sources indicated by coloured lines and dots. [Reproduced from Winterhalder and Huybrighs (2022)]

**Fig. 18:**
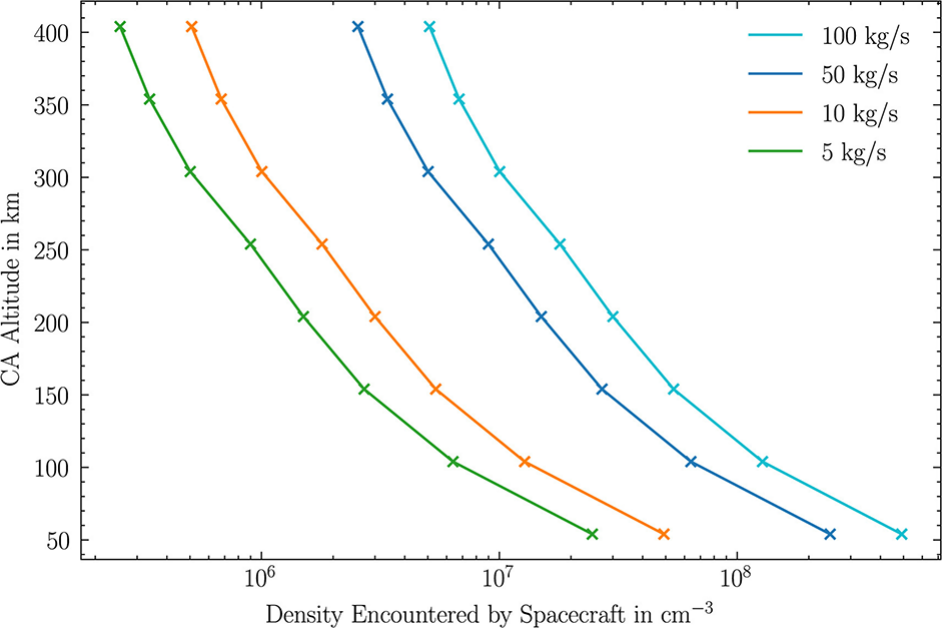
Density encountered by the spacecraft if an active plume source was present right beneath the closest approach point. As expected, the signal increases as the spacecraft’s closest approach point moves closer to the moon’s surface. [Reproduced from Winterhalder and Huybrighs (2022)]

**Fig. 19:**
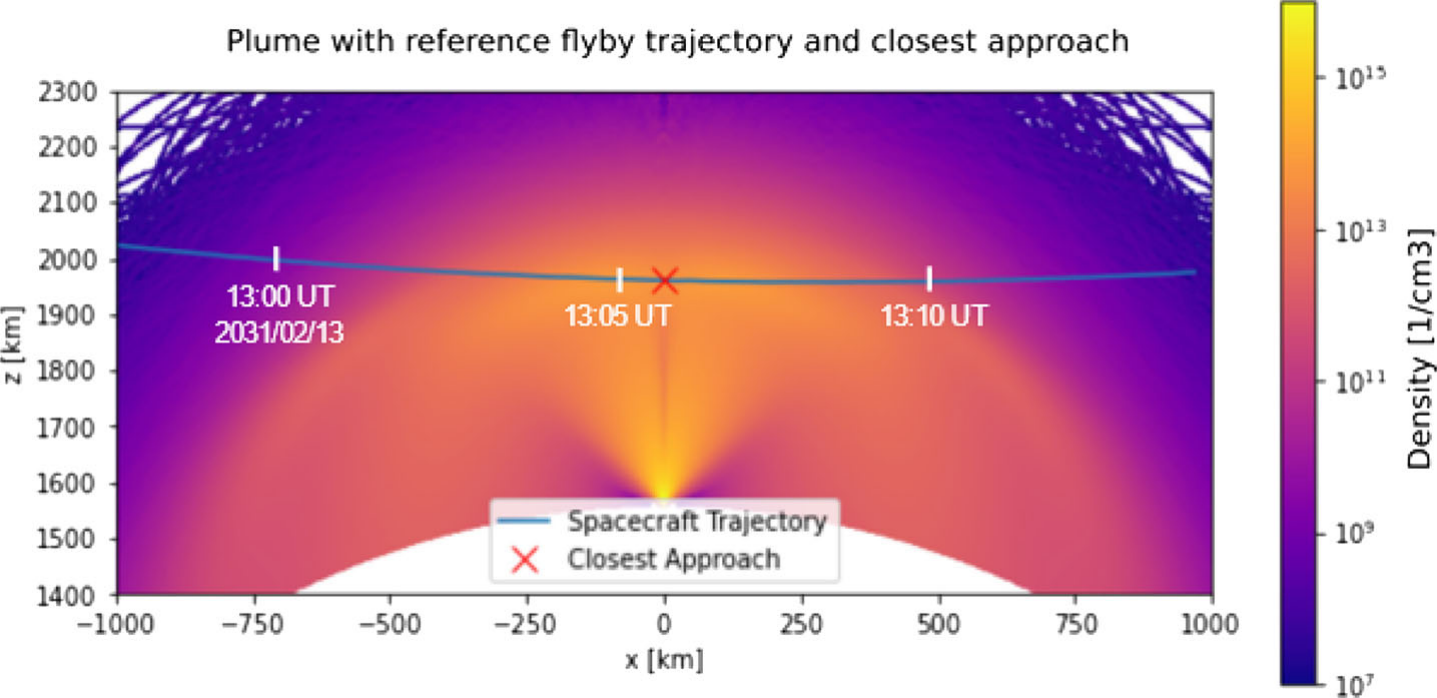
JUICE trajectory through a model 100 kg/s collisional plume, with closest approach at an altitude of 400 km. The time that JUICE encounters the plume (∼10 mins) well exceeds the PEP-NIM integration time (5 s), therefore the plume structure (e.g. the canopy shock) can be discerned, allowing to empirically constrain plume models and study internal plume physics. [Reproduced from Dayton-Oxland et al. 2023]

The possibility that ongoing plume activity could transport material from Europa’s subsurface, or from water reservoirs contained in the ice layer, creates an unprecedented opportunity to sample Europa’s subsurface environment and investigate its habitability. PEP-NIM and PEP-JDC are suitable for directly detecting H_2_O and H_2_O^+^ from Europa’s water plumes and separating it from atmospheric H_2_O and H_2_O^+^ based on the observed density distribution (Huybrighs et al. 2017). If the locations of plume activity identified in previous tentative detections are accurate guides to the plume distribution, the coverage of the southern hemisphere JUICE flyby is most suitable to detect the presumptive plume sources, since most are located on Europa’s southern hemisphere (Fig. 17). Due to its location, the potential plume reported by (Roth et al. 2014a) is the most likely to be detected even when the plume mass flux is as low as 1 kg/s (Fig. 17 and Winterhalder and Huybrighs (2022)). If the plume flux is larger than 1 kg/s, the formation of a canopy shock due to collisions between plume particles may limit the height of the plume and reduce the H_2_O density along the JUICE trajectory (Dayton-Oxland et al. 2023). This could result in a reduction of the region over Europa’s surface within which plumes would be separable from the H_2_O atmosphere by JUICE by up to a half. Lowering the flyby altitude would result in an increase in the signal strength of detected plume particles by an order of magnitude in the non-collisional and low mass flux case (Fig. 18) and would increase the likelihood of flying through or below the canopy shock in the collisional and high mass flux case. Flying below the shock decreases the risk of losing detection coverage, decreased density and duration (Dayton-Oxland et al. 2023). A flyby with a closest approach of 400 km that passes directly over a plume would encounter the plume for several minutes (see Fig. 19), which is much longer than the PEP-NIM integration time (5 s), giving good temporal (and therefore spatial) resolution of samples of the plume. Measurements of the structure of the plume would provide an empirical constraint on plume models, by which we can investigate the underlying physics and typical characteristics of Europa’s plumes (Dayton-Oxland et al. 2023).

Besides direct in situ detections of plume particles, other in situ instruments can also be used to infer the presence of plumes. JMAG could be used to infer the presence of plumes from magnetic field perturbations, while PEP-JENI could detect plumes through dropouts of energetic ions and ENA emissions (e.g., Huybrighs et al. 2020, 2021). RWPI could detect enhancements in the ionospheric density related to potential plumes. Furthermore, remote sensing observations can be used to detect plumes, both during flybys and in more distant limb stares and disk scan observations. UVS will search for localised hydrogen and oxygen auroral emission associated with plumes that are being bombarded by magnetospheric plasma, as well as localised absorption of stellar or solar light or Jupiter airglow by H_2_O during occultation and transit events. MAJIS is similarly capable of remote mapping of H_2_O in Europa’s exosphere, and SWI will also investigate the distribution and variability of exospheric H_2_O, searching for localised, transient enhancements of water vapour. Evidence for a recently terminated plume eruption could be detected via remote sensing of the surface (heavy molecules close to the eruption site resulting in localised surface enrichments) or in the global exospheric composition as a ratio to O_2_ (Teolis et al. 2017b).

Finally, MAJIS, JANUS and UVS can potentially search for active plumes by observing solar light scattered by plume grains along the satellite limb. This technique is most effective in high phase angle configurations, similar to those yielding the first images of Enceladus’ plumes (Porco et al. 2006) and their subsequent imaging by both the Cassini/ISS camera (Porco et al. 2014) and the Cassini/VIMS spectrometer (Hedman et al. 2009). Here, we provide an assessment of the feasibility of this plume detection technique at Europa and Ganymede, using MAJIS as a case study. We note that this technique will likely be difficult for UVS due to the lower solar flux at UV wavelengths and low particle scattering efficiencies, but the method has previously been used by the Lyman Alpha Mapping Project (LAMP) – a UVS sister instrument on NASA’s Lunar Reconnaissance Orbiter – to place limits on the lunar dust population (Feldman et al. 2014).

**Fig. 20:**
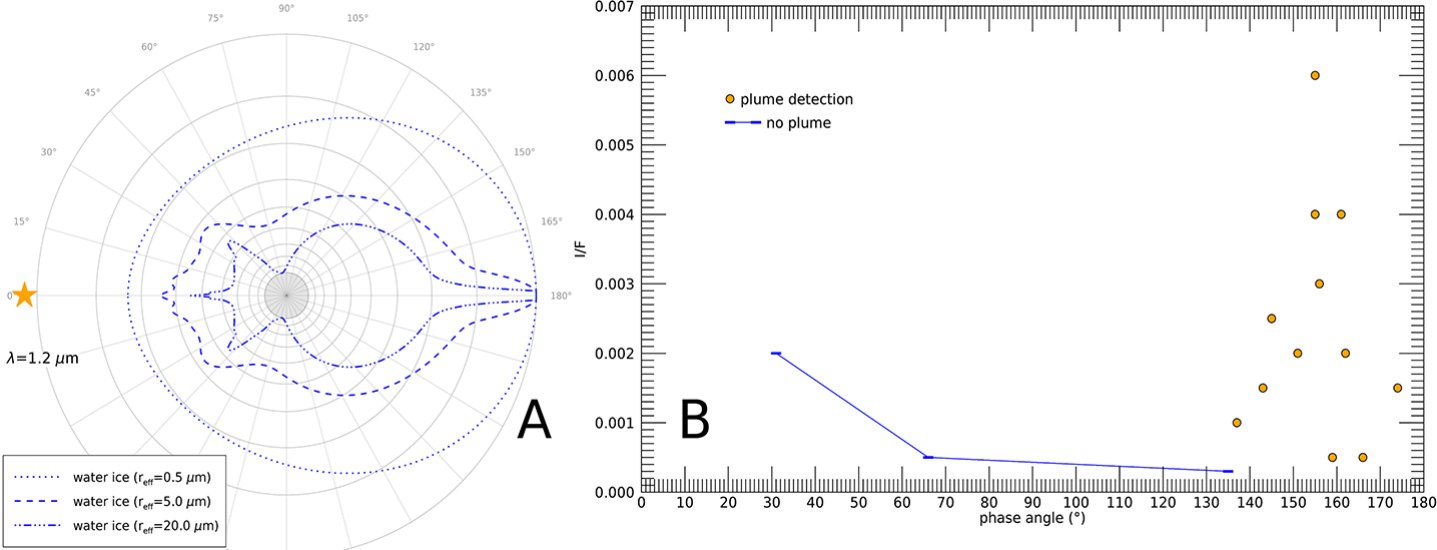
**Panel A:** Variation of water ice scattering phase function with respect to grain size, evaluated at 1.2 μm wavelength through the Mie theory for lognormal distributions with effective radius of 0.5 μm (dotted line), 5 μm (dashed line), and 20 μm (dot-dashed line). The angular values on the grid represent the phase angle, hence the light source is at the left (star symbol). All curves are normalised to their maximum value, which is at 180° phase angle. **Panel B:** Enceladus plume reflectance level (∼1.0–2.4 μm) resulting from a preliminary overview of a sample of Cassini/VIMS data as a function of solar phase angle (orange circles). Cases of non-detection are reported as upper limits in blue

The scattering efficiency of a plume is a function of the microphysics of its grains, the solar phase angle, and wavelength ($\lambda $), and all these quantities must be considered when evaluating the possibility of a direct detection by MAJIS. The enhancement of light scattering close to the forward direction is a well-known feature for grains of size larger than the wavelength. Figure 20A shows the single scattering phase functions of water ice grains at $\lambda = 1.2~\upmu \text{m}$ for three grain radii in the range 0.5–20 μm (evaluated through the Mie theory applied to lognormal distributions and refractive index from Mastrapa et al. 2008). For reference, the grain radius at Enceladus inferred from VIMS analysis is in the range 2–5 μm (Hedman et al. 2009). The fact that both the intensity and the width of the forward scattering lobe are quite dependent on the grain size parameter ($2 \frac{\pi r}{\lambda} $), may allow limb satellite imaging at different phases to help constrain the microphysics of eventual plumes but in any case, observations at smaller phase angles are much less likely to succeed. Information about the grain size distribution may also be gained by comparison with complementary observations by UVS and JANUS, due to the wavelength dependency.

To determine a suitable phase angle threshold for MAJIS, we consider the Cassini/VIMS dataset about the Enceladus plume. A preliminary analysis is shown in Fig. 20B, where a collection of VIMS plume reflectance levels at $\lambda = 2.2$–2.4 μm is plotted as a function of phase angle, along with the cases where no plume signal is detected. Although the selected VIMS data refer to a rather wide range of spatial resolutions (10–100 km/px), a detection threshold on phase angle between 135°–140° can be envisaged.

An additional parameter affecting the chance of plume detection by MAJIS is the achievable spatial resolution, which directly impacts the filling factor of the MAJIS pixel (angular resolution = 150 μrad). The tentative detections at Europa suggest a vertical extension of 200 km (Roth et al. 2014a). In the absence of constraints at the more massive Ganymede, our analysis also assumes this scale length as an upper limit for any plumes that may exist there.

**Fig. 21:**
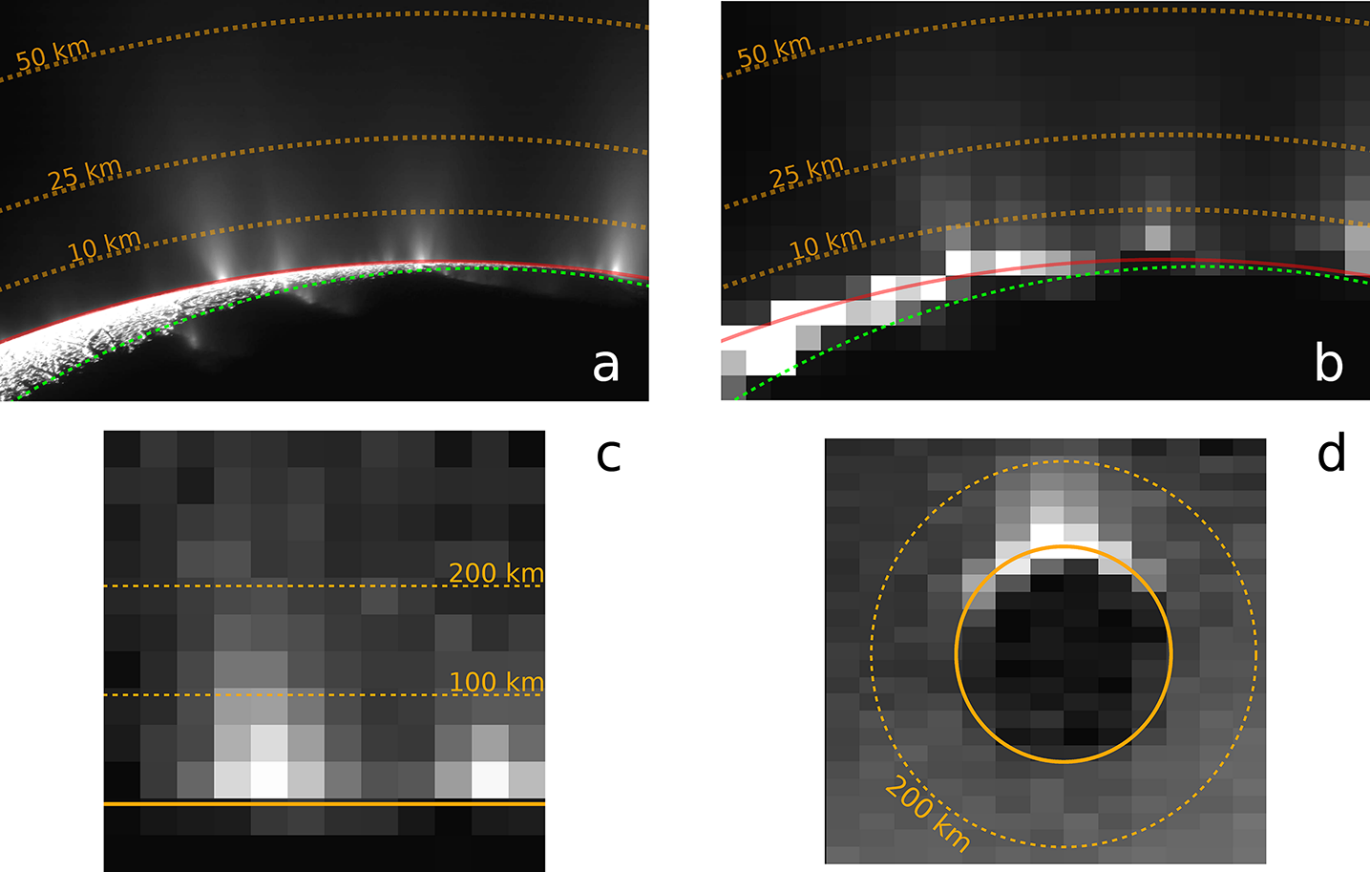
Effect of spatial resolution on the appearance of plumes on Enceladus’ South Pole. **Panel a**: Cassini/ISS image taken on 21 Nov 2009 at 145° solar phase angle, with a pixel scale of about 0.8 km/px (published in Porco et al. 2014). **Panel b**: The same image of Panel A degraded in resolution to simulate a view at 5 km/px. **Panel c**: Enceladus plumes imaged by Cassini/VIMS at near-infrared wavelengths (about 1–2 μm), nearly simultaneously to the image in Panel A, but with a resolution of about 33 km/pixel. **Panel d**: Another near-infrared VIMS image of Enceladus plumes at a resolution of about 40 km/pixel along the vertical direction. In all panels, dashed orange lines indicate different tangent altitudes above the satellite limb (solid line)

However, the typically narrow shape of plumes, and their possible apparent shortening due to satellite surface curvature, can make the scattered light signal dilute into background sky at lower altitudes. One example of the effect of spatial resolution on images can be seen in Fig. 21, where Cassini images in the visible (ISS) and in the near infrared (VIMS) at different resolution levels are shown. While investigating plume structures requires resolution of at least 5–10 km/px, VIMS detected plume signals at a resolution as low as 30–40 km/px. However, the extrapolation of these values to the Galilean’s case is not straightforward, since the activity level of Enceladus plumes is quite high and the gravity is much lower, resulting in a very diffuse feature like the E-ring which is not observed in the Jovian system.

A first analysis of a recent predicted JUICE trajectory (CReMA 5.0) shows that the best conditions for plume detection in terms of both phase (>135°) and spatial resolution (<30 km/px) are met only in the two Europa flybys in July 2032 and in six opportunities for Ganymede (see Table 2). During the Jovian tour mission phase, additional high phase angle opportunities are encountered for both targets, but with spatial resolutions worse than 80 km/px for Europa and 60 km/px for Ganymede. These conditions correspond to full disk images of the satellites (diameter smaller than 40 and 90 pixels respectively for Europa and Ganymede), that may only allow detection of rather dense plumes in single pixels.

**Table 2:** Opportunities for high phase angle views of Europa and Ganymede during the Jupiter tour mission phase, based on a recently predicted JUICE trajectory (CReMA 5.0)

UTC range	Phase (°)	MAJIS resolution (km/px)	Target
2032-JUL-02 16:53 – 2032-JUL-02 17:23	138	1.6	Europa
2032-JUL-16 22:46 – 2032-JUL-16 23:16	140	1.5	Europa
2032-JUL-18 03:08 – 2032-JUL-18 03:18	135	93	Europa
2034-JUN-29 10:32 – 2034-JUN-29 15:06	140	86	Europa
2034-AUG-14 13:19 – 2034-AUG-14 17:50	149	90	Europa
2034-OCT-28 05:10 – 2034-OCT-28 09:38	143	83	Europa
2034-DEC-06 07:13 – 2034-DEC-06 11:33	143	89	Europa
2032-FEB-13 23:17 – 2032-FEB-13 23:37	147	1.3	Ganymede
2032-APR-11 04:25 – 2032-APR-11 04:45	150	1.2	Ganymede
2032-MAY-09 19:02 – 2032-MAY-09 19:24	143	2.5	Ganymede
2032-JUN-02 21:33 – 2032-JUN-02 22:00	164	1.1	Ganymede
2033-NOV-27 05:53 – 2033-NOV-27 06:28	153	0.6	Ganymede
2034-JAN-17 20:34 – 2034-JAN-18 05:17	140	78	Ganymede
2034-JUN-06 05:04 – 2034-JUN-06 06:16	156	1.5	Ganymede
2034-SEP-05 17:30 – 2034-SEP-05 17:53	135	76	Ganymede
2034-SEP-27 03:22 – 2034-SEP-27 03:45	135	78	Ganymede
2034-NOV-16 12:12 – 2034-NOV-16 12:42	135	56	Ganymede
2034-DEC-14 17:21 – 2034-DEC-14 17:46	135	96	Ganymede

**Fig. 22:**
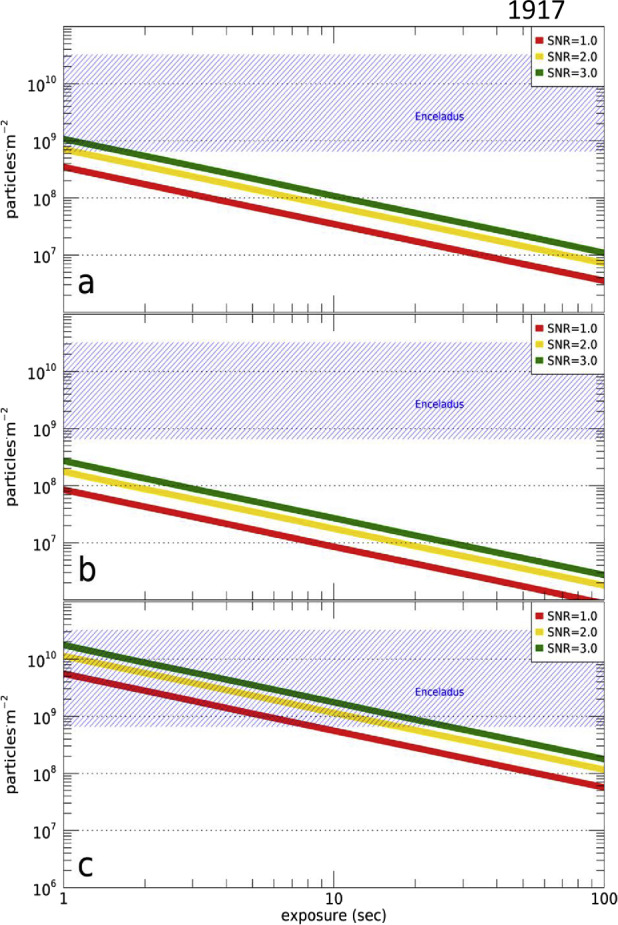
Detection limits of a water ice plume on Ganymede in three different JUICE mission phases: distant flyby (panel a), close flyby (panel b), high orbit (panel c). Red, orange, and green lines indicate the plume column density detectable with SNR values of 1, 2, or 3, respectively (as average value in the 0.8–1.0 μm range), compared to the Enceladus plume density range [Reproduced from Plainaki et al. 2020b]

Detection limits for plume detectability with MAJIS at Ganymede have been quantitatively investigated in Plainaki et al. (2020a, 2020b) for three mission phases (a distant flyby, a close flyby, and a high-altitude orbit like GCO5000), as a function of MAJIS exposure times and plume column densities. For the calculation, spherical homogeneous particles with a pure water composition and 3 μm effective radius are considered. Figure 22 shows the detectability of the column density with a signal-to-noise of 1, 2 or 3. The models show that the detection of possible plumes would be more favourable in case of a close flyby (Fig. 22b), which ensures the best conditions of resolution and phase angle suitable for low density plumes detection (1–2 orders of magnitude less dense than the Enceladus plumes), with low exposure times. In the other two cases analysed in Plainaki et al. (2020a, 2020b) plume detection is more uncertain because of inadequate spatial resolution (distant flyby; Fig. 22a) or long integration times (GCO5000; Fig. 22c). These predictions are preliminary due to the use of a modelled instrument response and will be refined using in-flight measurements of the instrument performance.

## Appendix: Excerpt of the JUICE Science Requirement Matrix

This Appendix presents an excerpt of the JUICE Science Requirements Matrix (SRM) included in the JUICE Science Requirements Document (SciRD, ESA Document: JUI-EST-SGS-RS-001, Issue 2, Rev 5). This SRM provides details for the codes find in the main text of the manuscript in terms of science objectives, investigations and measurement requirements expected to be achieved by JUICE at Ganymede (Table 3), Europa (Table 4), and Callisto (Table 5), respectively.

**Table 3:** Ganymede

Objectives	Investigations		Measurement Requirements	Instruments
Characterise the extent of the ocean and its relation to the deeper interior.	GA.1	Determine the amplitude and phase of the gravitational tides.	GA.1a. Measure spacecraft acceleration to resolve 2nd degree gravity field time dependence. Recover k_2_ at the orbital frequency of Ganymede with an accuracy of 5⋅10^−4^ (real) or 1⋅10^−3^ (complex) by performing range-rate measurements (accuracy ∼0.01 mm/s at 60 sec integration time) to determine spacecraft orbit to better than 1-meter (rms) over several tidal cycles. Determine the position of Ganymede’s center of mass relative to Jupiter during the lifetime of the mission to better than 10 meters, by performing range measurements with an accuracy of 20 cm end-to-end and range-rate measurements (accuracy ∼0.01 mm/s at 60 sec integration time) to determine spacecraft orbit to better than 1-meter (rms) throughout the lifetime of the orbiter.	3GM
			GA.1b. Measure topographic differences from globally distributed repeat ranging measurements, to recover spacecraft altitude at crossover points to 1-meter vertical accuracy by contiguous global ranging to the surface with 10-cm accuracy.	GALA
	GA.2	Characterise the space plasma environment to determine the magnetic induction response from the ocean.	GA.2a. Measure three-axis magnetic field components (at 32 to 128 Hz with a resolution of 0.1 nT) over a range of orbital phases to determine the induction response at multiple frequencies (related to satellite orbital and Jupiter rotation time scales).	JMAG
			GA.2b. Determine the distribution functions for electrons and ions (first order mass resolution) with an angular and energy coverage sufficient to determine the cold plasma density and velocity to constrain contributions from currents not related to the surface and ocean. Identify open and closed field lines with a latitudinal accuracy of ≤1° in Ganymede circular orbit by measuring electrons in opposing directions with a time resolution of ≤20 s.	PEP
			GA.2c. Measure electric field vectors at DC to 1.6 MHz better than <1 mV/m accuracy. Measure the vector magnetic field variations at 0.1 Hz–20 kHz. Together with electron and ion density (10^−4^ to 10^5^ /cm^3^) measurements with better than 20% accuracy, determine conductivity and electrical currents. Measure electron temperature (0.01–100 eV) and the ion drift speed (<200 km/s).	RPWI
	GA.3	Characterise surface motion over Ganymede’s tidal cycle.	GA.3a. Measure topographic differences (tidal amplitudes) with respect to the reference ellipsoid from globally distributed repeat measurements at varying orbital phase, with better than or equal to 1-meter vertical accuracy, to recover h_2_ to 0.01 (at the orbital frequency) by contiguous global ranging to the surface with 10-cm accuracy.	GALA
			GA.3b. Measure spacecraft acceleration to resolve the position of the spacecraft to better than 1-meter (rms) by performing range-rate measurements with an accuracy ∼0.01 mm/s at 60 sec integration time to determine spacecraft orbit to better than 1-meter (rms) over several tidal cycles.	3GM
			GA.3c. Provide supplementary measurements of the S/C differential lateral position relative to the ICRF2 background extragalactic radio sources with the accuracy of 100–10 μas (1 sigma) over integration time 60–1000 s.	PRIDE
	GA.4	Determine the satellite’s dynamical rotation state (forced libration, obliquity and nutation).	GA.4a. Determine the amplitude of physical longitudinal librations and the orientation of the spin pole by determining surface elevations and the orientation of the global shape at varying orbital phase of Ganymede (different true anomalies with respect to Jupiter), with up to 1-meter vertical accuracy, short along-track distance between the laser spots (few tens of meters) and by taking continuous tracks.	GALA
			GA.4b. Acquire multiple images of the same surface target at varying orbital phase, to determine satellite’s surface relative rotational position with better than 10 m horizontal accuracy. This would allow an independent evaluation of the mean spin pole, the forced nutation of the spin pole and the amplitude of the forced libration of Ganymede’s surface through fitting data with rotation models.	JANUS
			GA.4c. Perform range measurements with an accuracy of 20 cm end-to-end and range-rate measurements (accuracy ∼0.01 mm/s at 60 sec integration time) to a) determine the spacecraft position during circular orbits with an accuracy < 10 cm radial, < 10 m along track, <10 m (1–2 meters) out of plane (orbital frame of reference), to provide the appropriate referencing to laser altimeter measurements and optical imaging; and b) determine Ganymede’s physical librations in longitude and pole position in the putative body-fixed frame to accuracies about 10–20 m.	3 GM
	GA.5	Investigate the core and rocky mantle.	GA.5a. Resolve the gravity field to degree and order 12 or better by performing range-rate measurements (accuracy ∼0.01 mm/s at 60 sec integration time) to determine spacecraft orbit to better than 1-meter (rms).	3GM
			GA.5b. Perform topographic measurements to resolve coherence with gravity to degree and order 12 or better, with better than or equal to 1-meter vertical accuracy, by contiguous global ranging to the surface with 10-cm accuracy.	GALA
			GA.5c. Measure three-axis magnetic field components (at 32 to 128 Hz with a resolution of 0.1 nT) to determine whether there is a induction response at the Jupiter rotation rate.	JMAG
			GA.5d. Perform astrometric determination of the rate of change of Ganymede’s orbit by acquiring multiple images of the moon from a distance including background stars with a position accuracy of at least 1 km.	JANUS
			GA.5e. Provide supplementary measurements of the S/C differential lateral position relative to the ICRF2 background extragalactic radio sources with the accuracy of 100–10 μas (1 sigma) over integration time 60–1000 s.	PRIDE
Characterise the ice shell.	GB.1	Characterise the structure of the icy shell including its properties and the distribution of any shallow subsurface water.	GB.1a Obtain profiles of subsurface thermal, compositional and structural horizons down to a few kilometers (maximum depth from 1 to 9 km depending on the crust properties), with better than 50-km profile spacing, and with vertical resolution ranging from ∼50 meters to 1% of the target depth; Estimate subsurface dielectric properties and the density of buried scatterers in targeted regions.	RIME
			GB.1b. Obtain topography with a few tens of meters along-track resolution and better than or equal to 1 meter vertical accuracy. The maximum distance between ground-tracks (usually obtained in equatorial regions because of the polar orbit) shall be less than 5 km (goal: a few hundred meters). Determine surface roughness on the scale of 50 m or better from the laser return pulse.	GALA
	GB.2	Correlate surface features and subsurface structure to investigate near-surface and interior processes.	GB.2a. Subsurface sounding: maximum depth from 1 to 9 km depending on the crust properties), with better than 50-km profile spacing, and with vertical resolution ranging from ∼50 meters to 1% of the target depth, in order to:- Obtain profiles of subsurface dielectric horizons and structures down to a few kms - Estimate subsurface dielectric properties and the density of buried scatterers in targeted regions.- Perform globally distributed profiling of subsurface thermal, compositional and structural horizons down to a few kms.- Determine the thermal emission flux by identifying thermally-controlled subsurface horizons within the ice shell	RIME
			GB.2b. Measure topography at better than or equal to 1-km horizontal scale, better than or equal to 5-meter vertical resolution and with a few tens of meters along-track resolution, over the areas co-located with subsurface profiles sounding data.	GALA
			GB.2c. Measure surface reflectance in the wavelength range from 0.4 to greater than or equal to 5 microns of targeted features at better than or equal to 125 m/px spatial resolution, with spectral resolution sufficient to distinguish sulfuric acid hydrate from Mg- and Na-enriched salt hydrates; better than 5 nm from 0.4 to 2.0 microns and better than 10 nm from 2.0 to greater than or equal to 5 microns).	MAJIS
			GB.2d. Measure surface reflectance in the wavelength range from 0.1 to 0.2 microns of targeted features at better than or equal to 1 km/px spatial resolution, with < 2 nm from 0.1 to 0.2 microns.	UVS
			GB.2e. Perform radiometric-polarized measurements in the 600 GHz band with spatial resolution of at least 5 km and under different off-nadir angles in order to constrain the thermophysical properties of the ice, regolith and ice-regolith mixtures down to a few cm. Determine the thermal emission flux by measuring global surface thermal emission at a spatial resolution of at least 10 km/px.	SWI
			GB.2f. Perform detailed three-dimensional surface morphological characterization of targeted features through imaging at better than or equal to 30-meter horizontal scale and 15-meter vertical accuracy across selected targets. For generation of DTM for RIME data interpretation, measure topography along subsurface profiles with horizontal resolution better than 300 m/px and vertical resolution better than 50 m.	JANUS
Characterise the local environment and its interaction with the jovian magnetosphere.	GC.1	Globally characterise Ganymede’s intrinsic and induced magnetic fields, with implications for the deep interior.	GC.1a. Measure three-axis magnetic field components at 32 to 128 Hz with a resolution of 0.1 nT.	JMAG
			GC.1b. Determine the distribution functions for electrons and ions (first order mass resolution) with an angular and energy coverage sufficient to determine the cold plasma density and velocity to constrain contributions from currents not related to the surface and ocean. Determine the particle precipitation pattern by imaging the backscattered neutrals in Ganymede Circular Orbit in the tens eV to few keV range with an angular resolution ≤7°. Identify the open-closed field line boundary and its variability with an latitudinal accuracy of ≤1° in Ganymede circular orbit by measuring electrons in opposing directions with a time resolution of ≤20 s.	PEP
			GC.1c. Measure electric field vectors at DC to 1.6 MHz better than <1 mV/m accuracy. Measure the vector magnetic field variations at 0.1 Hz-20 kHz. Together with electron and ion density (10^−4^ to 10^5^ /cm^3^) measurements with better than 20% accuracy, determine conductivity and electrical currents. Measure electron temperature (0.01-100 eV) and the ion drift speed (0.1–200 km/s).	RPWI
	GC.2	Characterise the particle population within Ganymede’s magnetosphere and its interaction with Jupiter’s magnetosphere.	GC.2a. Measure three-axis magnetic field components at 32 to 128 Hz with a resolution of 0.1 nT.	JMAG
			GC.2b. Measure the distribution of bulk plasma and bulk ion drift speed with 10 second resolution; Determine electron and ion density 10^−4^–10^5^/cm^3^, electron temperature (0.01- to 100-eV), bulk ion drift speed (<200 km/s), as well as suprathermal electrons (non-Maxwellian distribution). Constrain ion temperature (0.02 to 20 eV). The acceleration of particles by Alfvén- and plasma waves requires electric field vector (DC-1.6 MHz), density fluctuations (*δ*n/n, DC-10 kHz) and magnetic field vector (0.1–20 kHz) measurements, and monitoring of radio waves (80 kHz–45 MHz).	RPWI
			GC.2c. Determine the distribution functions of electrons and ions with a sufficient angular and energy coverage to derive the moments (density, velocity, pressure) with a 10 second resolution and first order mass analysis. Determine plasma composition with the mass resolution M/ΔM greater than 20. Measure the volatile content of the exosphere, over a mass range better than 300 Daltons, mass resolution better than 500. Determine the particle precipitation pattern by imaging the backscattered neutrals in Ganymede Circular Orbit in the tens eV to few keV range with an angular resolution ≤7°.	PEP
			GC.2d. Measure Ganymede’s aurora emissions with sufficient spatial resolution to characterise the variability of the atmosphere and provide a complementary observation of the open/closed field line boundary through two-dimensional spectral-spatial images. Perform observations in the wavelength range of 120- to 200-nm to measure OI (135.6 nm, 130.4 nm) and H Ly alpha with a spectral resolution of at least 2 nm to derive information on the energy and energy flux of the incoming particles.	UVS
	GC.3	Investigate the generation of Ganymede’s aurorae.	GC.3a. Determine the distribution functions of electrons and ions with a sufficient angular and energy coverage to derive the moments (density, velocity, pressure) with a 10 second resolution and first order mass analysis. Determine the spatial-temporal variability of particle precipitation by imaging the backscattered neutrals in Ganymede Circular Orbit in the tens of eV to few keV range with an angular resolution ≤7°. Determine the variable Jovian magnetospheric environment local to Ganymede by imaging ENAs in the 10-100’s keV range produced with an angular resolution ≤10° (first order mass resolution) with a time resolution of 1 h. Measure the volatile content of the exosphere, over a mass range better than 300 Daltons, mass resolution better than 500.	PEP
			GC.3b. Measure three-axis magnetic field components at 32 Hz (required rate depends on the expected orbital velocity such that the magnetic field vector is sampled at least once per 300 km).	JMAG
			GC.3c. Perform multi-wavelength spectral and monochromatic imaging of Ganymede’s aurora in the wavelength range of 120-200 nm with a spectral resolution of ≤2 nm. Requires a temporal resolution of 1-minute and a spatial resolution ≤2.6-km/px.	UVS
			GC.3d. Perform multi-wavelength spectral and monochromatic imaging of Ganymede’s aurora in the wavelength range from 0.4 to at least 5 microns with a spectral resolution of better than or equal to 5 nm from 0.4 to 2 mm; better than or equal to 10 nm from 2 to at least 5 mm; Requires a temporal resolution of 1-minute and a spatial resolution better than 1 km/x.	MAJIS
			GC.3e. Measure electric field vectors/polarization (DC to 45 MHz), including electrostatic acceleration structures. Determine electron and ion density (10^−4^–10^5^/cm^3^), bulk ion drift speed (<200 km/s), as well as suprathermal electrons. Measure small scale density perturbations (*δ*n/n, DC to 10 kHz). Determine the presence of electrostatic and electromagnetic wave emissions of importance for the auroral energy transfer (DC to 20 kHz). Measure radio waves from auroral acceleration regions (1 kHz-45 MHz).	RPWI
	GC.4	Determine the sources and sinks of the ionosphere and exosphere.	GC.4a. Obtain two-dimensional spectral-spatial images in the wavelength range of 120 to 200 nm to determine column densities of atmospheric species at better than or equal to 1 km/px spatial resolution. Spectral resolution: ≤ 1 nm from 120 to 200 nm;Perform long-term and high-temporal-resolution monitoring in context of magnetospheric variations. Determine the composition, distribution and physical characteristics (grain-size, crystallinity, physical state) of volatile materials on the surface in the spectral range 100 –200 nm, including potential measurements of water ice, O_2_, O_3_, H_2_O_2_, carbon dioxide ices and other species. Spectral resolution: better than or equal to 2 nm. Perform stellar occultations in the wavelength range from 100 nm to 200 nm to search for absorption and/or emission signatures of atmospheric species (e.g., H_2_O, O_2_). Cover 100 to 200 nm at ≤ 1 nm resolution, and latitude and longitude resolution of ≤ 30°. Perform scans perpendicular to the limb from ∼5 km above the surface to the surface of the satellite in the wavelength range of 100–200 nm with spectral resolution of 2 nm to measure or search for emission from O (135.6 nm) and other species in the atmosphere.	UVS
			GC.4b. Obtain two-dimensional spectral-spatial images in the wavelength range of 0.4- to 5-microns to determine column densities of atmospheric species at better than or equal to 1 km/px spatial resolution. Spectral resolution: better than or equal to 5 nm from 0.4 to 2 μm; better than or equal to 10 nm from 2 to at least 5 μm. Perform long-term and high-temporal-resolution monitoring in context of magnetospheric variations. Determine the composition, distribution and physical characteristics (grain-size, crystallinity, physical state) of volatile materials on the surface in the spectral range 1–5 microns, including measurements of water ice, O_2_, O_3_, H_2_O_2_, carbon dioxide ices and other species. Spectral resolution: better than or equal to 10 nm. Perform stellar occultations in the wavelength range from 0.4 to 5.0 microns to search for absorption and/or emission signatures of atmospheric species (e.g., water and oxygen). Perform scans perpendicular to the limb from ∼5 km above the surface to the surface of the satellite in the wavelength range of 1.0 to 5.0 microns (spectral resolution better than or equal to 5 nm from 1.0 to 2.0 microns and better than or equal to 10 nm from 2.0 to greater than or equal to 5.0 microns) to measure or search for emission from O_2_ (1.27 microns), H_2_O, CO_2_ (4.26 microns) and other species in the atmosphere.	MAJIS
			GC.4c. Determination of the oxygen and hydrogen isotopic composition of surface water ice along sub-solar longitudes at about 5 km spatial resolution through observations in the 600 GHz band. Determine the vertical temperature profile from the ground up to at least 200-km altitude with about 5 km vertical resolution by measurement of $\text{H}_{2}^{16}\text{O}$ and $\text{H}_{2}^{18}\text{O}$ in the 600 GHz band. Determine the distribution of water vapour from water line observations in the 600 GHz band.	SWI
			GC.4d. Perform radio occultations to measure the neutral atmosphere and ionosphere.	3GM
			GC.4e. Measure neutrals coming off Ganymede with a mass range up to 300 Daltons and a mass resolution of up to 500. Determine the distribution functions of electrons and ions with a sufficient angular and energy coverage to derive the moments (density, velocity) with a 10 second resolution and mass resolution sufficient to distinguish between key magnetospheric and ionospheric pickup ions. Measure neutral exospheric composition with sufficient accuracy and to sufficient mass resolution to identify major volatile species with mixing ratios better than 1%. Determine the particle precipitation zones by imaging the sputtered and backscattered neutrals from the surface in the 10’s eV to few keV range with an energy resolution ≤100% and angular resolution ≤7°.	PEP
			GC.4f. Measure three-axis magnetic field components at 32 to 128 Hz with a resolution of 0.1 nT.	JMAG
			GC.4g. Determine the plasma density and temperature of the ionosphere, ion drift speeds (dynamics) in the ionosphere, and the electric field vector with accuracy better than 0.1 mV/m. Measure electron and ion density (10^−4^–10^5^/cm^3^) and electron temperature (0.01–100 eV), as well as the ion ram speed (<200-km/s). Constrain ion temperature (0- to 20- eV). Determine the ionizing Extreme Ultraviolet flux. Determine the electric field vectors (near DC to 1.6 MHz). Determine the presence of suprathermal electrons and plasma inhomogenities (*δ*n/n, 0–10 kHz). Determine energy transport by Alfvén- and plasma waves between populations, by measuring the electric (DC-45 MHz) and magnetic field (0.1 Hz–20 kHz) vectors. Monitor cut-off frequencies with distance from Ganymede of natural Jovian radio emissions (80 kHz-45 MHz).	RPWI
			GC.4h. Provide supplementary multi-static measurements of occultation radio signal for characterisation of the ionosphere and neutral atmosphere.	PRIDE
Understand the formation of surface features and search for past and present activity.	GD.1	Determine the formation mechanisms and characteristics of magmatic, tectonic, and impact landforms.	GD.1a. Determine the distributions and morphologies of surface landforms at regional and local scales. Constrain the regional and global stratigraphic relationships among landforms by determining surface color characteristics at ∼100 m/px scale in at least 3 colors with near-uniform lighting conditions and solar phase angles less than or equal to 45 degrees. Characterise selected 20-km x 20-km (or larger) areas at 10 m/px. Characterise small-scale three-dimensional surface morphology, at better than 10 m/px over targeted sites, with vertical resolution of better than or equal to 5-meter (1-meter as a goal). Requires context coverage at 10x bigger scale and not worse than 10x coarser resolution. Globally characterise the morphology at least at a spatial resolution of 400 m/px (desired 100 m/px) to provide context for higher resolution data.	JANUS
			GD.1b. Altimetric profiles over targeted sites to at least 5 meter vertical resolution and a few tens of meters along-track resolution between ground-spots. The distance between different tracks shall be less than 1 km horizontal resolution (goal: a few hundred meters).	GALA
			GD.1c. Obtain profiles of subsurface dielectric horizons and structures down to a few kilometers (maximum depth from 1 to 9 km depending on the crust properties), with better than 50-km profile spacing, and with vertical resolution ranging from ∼50 meters to ∼1% of the target depth; Estimate subsurface dielectric properties and the density of buried scatters in targeted regions.	RIME
	GD.2	Constrain global and regional surface ages.	GD.2a. Determine the distributions and morphologies of surface landforms at regional and local scales. Constrain the regional and global stratigraphic relationships among landforms by imaging at ∼100 m/px in at least 3 colors with near-uniform lighting conditions and solar phase angles less than or equal to 45 degrees. Characterise the morphology of targeted features through imaging at better than 10 m/px spatial scale.	JANUS
			GD.2b. Acquire high spatial resolution observations (better than or equal to 125 m/px) from 0.4 to 5.0 microns (spectral resolution: better than or equal to 5 nm from 400 nm to 2.0 microns; better than or equal to 10 nm from 2.0 to greater than or equal to 5 microns) on the leading hemisphere, particularly on the subjovian quadrant, with emphasis on the spectral differences between geologic features and the surrounding areas. Medium spatial resolution (2.5–10 km/px) on large areas to map leading/trailing asymmetries due to contamination by exogenic material.	MAJIS
			GD.2c. Acquire high spatial resolution observations (≤ 1 km/px) from 100 nm to 200 nm (spectral resolution: ≤ 2 nm from 100 to 200 nm) on the leading hemisphere, particularly on the subjovian quadrant, with emphasis on the spectral differences and slopes between geologic features and the surrounding areas. Medium spatial resolution (≤ 5 km/px) on large areas to map leading/trailing asymmetries due to contamination by exogenic material.	UVS
			GD.2d. Globally identify and locally characterize physical and dielectric subsurface horizons down to a few kms, (maximum depth from 1 to 9 km depending on the crust properties with vertical resolution ranging from a minimum of ∼50 meters to 1% of the target depth) by obtaining subsurface sounding profiles with better than 50 km spacing.	RIME
	GD.3	Investigate processes of erosion and deposition and their effects on the physical properties of the surface.	GD.3a. Characterise the morphology of targeted features through imaging at better than 10 m/px spatial scale.	JANUS
			GD.3b. Determine the particle precipitation zones by measuring (in-situ) the precipitating electrons and ions (major magnetospheric species) in the 10’s eV to MeV energy range, and by imaging the sputtered and backscattered neutrals from the surface in the 10’s eV to few keV range with an energy resolution ≤100% and angular resolution ≤7°. Measure sputtered exospheric products over a mass range better than 300 Daltons, mass resolution better than 500.	PEP
			GD.3c. Measure three-axis magnetic field components at 32 to 128 Hz and with a resolution of 0.1 nT to observe ion cyclotron waves; relate these waves to neutral ionization and plasma pickup in order to constrain sputtering rates.	JMAG
			GD.3d. Measure the DC electric field vector (DC-1 kHz) to monitor electrostatic acceleration structures and Alfvén waves that accelerate charged particles toward surface. Determine charge level on dust population by measuring the electron and ion densities (10^−4^–10^5^/cm^3^), and the spacecraft potential at +/−100 V at 1 V accuracy.	RPWI
			GD.3e. Perform measurements in the wavelength range of 1 to 5 microns with a spectral resolution better than or equal to 5 nm to characterise water ice bands at 1650 nm, 2000 nm, 3100 nm and 4530 nm (e.g. grain size), and hydrated salts and sulfuric acid hydrate at a spatial resolution better than 1 km.	MAJIS
Determine global composition, distribution and evolution of surface materials.	GE.1	Characterise surface organic and inorganic chemistry, including abundances and distributions of materials.	GE.1a. Identify and map non-water-ice materials (including organic compounds and radiolytic materials) over a wide range of spatial scales (from 5 km/px to 1 km/px or better), in the overall spectral range of 0.4 to greater than or equal to 5 microns and with a spectral resolution better than or equal to 5 nm from 400 nm to 2.0 microns and better than or equal to 10 nm from 2.0 to greater than or equal to 5 microns. At least 50% coverage with spatial resolution between 2 and 3 km/px. Identify globally distributed bulk material composition by measuring grain size, porosity, crystallinity, and physical state of water ice in the spectral range from 1.0 to 4.0 microns with a spectral resolution better than or equal to 10 nm and over a wide range of spatial scales (from 10 km/px to 100 m/px or better) and illumination conditions.	MAJIS
			GE.1b. Identify and map non-water-ice materials (including organic compounds and radiolytic materials) over a wide range of spatial scales (from 5 km/px to 1 km/px or better), in the overall spectral range of 100 – 200 nm and with a spectral resolution of ≤ 2 nm. At least 50% coverage with spatial resolution ≤3 km/px.	UVS
			GE.1c. Identify globally distributed bulk material composition by measuring grain size, porosity, crystallinity, and physical state of water ice from polarized continuum observations of the surface in the 600 GHz band under different off-nadir angles.	SWI
			GE.1d. Constrain the dielectric permittivity of the surface material by bistatic radar observations.	3GM
			GE.1e. Image at resolution better than 10 m/px with an image width of ∼2 km. Obtain repeat coverage to facilitate stereo analysis of targeted features.	JANUS
			GE.1f. Characterise the composition of sputtered surface products over a mass range better than 300 Daltons, mass resolution better than 500. Image the sputtered and backscattered neutrals from the surface in the 10’s eV to few keV range with an energy resolution ≤100% and angular resolution ≤7°.	PEP
			GE.1g. Provide supplementary multi-static measurements of radio occultation radio signal for estimates of dielectric properties of the surface.	PRIDE
	GE.2	Relate compositions and properties and their distributions to geology.	GE.2a. Acquire global imaging at 400 m/px in four colors (*e.g.* 0.4, 0.5, 0.7, and 0.9 μm). Obtain three-color (*e.g.* 0.4, 0.7, 0.9 μm) and monochromatic coverage for selected large areas at up to or better than 100 m/px. Acquire image mosaics at a uniform spatial resolution and illumination angle (*e.g*. mid-morning/mid-afternoon). Characterise the morphology through imaging at better than 10 m/px spatial scale over regions of high interest. For generation of DTM for RIME data interpretation, measure topography along subsurface profiles with horizontal resolution better than 300 m/px and vertical resolution better than 50 m.	JANUS
			GE.2b. Identify surface modifications due to external plasma and particle interactions by measuring (in-situ) the precipitating electrons and ions (major magnetospheric species) in the 10’s eV to MeV energy range, and by imaging the sputtered and backscattered neutrals from the surface in the 10’s eV to few keV range with an energy resolution ≤100% and angular resolution ≤7°. Measure neutral exospheric composition with sufficient sensitivity and to sufficient mass resolution to identify major volatile species with mixing ratios better than 1%.	PEP
			GE.2c. Detect dust and determine its mass and size distribution with electric field (DC to 45 MHz). Determine charge level on dust population by measuring the electron and ion densities (10^−4^–10^5^/cm^3^), and the spacecraft potential at +/−100 V at 1 V accuracy. Measure the DC electric field vector (DC-1 kHz) to monitor electrostatic acceleration structures and Alfvén waves that accelerate charged particles toward surface.	RPWI
			GE.2d. Identify and locally characterize subsurface compositional horizons and structures by obtaining profiles of subsurface dielectric horizons and structures (maximum depth from 1 to 9 km depending on the crust properties with vertical resolution ranging from a minimum of ∼50 meters to 1% of the target depth), with better than 50-km profile spacing; estimate subsurface dielectric properties and the density of buried scatterers in targeted regions.	RIME
			GE.2e. Measure topography with at least 5 meter vertical resolution and a few tens of meters along-track resolution between ground-spots. The distance between different tracks shall be less than 1 km horizontal resolution (goal: a few hundred meters) over the areas co-located with subsurface profiles.	GALA
			GE.2f. Map non-water-ice materials (including organics and products of radiolysis and ion bombardment, e.g. H_2_O_2_, O_3_, H_2_CO, H_2_CO_3_) and their association with known geologic features over the wavelength range of 100 nm to 200 nm with spectral resolution better than or equal to 2 nm. Requires at least 50% coverage with spatial resolution ≤3 km/px.	UVS
			GE.2g. Map non-water-ice materials (including potential organics and products of radiolysis and ion bombardment, e.g. H_2_O_2_, O_3_, H_2_CO, H_2_CO_3_) and their association with known geologic features over the wavelength range of 400-nm to greater than or equal to 5.0 microns with spectral resolution better than or equal to 5 nm from 400 nm to 2.0 microns; better than or equal to 10 nm from 2.0 to greater than or equal to 5.0 microns. Requires at least 50% coverage with spatial resolution between 2 and 3 km/px. Determine the origin and evolution of non-water-ice materials by making measurements in the wavelength range from 0.4 to at least 5.0-microns with a spectral resolution better than or equal to 10 nm and a spatial resolution better than or equal to 125 m/px of representative features. Co-register with higher-resolution monochromatic images at better than or equal to 100 m/px (see GE.2a).	MAJIS
	GE.3	Investigate surface composition and structure on open vs. closed field line regions.	GE.3a. Map several known or expected tracer species of weathering effects induced by the magnetosphere (e.g., H_2_O, CO_2_, NH_3_, O_3_, H_2_O_2_, H_2_SO_4_ hydrate, etc.) over the wavelength range of 100 to 200 nm with spectral resolution ≤ 5 nm. Requires at least 50% coverage with spatial resolution ≤ 3 km/px.	UVS
			GE.3b. Map several known or expected tracer species of weathering effects induced by the magnetosphere (e.g., H_2_O, CO_2_, NH_3_, O_3_, H_2_O_2_, H_2_SO_4_ hydrate, etc.) over the wavelength range of 1.0 to 2.0 μm with a spectral resolution better than or equal to 5 nm and 2.0 to at least 5 μm with a spectral resolution less than or equal to 10 nm. Requires at least 50% coverage with spatial resolution between 2 and 3 km/px. Map the distribution of the state of water ice (crystalline vs. amorphous) as a function of the latitude, in the spectral range from 1.0- to 4.0-μm with spectral resolution less than or equal to 10 nm. Map targeted features to assess local conditions (albedo) in the 0.4- to 1.0-μm range. Requires at least 50% coverage with spatial resolution between 2 and 3 km/px.	MAJIS
			GE.3c. Measure three-axis magnetic field components at 32 to 128 Hz and with a resolution of 0.1 nT at different orbital phases and distances less than 0.5 moon radii.	JMAG
			GE.3d. Global monochromatic imaging at 400 m/px with selected features mapped in 4 colors (e.g. 0.4-μm, 0.5-μm, 0.7-μm, 0.9-μm) at 100 m/px. Targeted imaging at a resolution of better than 30 m/px over regions of interest.	JANUS
			GE.3e. Identify open and closed field lines with an latitudinal accuracy of ≤1° in Ganymede circular orbit by measuring electrons in opposing directions with a time resolution of ≤20 s. Measure neutral exospheric composition with sufficient sensitivity and to sufficient mass resolution to identify major volatile species with mixing ratios better than 1%. Determine the particle precipitation zones by measuring (in-situ) the precipitating electrons and ions (major magnetospheric species) in the 10’s eV to MeV energy range, and by imaging the sputtered and backscattered neutrals from the surface in the 10’s eV to few keV range with an energy resolution ≤100% and angular resolution ≤7°.	PEP
			GE.3f. Measure electron and ion density (10^−4^–10^5^/cm^3^) and electron temperature (0 to 100 eV), as well as constrain ion temperature (0 to 20 eV) on open versus closed field lines. Determine charge level on dust population by measuring the electron and ion densities (10^−4^–10^5^/cm^3^), and the spacecraft potential at +/−100 V at 1 V accuracy. Measure the DC electric field vector (DC-1 kHz) to monitor electrostatic acceleration structures and Alfvén waves that accelerate charged particles toward surface.	RPWI
	GE.4	Determine volatile content to constrain satellite origin and evolution.	GE.4a. Measure the stable isotopes of C, H, O, and N in the major volatiles (*e.g*. H_2_O, CH_4_, NH_3_, CO, CO_2_, SO_2_), and measure the noble gases Ar, Kr, and Xe, with mass resolution better than 500. Characterise the composition of sputtered desorbed volatiles over a mass range better than 300 Daltons with mass resolution better than 500.	PEP
			GE.4b. and the ^17^O/^16^O and ^18^O/^16^O ratio in water ice from limb observations of water rotational transitions in the 600 GHz band. Identify globally distributed bulk material composition from polarized continuum observations of the surface in the 600 GHz band under different off-nadir angles.	SWI
			GE.4c. Identify and map non-water-ice materials over a wide range of spatial scales (from 5 km/px to 1 km/px or better), in the overall spectral range 0.1–0.2 micron and with a spectral resolution better than or equal to 2 nm, suitable to discriminate various volatiles known or expected to exist on the surface. Requires at least 50% coverage with spatial resolution ≤ 3 km/px.	UVS
			GE.4d. Identify and map non-water-ice materials over a wide range of spatial scales (from 5 km/px to 1 km/px or better), in the overall spectral range 0.4 to greater than or equal to 5 μm and with a spectral resolution better than or equal to 5 nm from 0.4 to 2.0 μm and better than or equal to 10 nm from 2.0 to greater than or equal to 5 μm, suitable to discriminate various volatiles known or expected to exist on the surface. Requires at least 50% coverage with spatial resolution between 2 and 3 km/px. Identify globally distributed bulk material composition in the spectral range from 1.0 to at least 5.0 μm with a spectral resolution better than or equal to 10 nm and over a wide range of spatial scales (from 10 km/px to 100 m/px or better).	MAJIS

**Table 4:** Europa

Science Objectives	Science Investigations		Measurement Requirements	Instruments
EA. Determine the composition of the non-ice material, especially as related to habitability	EA.1	Characterise surface organic and inorganic chemistry, including abundances and distributions of materials, with emphasis on essential elements for habitability and potential biosignatures.	EA.1a. Measure surface reflectance of large areas across the dayside, at a spatial resolution between 2.5 and 10 km/px, through a spectral range from 0.4 to 2.0 μm with a spectral resolution of better than or equal to 5 nm, and from 2.0 μm to at least 5 μm with a spectral resolution better than or equal to 10 nm. Targeted characterization of selected sites of very high interest should be at a spatial resolution of better than or equal to 100 m-1 km/px.	MAJIS
			EA.1b. Measure surface reflectance of large areas across the dayside, at a spatial resolution <50 km/px, through a spectral range of at least 110 to 200 nm with ≤ 3-nm spectral resolution. Targeted characterization of selected sites of very high interest should be at a spatial resolution of better than or equal to ∼1 km/px.	UVS
			EA.1c. Characterise the composition of sputtered surface products over a mass range better than 300 Daltons, mass resolution better than 500. Image the sputtered and backscattered neutrals from the surface in the 10’s eV to few keV range with an energy resolution ≤100% and angular resolution ≤7°.	PEP
			EA.1d. Correlate surface composition and physical characteristics (e.g., grain size) with geologic features through 3-color mapping at resolution of better than 2 km/px over the dayside, including selected sites of very high interest at spatial resolutions better than 0.5 km/px.	JANUS
	EA.2	Relate material composition and distribution to geological features and geological processes, especially material exchange with the interior.	EA.2a. Measure surface reflectance of large areas across the dayside, at a spatial resolution between 2.5 and 10 km/px, through a spectral range from 0.4 to 2.0 μm with a spectral resolution of better than or equal to 5 nm, and from 2.0 μm to at least 5 μm with a spectral resolution better than or equal to 10 nm. Targeted characterization of selected sites of very high interest should be at a spatial resolution of better than or equal to ∼100 m-1 km/px.	MAJIS
			EA.2b. Measure surface reflectance of large areas across the dayside, at a spatial resolution <50 km/px, through a spectral range of at least 110 to 200 nm with ≤ 3-nm spectral resolution. Targeted characterization of selected sites of very high interest should be at a spatial resolution of better than or equal to ∼1 km/px.	UVS
			EA.2c. Characterize subsurface dielectric horizons and structures down to a few kms (maximum depth from 1 to 9 km depending on the crust properties and with vertical resolution ranging from ∼50 meters to 1% of the target depth) by means of subsurface profiles of selected sites of at least 30-km in length.	RIME
			EA.2d. Determine the distributions and morphologies of surface landforms at regional and local scales, through monochromatic imaging with resolution better than or equal to 0.5 km/px. Constrain regional and global stratigraphic relationships by determining surface color characteristics at better than 2 km/px scale in at least 3 colors with near-uniform lighting conditions and solar phase angles less than or equal to 45 degrees, over large areas across the dayside. Measure topography on the order of 0.5 km/px spatial scale and better than or equal to 50 meter vertical resolution over regions of high interest, and along subsurface profiles for generation of DTM for RIME data interpretation.	JANUS
			EA.2e. In regions co-located with subsurface profiles: Acquire precise topography with a vertical resolution of 1 m along-track at 100-meter (goal: a few tens of meters) horizontal resolution. Determine surface roughness on the scale of 80 to 40 m or better during flybys from the laser return pulse.	GALA
			EA.2f. Perform radiometric-polarized measurements in the 600 GHz band and under different off-nadir angles to constrain the thermophysical properties of the ice, regolith and ice-regolith mixtures down to a depth of a few cm.	SWI
			EA.2g. Measure the volatile content of potential outgassing sources from the shallow subsurface or deeper interior and relate them to the origin and evolution of the satellite, over a mass range better than 300 Daltons, mass resolution better than 500.	PEP
	EA.3	Characterization of the backscattered and sputtered material from the surface	EA.3a. Measure the stable isotopes of C, H, O, and N in the major volatiles (*e.g*. H_2_O, CH_4_, NH_3_, CO, CO_2_, SO_2_), measure the noble gases Ar, Kr, and Xe, with mass resolution better than 500, and characterise the composition of sputtered desorbed volatiles over a mass range better than 300 Daltons and mass resolution better than 500. Determine the particle precipitation by measuring (in-situ) electrons and ions (major magnetospheric species) in the 10’s eV to MeV energy range. Image the sputtered and backscattered neutrals from the surface in the 10’s eV to few keV range with an energy resolution ≤100% and angular resolution ≤7°.	PEP
			EA.3b. Determine the plasma waves and cold plasma characteristics with good time resolution (<10 s). Determine mass and size distribution of dust for sizes >1 μm. Measure electron and ion density (10^−4^–10^5^/cm^3^) and electron temperature (0.01-100 eV), as well as the ion ram speed (<200-km/s). Constrain ion temperature (<20 eV). Determine the presence of suprathermal electrons and plasma inhomogenities (dn/n, 0–10 kHz). Determine energy transport by Alfvén- and plasma waves between populations, by measuring the electric (DC–45 MHz) and magnetic field (0.1 Hz–20 kHz) vectors.	RPWI
			EA.3c. Measure three-axis magnetic field components at 32 to 128 Hz and with a resolution of 0.1 nT to observe ion cyclotron waves; relate these waves to neutral ionization and plasma pickup in order to constrain sputtering rates.	JMAG
			EA.3d. Measure ionospheric electron density profiles for different locations and infer correlations with solar zenith angle, magnetospheric location and surface characteristics.	3GM
			EA.3e. Measure surface reflectance of large areas across the dayside of Europa, at a spatial resolution <50 km/px, through a spectral range of at least 110 to 200 nm, with ≤ 3 nm spectral resolution, along with targeted characterization of selected sites of very high interest at a spatial resolution of better than or equal to ∼1 km/px.	UVS
			EA.3f. Determine the distributions and morphologies of surface landforms at regional and local scales, through monochromatic imaging with resolution better than or equal to 0.5 km/px. Constrain regional and global stratigraphic relationships by determining surface color characteristics at better than 2 km/px scale in at least 3 colors with near-uniform lighting conditions and solar phase angles less than or equal to 45 degrees, over large areas across the dayside. Determine surface color characteristics at ∼500 m/px scale in at least 3 colors.	JANUS
			EA.3g. Measure surface reflectance of large areas across the dayside, at a spatial resolution between 2.5 and 10 km/px, from 0.4 to 2.0 μm with a spectral resolution of better than or equal to 5 nm, and from 2.0 μm to greater than or equal to 5 μm with a spectral resolution better than or equal to 10 nm, along with targeted characterization of selected sites of very high interest at a spatial resolution of better than or equal to ∼1 km/px.	MAJIS
EB. Look for liquid water under the most active sites	EB.1	Surface and Subsurface exploration of the icy crust below young and resurfaced areas to look for water reservoirs	EB.1a. Identify and locally characterise subsurface thermal or compositional horizons and structures related to the current or recent presence of water or brine, by obtaining profiles of subsurface dielectric horizons and structures down to a few kms (maximum depth from 1 to 9 km depending on the crust properties and with vertical resolution ranging from ∼50 meters to 1% of the target depth).	RIME
			EB.1b. In regions co-located with subsurface profiles: Acquire precise topography with a vertical resolution of 1 m along-track at 100-meter (goal: a few tens of meters) horizontal resolution.	GALA
			EB.1c. Determine the distributions and morphologies of surface landforms at regional and local scales, through monochromatic imaging with resolution better than or equal to 0.5 km/px. In regions co-located with subsurface profiles: Measure topography on the order of 0.5 km/px spatial scale and better than or equal to 50-meter vertical resolution.	JANUS
	EB.2	Determine minimal thickness of the icy crust on most active regions	EB.2a. Identify and locally characterise subsurface thermal or compositional horizons and structures related to the current or recent presence of water or brine, by obtaining profiles of subsurface dielectric horizons and structures down to a few kms (maximum depth from 1 to 9 km depending on the crust properties and with vertical resolution ranging from ∼50 meters to 1% of the target depth).	RIME
			EB.2b. In regions co-located with subsurface profiles: 1) Acquire precise topography with a vertical resolution of 1 m along-track at 100-meter (goal: a few tens of meters) horizontal resolution.	GALA
			EB.2c. In regions co-located with subsurface profiles: Measure topography on the order of 0.5 km/px spatial scale and better than or equal to 50-meter vertical resolution.	JANUS
	EB.3	Search for possible active regions on the surface of Europa (plumes etc.)	EB.3a. Identify and locally characterise subsurface thermal or compositional horizons and structures related to the current or recent presence of water or brine, by obtaining profiles of subsurface dielectric horizons and structures down to a few kms (maximum depth from 1 to 9 km depending on the crust properties and with vertical resolution ranging from ∼50 meters to 1% of the target depth).	RIME
			EB.3b. Characterise the detailed three-dimensional surface morphology of targeted features at a few 10s of meters horizontal scale and 1 meter vertical accuracy across the target, co-located with subsurface profiles.	GALA
			EB.3c. Determine surface color characteristics at ∼500 m/px scale in at least 3 colors, colocated with subsurface profiles. Measure topography on the order of 0.5 km/px spatial scale and better than or equal to 50 meter vertical resolution over regions of high interest.	JANUS
			EB.3d. Determine the distribution function of electrons and ions with sufficient angular and energy coverage to derive plasma density and velocity. Measure ion composition with first-order mass resolution. Measure the volatile content of potential outgassing sources from the shallow subsurface or deeper interior and relate them to the origin and evolution of the satellite, over a mass range better than 300 Daltons, mass resolution better than 500.	PEP
			EB.3e. Measure three-axis magnetic field components at 32 to 128 Hz and with a resolution of 0.1 nT to observe ion cyclotron waves; relate these waves to neutral ionization and plasma pickup in order to detect and constrain neutral sources.	JMAG
			EB.3f. Determine plasma wave, electrodynamics and cold plasma characteristics with good time resolution (< 10 s). This requires the full range of measurements of electric field vectors (DC-45 MHz), magnetic field vectors (0.1-20 kHz), density perturbations (*δ*n/n, DC–10 kHz), electron and ion densities (10^−4^–10^5^/cm^3^), electron temperature (0.01-100 eV), ion drift speed (<200 km/s), spacecraft potential (+/−100 V), and the mass and size distribution of dust for dust >1 μm. The DC electric field measurements need an accuracy better than 1 mV/m.	RPWI
			EB.3g. Search for high gradients in the water exosphere near the surface by measuring the rotational ground state of water vapour at 557 GHz	SWI
EC. Study the active processes	EC.1	Study the interaction between the local environment and (a) the Europa torus, (b) the effects of radiation on surface chemistry, and (c) sputtering processes	EC.1a. (a) Determine the global, relative variability and distribution of Europa neutral torus (and possible plasma component) through remote ENA imaging in the 10’s eV - 100 keV range (first order mass resolution) with ≤7° angular resolution on 10 h time scales. Determine the local pitch-angle distributions of ions in the 10-100’s keV range of major magnetospheric ion species. Measure plasma electron densities. (b)-(c) Determine the particle precipitation by measuring (in-situ) the electrons and ions (major magnetospheric species) in the 10’s eV to MeV energy range, and by imaging the sputtered and backscattered neutrals from the surface in the 10’s eV to few keV range with an energy resolution ≤100% and angular resolution ≤7°.	PEP
			EC.1b. Measure three-axis magnetic field components at 32 Hz and obtain 180 degrees plasma pitch angle coverage with time resolution of seconds to determine temporal and spatial changes of field-aligned vs. trapped particle distributions.	JMAG
			EC.1c. Determine plasma wave, electrodynamics and cold plasma characteristics with good time resolution (< 10 s). This requires the full range of measurements of electric field vectors (DC-45 MHz), magnetic field vectors (0.1-20 kHz), density perturbations (*δ*n/n, DC-10 kHz), electron and ion densities ($10^{-4}\text{--}10^{5}\text{ /cm}^{\underline{3}}$), electron temperature (0.01-100 eV), ion drift speed (<200 km/s), spacecraft potential (+/−100 V), and the mass and size distribution of dust for dust >1 μm. The DC electric field measurements need an accuracy of 1 mV/m.	RPWI
			EC.1d. Determine surface color characteristics at ∼500 m/px scale in at least 3 colors. Global view by imaging of the sodium exosphere with a spatial resolution of better than 20 km/px	JANUS
	EC.2	Observe the limb for activity	EC.2a. Perform stellar occultations and measure atmospheric emissions plus surface reflectance at better than or equal to 1 km/px spatial resolution in the wavelength range of 100 to 200 nm with < 2 nm spectral resolution. Obtain limb views for stellar occultations, including bulk photometric light curves for plume searches, and obtain surface profiles with less than or equal to 25-km spacing. Perform scans perpendicular to the limb from ∼5 km above the surface to the surface of the satellite in the wavelength range of 100 nm to 200 nm (spectral resolution of 2 nm) to measure or search for emission from O (135.6 nm) and other species in the atmosphere.	UVS
			EC.2b. Perform scans perpendicular to the limb from ∼5 km above the surface to the surface of the satellite in the wavelength range of 0.4 micron to greater than 5.0 microns (spectral resolution better than or equal to 5 nm below 2.0 microns and better than or equal to 10 nm from 2.0 to at least 5.0 microns) to measure or search for emission from O_2_ (1.27 microns), H_2_O, CO_2_ (4.26 microns) and other species in the atmosphere.	MAJIS
			EC.2c. Determine the distribution of water vapour from water line observations in the 600 GHz band.	SWI
	EC.3	Look for surface changes w.r.t. Galileo observations	EC.3a. Detailed morphological characterization of targeted features at better than or equal to 50 m/px spatial scale, and solar incidence angles between 30° and 70°, ideally with stereo. Co-located with imaging at approximately 10 times wider swath and not worse than 10 times coarser resolution. Correlate surface composition and physical characteristics (e.g., grain size) with geologic features through 3-color mapping at resolution of better than 2 km/px over the dayside of Europa, including selected sites of very high interest at spatial resolutions better than 0.5 km/px.	JANUS
			EC.3b. Measure surface reflectance of large areas across the dayside of Europa, at a spatial resolution <50 km/px, in the UV	UVS
			EC.3c. Measure surface reflectance of large areas across the dayside of Europa, at a spatial resolution between 2.5 and 10 km/px, from the Vis to the IR.	MAJIS

**Table 5:** Callisto

Objectives	Investigations		Measurement Requirements	Instruments
CA. Characterise the outer shells, including the ocean.	CA.1	Explore the structure and properties of Callisto’s icy crust and liquid shell.	CA.1a. Measure subsurface dielectric properties and estimate density of buried scatterers in targeted regions by obtaining profiles of subsurface dielectric horizons and structures down to a few kms (maximum depth from 1 to 9 km depending on the crust properties and with vertical resolution ranging from ∼50 meters to 1% of the target depth).	RIME
			CA.1b. In regions co-located with subsurface profiles: Acquire precise topography at 1 meter vertical resolution along-track at 100-meter (goal: a few tens of meters) horizontal resolution.	GALA
			CA.1c. Measure topography on the order of 0.5 km/px spatial scale and better than or equal to 50 meter vertical resolution over regions of high interest and along subsurface profiles for generation of DTM for RIME data interpretation.	JANUS
	CA.2	Characterise the space plasma environment to determine the magnetic induction response from Callisto’s ocean.	CA.2a. Measure three-axis magnetic field components at 32 to 128 Hz with a resolution of 0.1 nT across multiple flybys at different orbital phases to determine the induction response at multiple frequencies (related to satellite orbital and Jupiter rotation time scales).	JMAG
			CA.2b. Determine the distribution functions for electrons and ions (first order mass resolution) with an angular and energy coverage sufficient to determine the cold plasma density and velocity to constrain contributions from currents not related to the surface and ocean.	PEP
			CA.2c. Measure electric field vectors from DC to 1.6 MHz better than <1 mV/m accuracy. Measure the vector magnetic field variations at 0.1 Hz-20 kHz. Together with electron and ion density (10^−4^ to 10^5^/cm^3^) measurements with better than 20% accuracy, determine conductivity and electrical currents. Measure electron temperature (0.01-100 eV) and the ion drift speed (<200 km/s).	RPWI
CB. Determine the composition of the non-ice material	CB.1	Characterise surface organic and inorganic chemistry, including abundances and distributions of materials and volatile outgassing.	CB.1a. Identify and map non-water-ice materials (including organic compounds and radiolytic materials) over a wide range of spatial scales (from 5 km/px to 100 m/px or better), with a spectral resolution (better than or equal to 5 nm from 0.4 to 2.0 μm and better than or equal to 10 nm from 2.0 to greater than or equal to 5 μm) suitable to discriminate various compounds known or expected to exist on the surface. Determine bulk material composition, grain size, porosity, crystallinity, and physical state of water ice in the spectral range from 1 to 4 microns with a spectral resolution better than or equal to 10 nm over a wide range of spatial scales (from 10 km/px to 100 m/px or better) and illumination conditions. Determine the composition, distribution and physical characteristics (grain-size, physical state) of volatile materials on the surface. Determine the origin and geologic evolution of non-water-ice materials, including the role of geologic processes by making observations in the wavelength range from 0.4 to at least 5 μm with a spectral resolution better than or equal to 10 nm and spatial resolution better than or equal to 1 km/px of representative features. Observations need to be co-registered with higher-resolution panchromatic images.	MAJIS
			CB.1b. Acquire two-dimensional spectral-spatial images in the range 100 to 200 nm with a spectral resolution better than or equal to 2 nm and a spatial resolution from 5 km/px to 1 km/px or better. Determine the composition, distribution and physical characteristics (grain-size, physical state) of volatile materials on the surface, including measurements in the wavelength range of 100- to 200 nm at 2 nm spectral resolution to identify O_3_, H_2_O_2_ and other species.	UVS
			CB.1c. Measure the volatile content (*i.e.* water, carbon dioxide, methane, ammonia, and noble gases) of potential outgassing sources from the near subsurface or deeper interior over a mass range better than 300 Daltons with a mass resolution better than 500. Characterise the composition of sputtered surface products over a mass range better than 300 Daltons, mass resolution better than 500. Image the sputtered and backscattered neutrals from the surface in the 10’s eV to few keV range with an energy resolution ≤100% and angular resolution ≤7°.	PEP
			CB.1d. Image at medium-resolution (∼100’s m/px) to characterize large parts of the surface in four spectral band passes (e.g. 0.4, 0.67, 0.76, 1.0 μm) in the wavelength range of 350 nm to 1.0 μm including multiphase coverage for measurements of surface physical properties. Acquire high-resolution (better than 50 m/px) imaging of selected targets. Requires repeat pass coverage of areas of interest to assess temporal variations. Determine the origin and geologic evolution of non-water-ice materials, including the role of geologic processes by making high-resolution panchromatic images (<1 km/pxl) of representative features.	JANUS
			CB.1e. Identify globally distributed bulk material composition from polarized continuum observations of the surface in the 600 GHz band under different off-nadir angles.	SWI
			CB.1f. Characterize the ionized part of the volatile outgassing by measuring the electron and ion densities (10^−4^–10^5^/cm^3^) and ion drift speed (<200 km/s) close to Callisto with good time resolution (< 10 s). This requires also measurements of electric field vectors (DC–45 MHz), magnetic field vectors (0.1–20 kHz), density perturbations (*δ*n/n, DC–10 kHz), electron temperature (0.01-100 eV), spacecraft potential (+/−100 V), and the mass and size distribution of dust for dust >1 μm. The DC electric field measurements need an accuracy better than 1 mV/m.	RPWI
	CB.2	Relate material composition and distribution to geological and magnetospheric processes.	CB.2a. Map large parts of the surface at medium resolution (∼100’s m/px) in four spectral band passes (*e.g.* 0.4 μm, 0.67 μm, 0.76 μm, 1.0 μm) in the wavelength range of 350 nm to 1.0 μm.	JANUS
			CB.2b. Identify surface modifications due to external plasma and particle interactions by measuring (in-situ) the precipitating electrons and ions (major magnetospheric species) in the 10’s eV to MeV energy range, and by imaging the sputtered and backscattered neutrals from the surface in the 10’s eV to few keV range with an energy resolution ≤100% and angular resolution ≤7°. Determine the variable Jovian magnetospheric environment local to Ganymede by imaging ENAs in the 10-100’s keV range produced with an angular resolution ≤10° (first order mass resolution) with a time resolution of 1 h. Measure neutral exospheric composition with sufficient sensitivity and to sufficient mass resolution to identify major volatile species with mixing ratios better than 1%. Requires open source positive ion spectrum to characterize the composition of ionospheric plasma, open source neutral spectrum for density profiles of sputtered species, and closed source neutral spectrum.	PEP
			CB.2c. Measure the surface reflectance in the wavelength range from 0.4 to at least 5 μm to identify surface composition and relate it to geologic units and weathering processes. Identify and map the distribution of products of radiolysis and ion bombardment (*e.g.* H_2_O_2_, O_3_, H_2_CO, H_2_CO_3_) over the wavelength range from 1.0 to 2.0 μm at better than or equal to 5 nm spectral resolution and from 2.0 to greater than or equal to 5 μm at better than or equal to 10 nm spectral resolution.	MAJIS
			CB.2d. Measure the surface reflectance in the wavelength range from 0.1 to 0.2 μm to identify surface composition and relate it to geologic units and weathering processes. Identify and map the distribution of products of radiolysis and ion bombardment (*e.g.* H_2_O_2_, O_3_, H_2_CO, H_2_CO_3_) over the wavelength range of 100 to 200 nm with spectral resolution better than or equal to 2 nm.	UVS
			CB.2e. Perform radiometric-polarized measurements in the 600 GHz band and under different off-nadir angles to constrain the thermophysical properties of the ice, regolith and ice-regolith mixtures down to a depth of a few cm.	SWI
			CB.2f. Detect dust and determine its mass and size distribution with electric field (DC to 45 MHz). Determine charge level on dust population by measuring the electron and ion densities (10^−4^–10^5^/cm^3^), and the spacecraft potential at +/−100 V at 1 V accuracy. Measure the DC electric field vector (DC-1 kHz) to monitor electrostatic acceleration structures and Alfvén waves that accelerate charged particles toward surface.	RPWI
			CB.2g. In regions co-located with subsurface profiles: Acquire precise topography at 1 meter vertical resolution along-track at 100-meter (goal: a few tens of meters) horizontal resolution.	GALA
			CB.2h. Measure subsurface dielectric properties in targeted regions by obtaining profiles of subsurface dielectric horizons and structures down to a few kms (maximum depth from 1 to 9 km depending on the crust properties and with vertical resolution ranging from ∼50 meters to 1% of the target depth).	RIME
	CB.3	Characterize the ionosphere and exosphere of Callisto.	CB.3a. Identify and determine column densities of atmospheric species across the globe at 1 km spatial resolution or better using stellar occultations. Requires coverage in the wavelength ranges of 0.4 to 2.0 μm with spectral resolution better than or equal to 5 nm and 2.0 to 5.0 μm with a spectral resolution better than or equal to 10 nm. Obtain global, two-dimensional, spectral-spatial images at better than 50 km/px spatial resolution in the wavelength range of 0.1- to 5-microns to measure CO_2_, C, O, CO, O^+^ and other species in absorption and/or emission. spectral resolution better than 10 nm for wavelengths greater than 1.0-micron. Map atmospheric emissions by scanning perpendicular to the limb from ∼300 km above the surface to the surface of the satellite (at steps of 5 km). Requires measurements in the wavelength range 1.0 to 2.0 μm at better than or equal to 5 nm from and 2.0 to 5 μm at better than or equal to 10 nm.	MAJIS
			CB.3b. Identify and determine column densities of atmospheric species across the globe at 1 km spatial resolution or better using stellar occultations. Requires coverage in the wavelength ranges of 100- to 200- nm at 1 nm spectral resolution. Obtain global, two-dimensional, spectral-spatial images at better than 50 km/px spatial resolution in the wavelength range of 100- to 200-nm to measure CO_2_, C, O, CO, O^+^ and other species in absorption and/or emission. Requires spectral resolution of 2 nm. Map atmospheric emissions by scanning perpendicular to the limb from ∼300 km above the surface to the surface of the satellite (at steps of 5 km). Requires measurements in the wavelength range of 100 to 200 nm at 2 nm spectral resolution.	UVS
			CB.3c. Measure neutral exospheric composition with sufficient accuracy and to sufficient mass resolution to identify major volatile species with mixing ratios better than 1%. Determine positive ion spectrum of sputtered ions with M/ΔM ≥ 20. Determine the particle precipitation distribution by measuring (in-situ) the precipitating electrons and ions (major magnetospheric species) in the 10’s eV to MeV energy range,and by imaging the sputtered and backscattered neutrals from the surface in the 10’s eV to few keV range with an energy resolution ≤100% and angular resolution ≤7°.	PEP
			CB.3d. Determine the plasma density and temperature of the ionosphere, ion drift speeds (dynamics) in the ionosphere, and the electric field vector with accuracy better than 0.1 mV/m. Measure electron and ion density (10^−4^–10^5^/cm^3^) and electron temperature (0.01-100 eV), as well as the ion ram speed (<200-km/s). Constrain ion temperature (0- to 20- eV). Determine the ionizing Extreme Ultraviolet flux. Determine the electric field vectors (near DC to 1.6 MHz). Determine the presence of suprathermal electrons and plasma inhomogeneities (dn/n, 0–10 kHz). Determine energy transport by Alfvén- and plasma waves between populations, by measuring the electric (DC-45 MHz) and magnetic field (0.1 Hz–20 kHz) vectors.	RPWI
			CB.3e. Measure three-axis magnetic field components at 32 to 128 Hz with a resolution of 0.1 nT across multiple flybys at different orbital phases.	JMAG
			CB.3f. Determine the distribution of water vapour in the atmosphere from water line observations in the 600 GHz band. Determine the hydrogen and oxygen isotopic ratios of water.	SWI
			CB.3g. Characterize and map the ionosphere by performing radio occultations over as wide a range of longitude space as possible.	3GM
CC. Study the past activity	CC.1	Determine the formation and characteristics of tectonic and impact landforms.	CC.1a. Acquire precise topography at 1 m vertical resolution and better than 1 km horizontal resolution with selected targets at 100-meter (goal: a few tens of meters) horizontal resolution. In regions co-located with subsurface profiles: Acquire precise topography at 1 meter vertical resolution along-track at 100-meter horizontal resolution.	GALA
			CC.1b. Characterize large parts of the surface at medium resolution (∼100’s m/px) in four spectral band passess (e.g. 0.4 μm, 0.67 μm, 0.76 μm, 1.0 μm) in the wavelength range of 350 nm to 1.0 μm. Requires solar illumination at mid-morning to mid-afternoon local times. Acquire high-resolution (better than 50 m/px) imaging of selected targets. Measure topography on the order of 0.5 km/px spatial scale and better than or equal to 50 meter vertical resolution over regions of high interest.	JANUS
			CC.1c. Measure surface reflectance in the wavelength range from 0.4 to greater than or equal to 5 μm with spectral resolution better than or equal to 5 nm from 0.4 to 2.0 μm and better than or equal to 10 nm from 2.0 to greater than or equal to 5 μm and spatial resolution better than or equal to 20 km/px. Requires targeted high spatial/high spectral observations of important geologic units and terrain types.	MAJIS
			CC.1d. Identify and locally characterize subsurface compositional horizons and structures by obtaining profiles of subsurface dielectric horizons and structures down to a few kms (maximum depth from 1 to 9 km depending on the crust properties and with vertical resolution ranging from a minimum of ∼50 meters to 1% of the target depth) to estimate subsurface dielectric properties and the density of buried scatterers in targeted regions.	RIME
	CC.2	Investigate the interior of Callisto, with a special emphasis on its degree of differentiation.	CC.2a. Derive information on the static shape by measuring range to the surface during multiple fly-bys.	GALA
			CC.2b. Image the limb for shape determination by acquiring multiple images at better than 1 km/px resolution. Perform astrometric determination of the rate of change of Callisto’s orbit by acquiring multiple images of Callisto from a distance including background stars with a position accuracy of at least 1 km. Determine the mean spin pole direction (obliquity) to better than 1 km by developing a geodetic control network (∼20 points) at a resolution better than 500 m/px.	JANUS
			CC.2c. Determine the static gravity field at low order by measuring the range-rate from spacecraft tracking during multiple flybys to derive the 2nd degree gravity field and local potential anomalies. Requires range-rate measurements with an accuracy ∼0.01 mm/s over 60 s integration time. If tracking is available for all the flybys, the gravity field may be stimated up to degree 3 and the Love number k_2_ with an accuracy of 6E-2	3GM
			CC.2d.	
	CC.3	Constrain global and regional surface ages.	CC.3a. High spatial resolution observations (better than or equal to 1 km/px) from 0.4 to 5 μm (spectral resolution: better than or equal to 5 nm from 0.4 to 2.0 μm, better than or equal to 10 nm from 2.0 to greater than or equal to 5 μm), with emphasis on the spectral differences between geologic features (multi-ring basins, craters) and the surrounding areas. Medium spatial resolution (better than or equal to 10 km/px) on large areas to map leading/trailing asymmetries.	MAJIS
			CC.3b. Perform detailed morphological characterization of selected features through imaging at better than or equal 50 m/px spatial scale. Determine the distribution and morphology of impact craters by mapping in four colors (visible to near-IR) for large areas at scales ∼400 m/px and in a single color at regional scales (∼100 m/px) with near-uniform lighting conditions and solar phase angles less than or equal to 45 degrees.	JANUS
			CC.3c. High spatial resolution observations (better than or equal to 1 km/px) from 0.1 to 0.2 micron (spectral resolution: ≤ 2 nm), with emphasis on the spectral differences between geologic features (multi-ring basins, craters) and the surrounding areas. Medium spatial resolution (better than or equal to 10 km/px) on large areas to map leading/trailing asymmetries.	UVS
			CC.3d. Identify and locally characterize subsurface compositional horizons and structures by obtaining profiles of subsurface dielectric horizons and structures down to a few kms (maximum depth from 1 to 9 km depending on the crust properties and with vertical resolution ranging from ∼50 meters to 1% of the target depth) to estimate subsurface dielectric properties and the density of buried scatterers in targeted regions.	RIME
			CC.3e. In regions co-located with subsurface profiles: Acquire precise topography at 1 meter vertical resolution along-track at 100-meter (goal: a few tens of meters) horizontal resolution.	GALA

## References

[CR1] Alday J, Roth L, Ivchenko N, Retherford KD, Becker TM, Molyneux P, Saur J (2017) New constraints on Ganymede’s hydrogen corona: analysis of Lyman- emissions observed by HST/STIS between 1998 and 2014. Planet Space Sci 148:35–44. 10.1016/j.pss.2017.10.006 10.1016/j.pss.2017.10.006

[CR2] Arnold H, Liuzzo L, Simon S (2020) Plasma interaction signatures of plumes at Europa. J Geophys Res Space Phys 125(1):e2019JA027346. 10.1029/2019JA027147 10.1029/2019JA027147

[CR3] Aydin A (2006) Failure modes of the lineaments on Jupiter’s moon, Europa: implications for the evolution of its icy crust. J Struct Geol 28(12):2222–2236. 10.1016/j.jsg.2006.08.003 10.1016/j.jsg.2006.08.003

[CR4] Baby NR, Kenkmann T, Stephan K, Wagner R (2024) Polygonal impact craters on Ganymede. Meteorit Planet Sci 59(3):544–559. 10.1111/maps.14138 10.1111/maps.14138

[CR5] Barr AC, Stillman DE (2011) Strain history of ice shells of the Galilean satellites from radar detection of crystal orientation fabric. Geophys Res Lett 38(6):L06203. 10.1029/2010GL046616 10.1029/2010GL046616

[CR6] Barth CA, Hord CW, Stewart AIF, Pryor WR, Simmons KE, McClintock WE, Ajello JM, Naviaux KL, Aiello JJ (1997) Galileo ultraviolet spectrometer observations of atomic hydrogen in the atmosphere at Ganymede. Geophys Res Lett 24(17):2147–2150. 10.1029/97GL01927 10.1029/97GL01927

[CR7] Belgacem I, Schmidt F, Jonniaux G (2020) Regional study of Europa’s photometry. Icarus 338:113525. 10.1016/j.icarus.2019.113525 10.1016/j.icarus.2019.113525

[CR8] Bjonnes E, Johnson BC, Silber EA, Singer KN, Evans AJ (2022) Ice shell structure of Ganymede and Callisto based on impact crater morphology. J Geophys Res, Planets 127(4):e2021JE007028. 10.1029/2021JE007028 10.1029/2021JE007028

[CR9] Black GJ, Campbell DP, Nicholson PD (2001) Icy Galilean Satellites: Modeling Radar reflectivities as a coherent backscatter effect. Icarus 151(2):167–180. 10.1006/icar.2001.6616 10.1006/icar.2001.6616

[CR10] Bland MT, McKinnon WB (2015) Forming Ganymede’s grooves at smaller strain: toward a self-consistent local and global strain history for Ganymede. Icarus 245:247–262. 10.1016/j.icarus.2014.09.008 10.1016/j.icarus.2014.09.008

[CR11] Bland MT, Singer KN, McKinnon WB, Schenk PM (2017) Viscous relaxation of Ganymede’s impact craters: constraints on heat flux. Icarus 296:275–288. 10.1016/j.icarus.2017.06.012 10.1016/j.icarus.2017.06.012

[CR12] Blöcker A, Saur J, Roth L (2016) Europa’s plasma interaction with an inhomogeneous atmosphere: development of Alfvén winglets within the Alfvén wings. J Geophys Res Space Phys 121(10):9794–9828. 10.1002/2016JA02247 10.1002/2016JA02247

[CR13] Bockelée-Morvan D, Lellouch E, Poch O, Quirico E, Cazaux S, de Pater I, Fouchet T, Fry PM, Rodriguez-Ovalle P, Tosi F, Wong MH, Boshuizen I, de Kleer K, Fletcher LN, Mura A, Roth L, Saur J, Schmitt B, Trumbo SK, Brown ME, O’Donoghue J, Showalter MR (2024) Composition and thermal properties of Ganymede’s surface from JWST/NIRSpec and MIRI observations. Astron Astrophys 681:A27. 10.1051/0004-6361/202347326 10.1051/0004-6361/202347326

[CR14] Bottke WF, Vokrouhlický D, Nesvorný D, Moore JM (2013) Black rain: the burial of the Galilean satellites in irregular satellite debris. Icarus 223(2):775–795. 10.1016/j.icarus.2013.01.008 10.1016/j.icarus.2013.01.008

[CR15] Bottke WF, Vokrouhlický D, Nesvorný D, Marschall R, Morbidelli A, Deienno R, Marchi S, Kirchoff M, Dones L, Levison HF (2024) The bombardment history of the giant planet satellites. Planet Sci J 5(4):88. 10.3847/PSJ/ad29f4 10.3847/PSJ/ad29f4

[CR16] Bourgoin A, Gramigna E, Zannoni M, Gomez Casajus L, Tortora P (2022) Determination of uncertainty profiles in neutral atmospheric properties measured by radio occultation experiments. Adv Space Res 70(8):2555–2570. 10.1016/j.asr.2022.07.015 10.1016/j.asr.2022.07.015

[CR17] Boutonnet A, Langevin Y, Erd C (2024) Designing the JUICE Trajectory. Space Sci Rev 220

[CR18] Bray VJ, Schenk PM, Melosh HJ, Morgan JV, Collins GS (2012) Ganymede crater dimensions – implications for central peak and central pit formation and development. Icarus 217(1):115–129. 10.1016/j.icarus.2011.10.004 10.1016/j.icarus.2011.10.004

[CR19] Brown ME (2001) Potassium in Europa’s atmosphere. Icarus 151(2):190–195. 10.1006/icar.2001.6612 10.1006/icar.2001.6612

[CR20] Brown RH, Cruikshank DP (1997) Determination of the composition and state of icy surfaces in the outer Solar System. Annu Rev Earth Planet Sci 25:243–277. 10.1146/annurev.earth.25.1.243 10.1146/annurev.earth.25.1.243

[CR21] Brown ME, Hill RE (1996) Discovery of an extended sodium atmosphere around Europa. Nature 380(6571):229–231. 10.1038/380229a0 10.1038/380229a0

[CR22] Brown WL, Lanzerotti LJ, Poate JM, Augustyniak WM (1978) Sputtering of ice by MeV ions. Phys Rev Lett 49:1027–1030. 10.1103/PhysRevLett.40.1027 10.1103/PhysRevLett.40.1027

[CR23] Brown WL, Lanzerotti LJ, Johnson RE (1982) Fast ion bombardment of ices and its astrophysical implications. Science 218(4572):525–531. 10.1126/science.218.4572.525 17842043 10.1126/science.218.4572.525

[CR24] Brown RH, Cruikshank DP, Tokunaga AT, Smith RG, Clark RN (1988) Search for volatiles on icy satellites: I. Europa. Icarus 74(2):262–271. 10.1016/0019-1035(88)90041-3 10.1016/0019-1035(88)90041-3

[CR25] Brown RH, Clark RN, Buratti BJ, Cruikshank DP, Barnes JW, Mastrapa RME, Bauer J, Newman S, Momary T, Baines KH, Bellucci G, Capaccioni F, Cerroni P, Combes M, Coradini A, Drossart P, Formisano V, Jaumann R, Langevin Y, Matson DL, McCord TB, Nelson RM, Nicholson PD, Sicardy B, Sotin C (2006) Composition and physical properties of Enceladus’ surface. Science 311(5766):1425–1428. 10.1126/science.1121031 16527972 10.1126/science.1121031

[CR26] Bruzzone L, Plaut JJ et al (2024) RIME: Radar for Icy Moon Exploration. Space Sci Rev 220

[CR27] Burchell MJ (2013) Cratering on icy bodies. In: Gudipati MS, Castillo-Rogez J (eds) The science of Solar System ices. Springer, New York, pp 253–278. 10.1007/978-1-4614-3076-6_9

[CR28] Calvin WM, Clark RN (1991) Modeling the reflectance spectrum of Callisto 0.25 to 4.1 μm. Icarus 89(2):305–317. 10.1016/0019-1035(91)90180-2 10.1016/0019-1035(91)90180-2

[CR29] Cameron ME, Smith-Konter BR, Burkhard L, Collins GC, Seifert F, Pappalardo RT (2018) Morphological mapping of Ganymede: investigating the role of strike-slip tectonics in the evolution of terrain types. Icarus 315:92–114. 10.1016/j.icarus.2018.06.024 10.1016/j.icarus.2018.06.024

[CR30] Cameron ME, Smith-Konter BR, Collins GC, Patthoff DA, Pappalardo RT (2019) Tidal stress modeling of Ganymede: strike-slip tectonism and Coulomb failure. Icarus 319:99–120. 10.1016/j.icarus.2018.09.002 10.1016/j.icarus.2018.09.002

[CR31] Carberry Mogan SR, Tucker OJ, Johnson RE, Vorburger A, Galli A, Marchand B, Tafuni A, Kumar S, Sahin I, Sreenivasan KR (2021) A tenuous, collisional atmosphere on Callisto. Icarus 368:114597. 10.1016/j.icarus.2021.114597 10.1016/j.icarus.2021.114597

[CR32] Carberry Mogan SR, Tucker OJ, Johnson RE, Roth L, Alday J, Vorburger A, Wurz P, Galli A, Smith HT, Marchand B, Oza AV (2022) Callisto’s atmosphere: first evidence for H_2_ and constraints on H_2_O. J Geophys Res, Planets 127(11):e2022JE007294. 10.1029/2022JE007294 10.1029/2022JE007294

[CR33] Carlson RW (1999) A tenuous carbon dioxide atmosphere on Jupiter’s moon Callisto. Science 283(5403):820–821. 10.1126/science.283.5403.820 9933159 10.1126/science.283.5403.820

[CR34] Carlson RW, Bhattacharyya JC, Smith BA, Johnson TV, Hidayat B, Smith SA, Taylor GE, O’Leary B, Brinkmann RT (1973) An atmosphere on Ganymede from its occultation of SAO 186800 on 7 June 1972. Science 182(4107):53–55. 10.1126/science.182.4107.53 17829812 10.1126/science.182.4107.53

[CR35] Carlson RW, Weissman PR, Smythe WD, Mahoney JC (1992) Near-Infrared Mapping Spectrometer experiment on Galileo. Space Sci Rev 60(1–4):457–502. 10.1007/BF00216865 10.1007/BF00216865

[CR36] Carlson RW, Anderson MS, Johnson RE, Smythe WD, Hendrix AR, Barth CA, Soderblom LA, Hansen GB, McCord TB, Dalton JB, Clark RN, Shirley JH, Ocampo AC, Matson DL (1999a) Hydrogen peroxide on the surface of Europa. Science 283(5410):2062–2064. 10.1126/science.283.5410.2062 10092224 10.1126/science.283.5410.2062

[CR37] Carlson RW, Johnson RE, Anderson MS (1999b) Sulfuric acid on Europa and the radiolytic sulfur cycle. Science 286(5437):97–99. 10.1126/science.286.5437.97 10506568 10.1126/science.286.5437.97

[CR38] Carlson RW, Calvin WM, Dalton JB, Hansen GB, Hudson RL, Johnson RE, McCord TB, Moore MH (2009) Europa’s surface composition. In: Pappalardo RT, McKinnon WB, Khurana KK (eds) Europa. University of Arizona Press, Tucson, pp 283–327

[CR39] Carnielli G, Galand M, Leblanc F, Modolo R, Beth A, Jia X (2020) Constraining Ganymede’s neutral and plasma environments through simulations of its ionosphere and Galileo observations. Icarus 343:113691. 10.1016/j.icarus.2020.113691 10.1016/j.icarus.2020.113691

[CR40] Carr MH, Belton MJS, Chapman CR, Davies ME, Geissler P, Greenberg R, McEwen AS, Tufts BR, Greeley R, Sullivan R, Head JW, Pappalardo RT, Klaasen KP, Johnson TV, Kaufman J, Senske D, Moore J, Neukum G, Schubert G, Burns JA, Thomas P, Veverka J (1998) Evidence for a subsurface ocean on Europa. Nature 391(6665):363–365. 10.1038/34857 9450749 10.1038/34857

[CR41] Cartwright RJ, Villanueva GL, Holler BJ, Camarca M, Faggi S, Neveu M, Roth L, Raut U, Glein CR, Castillo-Rogez JC, Malaska MJ, Bockelée-Morvan D, Nordheim TA, Hand KP, Strazzulla G, Pendleton YJ, de Kleer K, Beddingfield CB, de Pater I, Cruikshank DP, Protopapa S (2024) Revealing Callisto’s carbon-rich surface and CO2 atmosphere with JWST. Planet Sci J 5(3):60. 10.3847/PSJ/ad23e6 10.3847/PSJ/ad23e6

[CR42] Cassidy TA, Paranicas CP, Shirley JH, Dalton JB III, Teolis BD, Johnson RE, Kamp L, Hendrix RA (2013) Magnetospheric ion sputtering and water ice grain size at Europa. Planet Space Sci 77:64–73. 10.1016/j.pss.2012.07.008 10.1016/j.pss.2012.07.008

[CR43] Choukroun M, Grasset O (2010) Thermodynamic data and modeling of the water and ammonia-water phase diagrams up to 2.2 GPa for planetary geophysics. J Chem Phys 133(14):144502. 10.1063/1.3487520 20950012 10.1063/1.3487520

[CR44] Chuang FC, Greeley R (2000) Large mass movements on Callisto. J Geophys Res 105(E8):20227–20244. 10.1029/2000JE001249 10.1029/2000JE001249

[CR45] Collins GC, Johnson TV (2014) Ganymede and Callisto. In: Spohn T, Breuer D, Johnson TV (eds) Encyclopedia of the Solar System, 3rd edn. Elsevier, Amsterdam, pp 813–829. 10.1016/B978-0-12-415845-0.00037-2

[CR46] Collins GC, Nimmo F (2009) Chaotic terrain on Europa. In: Pappalardo RT, McKinnon WB, Khurana KK (eds) Europa. University of Arizona Press, Tucson, pp 259–282

[CR47] Collins GC, Head JW, Pappalardo RT (1998) Formation of Ganymede Grooved Terrain by sequential extensional episodes: implications of Galileo observations for regional stratigraphy. Icarus 135(1):345–359. 10.1006/icar.1998.5978 10.1006/icar.1998.5978

[CR48] Collins GC, McKinnon WB, Moore JM, Nimmo F, McGill GE, Pappalardo RT, Schultz RA, Watters TR (2010) Tectonics of the outer planet satellites. In: Watters TR, Schultz RA (eds) Planetary tectonics. Cambridge University Press, Cambridge, pp 264–350. 10.1017/CBO9780511691645.008

[CR49] Collins GC, Patterson GW, Head JW, Pappalardo RT, Prockter LM, Lucchitta BK, Kay JP (2013) Global geological map of Ganymede: U.S. Geological Survey Scientific Investigations Map 3237, pamphlet 4 p., 1 sheet, scale 1:15,000,000. 10.3133/sim3237

[CR50] Collins GC, Patterson GW, Detelich CE, Prockter LM, Kattenhorn SA, Cooper CM, Rhoden AR, Cutler BB, Oldrid SR, Perkins RP, Rezza CA (2022) Episodic plate tectonics on Europa: evidence for widespread patches of mobile-lid behavior in the antijovian hemisphere. J Geophys Res, Planets 127(11):e2022JE007492. 10.1029/2022JE007492 10.1029/2022JE007492 PMC10078521 37035521

[CR51] Cooper JF, Johnson RE, Mauk MH, Garrett HB, Gerhels N (2001) Energetic ion and electron irradiation of the icy Galilean satellites. Icarus 149(1):133–159. 10.1006/icar.2000.6498 10.1006/icar.2000.6498

[CR52] Cruz Mermy G, Schmidt F, Andrieu F, Cornet T, Belgacem I, Altobelli N (2023) Selection of chemical species for Europa’s surface using Galileo/NIMS. Icarus 394:115379. 10.1016/j.icarus.2022.115379 10.1016/j.icarus.2022.115379

[CR53] Cunningham NJ, Spencer JR, Feldman PD, Strobel DF, France K, Osterman SN (2015) Detection of Callisto’s oxygen atmosphere with the Hubble Space Telescope. Icarus 254:178–189. 10.1016/j.icarus.2015.03.021 10.1016/j.icarus.2015.03.021

[CR54] Dalton JB (2003) Spectral behavior of hydrated sulfate salts: implications for Europa mission spectrometer design. Astrobiology 3(4):771–784. 10.1089/153110703322736097 14987482 10.1089/153110703322736097

[CR55] Dalton JB (2007) Linear mixture modeling of Europa’s non-ice material based on cryogenic laboratory spectroscopy. Geophys Res Lett 34(21):L21205. 10.1029/2007GL031497 10.1029/2007GL031497

[CR56] Dalton JB, Prieto-Ballesteros O, Kargel JS, Jamieson CS, Jolivet J, Quinn R (2005) Spectral comparison of heavily hydrated salts with disrupted terrains on Europa. Icarus 177(2):472–490. 10.1016/j.icarus.2005.02.023 10.1016/j.icarus.2005.02.023

[CR57] Dalton JB, Shirley JH, Kamp LW (2012) Europa’s icy bright plains and dark linea: exogenic and endogenic contributions to composition and surface properties. J Geophys Res, Planets 117(E3):E03003. 10.1029/2011JE003909 10.1029/2011JE003909

[CR58] Dalton JB, Cassidy T, Paranicas C, Shirley JH, Prockter LM, Kamp LW (2013) Exogenic controls on sulfuric acid hydrate production at the surface of Europa. Planet Space Sci 77:45–63. 10.1016/j.pss.2012.05.013 10.1016/j.pss.2012.05.013

[CR59] Dayton-Oxland R, Huybrighs HLF, Winterhalder TO, Mahieux A, Goldstein D (2023) In-situ detection of Europa’s water plumes is harder than previously thought. Icarus 395:115488. 10.1016/j.icarus.2023.115488 10.1016/j.icarus.2023.115488

[CR60] De Angelis S, Carli C, Tosi F, Beck P, Schmitt B, Piccioni G, De Sanctis MC, Capaccioni F, Di Iorio T, Philippe S (2017) Temperature-dependent VNIR spectroscopy of hydrated Mg-sulfates. Icarus 281:444–458. 10.1016/j.icarus.2016.07.022 10.1016/j.icarus.2016.07.022

[CR61] De Angelis S, Carli C, Tosi F, Beck P, Brissaud O, Schmitt B, Potin S, De Sanctis MC, Capaccioni F, Piccioni G (2019) NIR reflectance spectroscopy of hydrated and anhydrous sodium carbonates at different temperatures. Icarus 317:388–411. 10.1016/j.icarus.2018.08.012 10.1016/j.icarus.2018.08.012

[CR62] De Angelis S, Tosi F, Carli C, Potin S, Beck P, Brissaud O, Schmitt B, Piccioni G, De Sanctis MC, Capaccioni F (2021) Temperature-dependent, VIS-NIR reflectance spectroscopy of sodium sulfates. Icarus 357:114165. 10.1016/j.icarus.2020.114165 10.1016/j.icarus.2020.114165

[CR63] De Angelis S, Tosi F, Carli C, Beck P, Brissaud O, Schmitt B (2022) VIS-IR spectroscopy of magnesium chlorides at cryogenic temperatures. Icarus 373:114756. 10.1016/j.icarus.2021.114756 10.1016/j.icarus.2021.114756

[CR64] de Kleer K, Milby Z, Schmidt C, Camarca M, Brown ME (2023) The optical aurorae of Europa, Ganymede, and Callisto. Planet Sci J 4(2):37. 10.3847/PSJ/acb53c 10.3847/PSJ/acb53c

[CR65] Denk T, Williams DA, Tosi F, Bell JF III, Mottola S, de Pater I, Lainey V, Molyneux P, Matz K-D, Hartogh P, Lopes RM, Solomonidou A, Thomas PC, Huybrighs HLF, Gurvits LI, Mura A, Retherford KD, Rezac L, Roatsch T, Roth L, Haslebacher N, Tubiana C, Langevin Y, Lellouch E, Lucchettii A, Poulet P, Tsuchiya F, Vallat C, van Hoolst T, Vorburger A, Wurz P, D’Aversa E, Gladstone R, Greathouse T, Schneider N, Zambon F, Altobelli N, Palumbo P, Portyankina A, Aharonson O, Bruzzone L, Carter J, Cecconi B, Cooper N, Costa Sitja M, Escalante Lopez A, FutaanaY MA, Moore WB, Moreno R, Murray C, Penasa L, Piccioni G, Schmidt J, Wahlund J-E, Witasse O (2024) Io and the Minor Jovian Moons – Prospects for JUICE. Space Sci Rev 220

[CR66] Divine N (1993) Five populations of interplanetary meteoroids. J Geophys Res 98(E9):17029–17048. 10.1029/93JE01203 10.1029/93JE01203

[CR67] Dols VJ, Bagenal F, Cassidy TA, Crary FJ, Delamere PA (2016) Europa’s atmospheric neutral escape: importance of symmetrical O_2_ charge exchange. Icarus 264:387–397. 10.1016/j.icarus.2015.09.026 10.1016/j.icarus.2015.09.026

[CR68] Dombard AJ, McKinnon WB (2001) Formation of grooved terrain on Ganymede: extensional instability mediated by cold, superplastic creep. Icarus 154(2):321–336. 10.1006/icar.2001.6728 10.1006/icar.2001.6728

[CR69] Domingue DL, Lane AL, Beyer RA (1998) IUE’s detection of tenuous SO2 frost on Ganymede and its rapid time variability. Geophys Res Lett 25(16):3117–3120. 10.1029/98GL02386 10.1029/98GL02386

[CR70] Dones L, Chapman CR, McKinnon WB, Melosh HJ, Kirchoff MR, Neukum R, Zahnle KJ (2009) Icy satellites of Saturn: impact cratering and age determination. In: Dougherty MK, Esposito LW, Krimigis SM (eds) Saturn from Cassini-Huygens. Springer, Dordrecht, pp 613–635. 10.1007/978-1-4020-9217-6_19

[CR71] Dougherty MK, Khurana KK, Neubauer FM, Russell CT, Saur J, Leisner JS, Burton ME (2006) Identification of a dynamic atmosphere at Enceladus with the Cassini magnetometer. Science 311(5766):1406–1409. 10.1126/science.1120985 16527966 10.1126/science.1120985

[CR72] Douté S, Schmitt B, Lopes-Gautier R, Carlson RW, Soderblom L, Shirley J (the Galileo NIMS Team) (2001) Mapping SO2 frost on Io by the modeling of NIMS hyperspectral images. Icarus 149(1):107–132. 10.1006/icar.2000.6513 10.1006/icar.2000.6513

[CR73] Fagents SA (2003) Considerations for effusive cryovolcanism on Europa: the post-Galileo perspective. J Geophys Res 108(E12):5139. 10.1029/2003JE002128 10.1029/2003JE002128

[CR74] Fagents SA, Greeley R, Sullivan RJ, Pappalardo RT, Prockter LM (Galileo SSI Team) (2000) Cryomagmatic mechanisms for the formation of Rhadamanthys Linea, triple band margins, and other low-albedo features on Europa. Icarus 144(1):54–88. 10.1006/icar.1999.6254 10.1006/icar.1999.6254

[CR75] Fagents SA, Lopes RMC, Quick LC, Gregg TKP (2022) Cryovolcanism. In: Gregg TKP, Lopes RMC, Fagents SA (eds) Planetary volcanism across the Solar System. Elsevier, Amsterdam, pp 161–234. 10.1016/B978-0-12-813987-5.00005-5

[CR76] Famá M, Shi J, Baragiola RA (2008) Sputtering of ice by low-energy ions. Surf Sci 602(1):156–161. 10.1016/j.susc.2007.10.002 10.1016/j.susc.2007.10.002

[CR77] Fatemi S, Poppe AR, Khurana KK, Holmström M, Delory GT (2016) On the formation of Ganymede’s surface brightness asymmetries: kinetic simulations of Ganymede’s magnetosphere. Geophys Res Lett 43(10):4745–4754. 10.1029/2019JA026643 10.1029/2019JA026643

[CR78] Feldman PD, McGrath MA, Strobel DF, Moos HW, Retherford KD, Wolven BC (2000) HST/STIS Ultraviolet imaging of polar aurora on Ganymede. Astrophys J 535(2):1085–1090. 10.1086/308889 10.1086/308889

[CR79] Feldman PD, Glenar DA, Stubbs TJ, Retherford KD, Gladstone GR, Miles PF, Greathouse TK, Kaufmann DE, Parker JW, Stern SA (2014) Upper limits for a lunar dust exosphere from far-ultraviolet spectroscopy by LRO/LAMP. Icarus 233:106–133. 10.1016/j.icarus.2014.01.039 10.1016/j.icarus.2014.01.039

[CR80] Gaidos E, Nimmo F (2000) Tectonics and water on Europa. Nature 405(6787):637. 10.1038/35015170 10864313 10.1038/35015170

[CR81] Galli A, Vorburger A, Wurz P, Pommerol A, Cerubini R, Jost B, Poch O, Tulej M, Thomas N (2018) 0.2 to 10 keV electrons interacting with water ice: radiolysis, sputtering, and sublimation. Planet Space Sci 155:91–98. 10.1016/j.pss.2017.11.016 10.1016/j.pss.2017.11.016

[CR82] Galli A, Vorburger A, Carberry Mogan SR, Roussos E, Stenberg Wieser G, Wurz P, Föhn M, Krupp N, Fränz M, Barabash S, Futaana Y, Brandt PC, Kollmann P, Haggerty DK, Jones GH, Johnson RE, Tucker OJ, Simon S, Tippens T, Liuzzo L (2022) Callisto’s atmosphere and its space environment: prospects for the particle environment package on board JUICE. Earth Space Sci 9(5):e2021EA002172. 10.1029/2021EA002172 10.1029/2021EA002172

[CR83] Galli A, Vorburger A, Wurz P, Galand M, Oza A, Fatemi S, Plainaki C, Mura A (2024) Interactions between the space environment and Ganymede’s surface. In: Volwerk M, McGrath M, Jia X, Spohn T (eds) Ganymede. Planetary science series, vol 28. Cambridge University Press, Cambridge. https://wurz.space.unibe.ch/Ganymede_book_3p2_rev.pdf

[CR84] Geissler P (2015) Cryovolcanism in the outer Solar System. In: Sigurdsson H (ed) The encyclopedia of volcanoes, 2nd edn. Elsevier, Amsterdam, pp 763–776. 10.1016/B978-0-12-385938-9.00044-4

[CR85] Giese B, Oberst J, Roatsch T, Neukum G, Head JW, Pappalardo RT (1998) The local topography of Uruk Sulcus and Galileo Regio obtained from stereo images. Icarus 135(1):303–316. 10.1006/icar.1998.5967 10.1006/icar.1998.5967

[CR86] Giese B, Wagner R, Hussmann H, Neukum G, Perry J, Helfenstein P, Thomas PC (2008) Enceladus: an estimate of heat flux and lithospheric thickness from flexurally supported topography. Geophys Res Lett 35(24):L24204. 10.1029/2008GL036149 10.1029/2008GL036149

[CR87] Giese B, Hauber E, Hussmann H (2017) On the formation of caldera-like features on Ganymede: implications from Galileo-G28 images. 48th Lunar and Planetary Science Conference, LPI Contribution No. 1964, #2474

[CR88] Giono G, Roth L, Ivchenko N, Saur J, Retherford K, Schlegel S, Ackland M, Strobel D (2020) An analysis of the statistics and systematics of limb anomaly detections in HST/STIS transit images of Europa. Astron J 159(4):155. 10.3847/1538-3881/ab7454 10.3847/1538-3881/ab7454

[CR89] Golombek MP, Allison ML (1981) Sequential development of grooved terrain and polygons on Ganymede. Geophys Res Lett 8(11):1139–1142. 10.1029/GL008i011p01139 10.1029/GL008i011p01139

[CR90] Grasset O, Dougherty MK, Coustenis A, Bunce EJ, Erd C, Titov D, Blanc M, Coates A, Drossart P, Fletcher LN, Hussmann H, Jaumann R, Krupp N, Lebreton JP, Prieto-Ballesteros O, Tortora P, Tosi F, Van Hoolst T (2013) JUpiter ICy moons Explorer (JUICE): an ESA mission to orbit Ganymede and to characterise the Jupiter system. Planet Space Sci 78:1–21. 10.1016/j.pss.2012.12.002 10.1016/j.pss.2012.12.002

[CR91] Greathouse TK, Gladstone GR, Molyneux PM, Versteeg MH, Hue V, Kammer JA, Davis MW, Bolton SJ, Giles RS, Connerney JEP, Gerard J-C, Grodent DC, Bonfond B, Saur J, Duling S (2022) UVS observations of Ganymede’s aurora during Juno orbits 34 and 35. Geophys Res Lett 49(23):e2022GL099794. 10.1029/2022GL099794 10.1029/2022GL099794

[CR92] Greeley R, Klemaszewski JE, Wagner R (2000) Galileo views of the geology of Callisto. Planet Space Sci 48:829–853. 10.1016/S0032-0633(00)00050-7 10.1016/S0032-0633(00)00050-7

[CR93] Greeley R, Pappalardo RT, Prockter LM, Hendrix AR, Lock RE (2009) Future exploration of Europa. In: Pappalardo RT, McKinnon WB, Khurana KK (eds) Europa. University of Arizona Press, Tucson, pp 655–696

[CR94] Greenberg R, Hoppa GV, Tufts BR, Geissler P, Riley J, Kadel S (1999) Chaos on Europa. Icarus 141(2):263–286. 10.1006/icar.1999.6187 10.1006/icar.1999.6187

[CR95] Grundy WM, Schmitt B (1998) The temperature-dependent near-infrared absorption spectrum of hexagonal H2O ice. J Geophys Res, Planets 103(E11):25809–25822. 10.1029/98JE00738 10.1029/98JE00738

[CR96] Haffoud P, Poulet F, Vincendon M, Filacchione G, Barbis A, Guiot P, Lecomte B, Langevin Y, Piccioni G, Dumesnil C, Rodriguez S, Carter J, Stefani S, Tommasi L, Tosi F, Pilorget C (2024) Calibration of MAJIS (Moons And Jupiter Imaging Spectrometer). III. Spectral calibration. Rev Sci Instrum 95(3):031301. 10.1063/5.0188944 38451143 10.1063/5.0188944

[CR97] Hall DT, Strobel DF, Feldman PD, McGrath MA, Weaver HA (1995) Detection of an oxygen atmosphere on Jupiter’s moon Europa. Nature 373(6516):677–679. 10.1038/373677a0 7854447 10.1038/373677a0

[CR98] Hall DT, Feldman PD, McGrath MA, Strobel DF (1998) The far-ultraviolet oxygen airglow of Europa and Ganymede. Astrophys J 499(5):475–481. 10.1086/305604 10.1086/305604

[CR99] Hanel R, Pearl JC, Lowman P, Kumar S, Horn L (1979) Preliminary results from Voyager 1 infrared observations of the Jovian satellites. EOS Trans 60

[CR100] Hansen GB, McCord TB (2004) Amorphous and crystalline ice on the Galilean satellites: a balance between thermal and radiolytic processes. J Geophys Res, Planets 109(E1):E01012. 10.1029/2003JE002149 10.1029/2003JE002149

[CR101] Hansen GB, McCord TB (2008) Widespread CO_2_ and other non-ice compounds on the anti-Jovian and trailing sides of Europa from Galileo/NIMS observations. Geophys Res Lett 35(1):L01202. 10.1029/2007GL031748 10.1029/2007GL031748

[CR102] Hansen CJ, Shemansky DE, Hendrix AR (2005) Cassini UVIS observations of Europa’s oxygen atmosphere and torus. Icarus 176(2):305–315. 10.1016/j.icarus.2005.02.007 10.1016/j.icarus.2005.02.007

[CR103] Hansen CJ, Esposito LW, Hendrix AR (2019) Ultraviolet observation of Enceladus’ plume in transit across Saturn, compared to Europa. Icarus 330:256–260. 10.1016/j.icarus.2019.04.031 10.1016/j.icarus.2019.04.031

[CR104] Hansen CJ, Esposito LW, Colwell JE, Hendrix AR, Portyankina G, Stewart AIF, West RA (2020) The composition and structure of Enceladus’ plume from the complete set of Cassini UVIS occultation observations. Icarus 344:113461. 10.1016/j.icarus.2019.113461 10.1016/j.icarus.2019.113461

[CR105] Hansen CJ, Bolton S, Sulaiman AH, Duling S, Bagenal F, Brennan M, Connerney J, Clark G, Lunine J, Levin S, Kurth W, Mura M, Paranicas C, Tosi F, Withers P (2022) Juno’s close encounter with Ganymede – an overview. Geophys Res Lett 49(23):e2022GL099285. 10.1029/2022GL099285 10.1029/2022GL099285 PMC10078441 37034391

[CR106] Hansen CJ, Ravine MA, Schenk PM, Collins GC, Leonard EJ, Phillips CB, Caplinger MA, Tosi F, Bolton SJ, Jónsson B (2024) Juno’s JunoCam images of Europa. Planet Sci J 5(3):76. 10.3847/PSJ/ad24f4 10.3847/PSJ/ad24f4

[CR107] Hapke B (1990) Coherent backscatter and the radar characteristics of outer planet satellites. Icarus 88(2):407–417. 10.1016/0019-1035(90)90091-M 10.1016/0019-1035(90)90091-M

[CR108] Hartkorn O, Saur J, Strobel DF (2017) Structure and density of Callisto’s atmosphere from a fluid-kinetic model of its ionosphere: comparison with Hubble Space Telescope and Galileo observations. Icarus 282:237–259. 10.1016/j.icarus.2016.09.020 10.1016/j.icarus.2016.09.020

[CR109] Hartogh P, Bockelée-Morvan D, Rezac L, Moreno R, Lellouch E, Rengel M, Jarchow C, de Val-Borro M, Crovisier J, Biver N (2013) Detection and characterization of Ganymede’s and Callisto’s water atmospheres. In: International symposium “the universe explored by Herschel”, ESTEC, Netherlands, 15–18 October 2013. http://herschel.esac.esa.int/TheUniverseExploredByHerschel/presentations/13a-1720_HartoghP.pdf

[CR110] Hartogh P et al (2024) The sub-millimeter wave Instrument (SWI) for the JUICE mission. Space Sci Rev 220

[CR111] Hauber E, Grott M, Kronberg P (2010) Martian rifts: structural geology and geophysics. Earth Planet Sci Lett 294(3–4):393–410. 10.1016/j.epsl.2009.11.005 10.1016/j.epsl.2009.11.005

[CR112] Hedman MM, Nicholson PD, Showalter MR, Brown RH, Buratti BJ, Clark RN (2009) Spectral observations of the Enceladus plume with Cassini-VIMS. Astrophys J 693(2):1749. 10.1088/0004-637X/693/2/1749 10.1088/0004-637X/693/2/1749

[CR113] Heggy E, Scabbia G, Bruzzone L, Pappalardo RT (2017) Radar probing of Jovian icy moons: understanding subsurface water and structure detectability in the JUICE and Europa missions. Icarus 285:237–251. 10.1016/j.icarus.2016.11.039 10.1016/j.icarus.2016.11.039

[CR114] Hendrix AR, Johnson RE (2008) Callisto: new insights from Galileo disk-resolved UV measurements. Astrophys J 687(1):706. 10.1086/591491 10.1086/591491

[CR115] Hendrix AR, Barth CA, Hord CW, Lane AL (1998) Europa: disk-resolved ultraviolet measurements using the Galileo ultraviolet spectrometer. Icarus 135(1):79–84. 10.1006/icar.1998.5983 10.1006/icar.1998.5983

[CR116] Hendrix AR, Barth CA, Hord CW (1999) Ganymede’s ozone-like absorber: observations by the Galileo ultraviolet spectrometer. J Geophys Res 104(E6):14169–14178. 10.1029/1999JE900001 10.1029/1999JE900001

[CR117] Hendrix AR, Cassidy TA, Johnson RE, Paranicas C, Carlson RW (2011) Europa’s disk-resolved ultraviolet spectra: relationships with plasma flux and surface terrains. Icarus 212(2):736–743. 10.1016/j.icarus.2011.01.023 10.1016/j.icarus.2011.01.023

[CR118] Hibbitts CA, Hansen GB (2001) The non-ice material on Callisto. EOS 82, abstract n. #P12B-0495

[CR119] Hibbitts CA, McCord TB, Hansen GB (2000) Distributions of CO_2_ and SO_2_ on the surface of Callisto. J Geophys Res 105(E9):22541–22558. 10.1029/1999JE001101 10.1029/1999JE001101

[CR120] Hibbitts CA, Klemaszewski JE, McCord TB, Hansen GB, Greeley R (2002) CO_2_-rich impact craters on Callisto. J Geophys Res 107(E10):5084–5100. 10.1029/2000JE001412 10.1029/2000JE001412

[CR121] Hibbitts CA, Pappalardo RT, Hansen GB, McCord TB (2003) Carbon dioxide on Ganymede. J Geophys Res 108(E5):5036. 10.1029/2002JE001956 10.1029/2002JE001956

[CR122] Hibbitts CA, Stephan K, Collins G, Hansen GB (2009) Composition and distribution of nonice and trace materials on Ganymede as derived from Galileo observations. EPSC 2009, abstract n. 632. https://meetingorganizer.copernicus.org/epsc2009/epsc2009-632.pdf

[CR123] Hoppa GV, Tufts BR, Greenberg R, Geissler P (1999a) Strike-slip faults on Europa: global shear patterns driven by tidal stress. Icarus 141(2):287–298. 10.1006/icar.1999.6185 10.1006/icar.1999.6185

[CR124] Hoppa GV, Tufts BR, Greenberg R, Geissler PE (1999b) Formation of cycloidal features on Europa. Science 285(5435):1899–1902. 10.1126/science.285.5435.1899 10489365 10.1126/science.285.5435.1899

[CR125] Hord CW, McClintock WE, Stewart AIF, Barth CA, Esposito LW, Thomas GE, Sandel BR, Hunten DM, Broadfoot AL, Shemansky DE (1992) Galileo ultraviolet spectrometer experiment. Space Sci Rev 60(1–4):503–530. 10.1007/BF00216866 10.1007/BF00216866 17784100

[CR126] Hurford TA, Beyer RA, Schmidt B, Preblich B, Sarid AR, Greenberg R (2005) Flexure of Europa’s lithosphere due to ridge-loading. Icarus 177(2):380–396. 10.1016/j.icarus.2005.06.019 10.1016/j.icarus.2005.06.019

[CR127] Hussmann H et al (2024) The Ganymede Laser Altimeter (GALA) on the Jupiter Icy Moons Explorer (JUICE) Mission: Scientific Objectives and Experiment Description. Space Sci Rev 220

[CR128] Huybrighs HLF, Futaana Y, Barabash S, Wieser M, Wurz P, Krupp N, Glassmeier K-H, Vermeersen B (2017) On the in-situ detectability of Europa’s water vapour plumes from a flyby mission. Icarus 289:270–280. 10.1016/j.icarus.2016.10.026 10.1016/j.icarus.2016.10.026

[CR129] Huybrighs HLF, Roussos E, Blöcker A, Krupp N, Futaana Y, Barabash S, Hadid LZ, Holmberg MKG, Lomax O, Witasse O (2020) An active plume eruption on Europa during Galileo flyby E26 as indicated by energetic proton depletions. Geophys Res Lett 47(10):e2020GL087806. 10.1029/2020GL087806 10.1029/2020GL087806

[CR130] Huybrighs HLF, Roussos E, Blöcker A, Krupp N, Futaana Y, Barabash S, Hadid LZ, Holmberg MKG, Witasse O (2021) Reply to comment on “An active plume eruption on Europa during Galileo flyby E26 as indicated by energetic proton depletions”. Geophys Res Lett 48(18):e2021GL095240. 10.1029/2021GL095240 10.1029/2021GL095240

[CR131] Ilyushin YA, Hartogh P (2020) Submillimeter Wave Instrument radiometry of the Jovian icy moons. Numerical simulation of the microwave thermal radiative transfer and Bayesian retrieval of the physical properties. Astron Astrophys 644:A24. 10.1051/0004-6361/201937220 10.1051/0004-6361/201937220

[CR132] Ip W-H (1996) Europa’s oxygen exosphere and its magnetospheric interaction. Icarus 120(2):317–325. 10.1006/icar.1996.0052 10.1006/icar.1996.0052

[CR133] Jaumann R, Stephan K, Hansen GB, Clark RN, Buratti BJ, Brown RH, Baines KH, Newman SF, Bellucci G, Filacchione G, Coradini A, Cruikshank DP, Griffith CA, Hibbitts CA, McCord TB, Nelson RM, Nicholson PD, Sotin C, Wagner R (2008) Distribution of icy particles across Enceladus’ surface as derived from Cassini-VIMS measurements. Icarus 193(2):407–419. 10.1016/j.icarus.2007.09.013 10.1016/j.icarus.2007.09.013

[CR134] Jia X, Kivelson MG, Khurana KK, Kurth WS (2018) Evidence of a plume on Europa from Galileo magnetic and plasma wave signatures. Nat Astron 2(6):459–464. 10.1038/s41550-018-0450-z 10.1038/s41550-018-0450-z

[CR135] Jia X, Kivelson MG, Paranicas C (2021) Comment on “An active plume eruption on Europa during Galileo flyby E26 as indicated by energetic proton depletions” by Huybrighs et al. Geophys Res Lett 48(6):e2020GL091550. 10.1029/2020GL091550 10.1029/2020GL091550

[CR136] Johnson RE (1997) Polar “caps” on Ganymede and Io revisited. Icarus 128(2):469–471. 10.1006/icar.1997.5746 10.1006/icar.1997.5746

[CR137] Johnson TV (2005) Geology of the icy satellites. Space Sci Rev 116:401–420. 10.1007/s11214-005-1963-1 10.1007/s11214-005-1963-1

[CR138] Johnson RE, Lanzerotti LJ, Brown WL, Armstrong TP (1981) Erosion of Galilean satellite surfaces by Jovian magnetosphere particles. Science 212(4498):1027–1030. 10.1126/science.212.4498.1027 17779973 10.1126/science.212.4498.1027

[CR139] Johnson RE, Burger MH, Cassidy TA, Leblanc F, Marconi M, Smyth WH, Dotson R (2009) Composition and detection of Europa’s sputter-induced atmosphere. In: Pappalardo RT, McKinnon WB, Khurana KK (eds) Europa. University of Arizona Press, Tucson, pp 507–528. 10.2307/j.ctt1xp3wdw.27

[CR140] Journaux B, Kalousová K, Sotin C, Tobie G, Vance S, Saur J, Bollengier O, Noack L, Rückriemen-Bez T, Van Hoolst T, Soderlund KM, Brown JM (2020) Large ocean worlds with high-pressure ices. Space Sci Rev 216(1):1–36. 10.1007/s11214-019-0633-7 10.1007/s11214-019-0633-7

[CR141] Journaux B, Pakhomova A, Collings IE, Petitgirard S, Boffa Ballaran T, Brown JM, Vance SD, Chariton S, Prakapenka VB, Huang D, Ott J, Glazyrin K, Garbarino G, Combonic D, Hanfland M (2023) On the identification of hyperhydrated sodium chloride hydrates, stable at icy moon conditions. Proc Natl Acad Sci USA 120(9):e2217125120. 10.1073/pnas.2217125120 36802438 10.1073/pnas.2217125120 PMC9992769

[CR142] Kanik I, Johnson PV, Das MB, Khakoo MA, Tayal SS (2001) Electron-impact studies of atomic oxygen: I. Differential and integral cross sections; experiment and theory. J Phys B, At Mol Opt Phys 34:2647–2665. 10.1088/0953-4075/34/13/308 10.1088/0953-4075/34/13/308

[CR143] Kanik I, Noren C, Makarov OP, Vatti Palle P, Ajello JM, Shemansky DE (2003) Electron impact dissociative excitation of O_2_: 2. Absolute emission cross sections of the OI(130.4 nm) and OI(135.6 nm) lines. J Geophys Res, Planets 108(E11):5126. 10.1029/2000JE001423 10.1029/2000JE001423

[CR144] Kargel JS, Kaye JZ, Head JW, Marion GM, Sassen R, Crowley JK, Ballesteros OP, Grant SA, Hogenboom DL (2000) Europa’s crust and ocean: origin, composition, and the prospects for life. Icarus 148(1):226–265. 10.1006/icar.2000.6471 10.1006/icar.2000.6471

[CR145] Kattenhorn SA (2004) Strike-slip fault evolution on Europa: evidence from tailcrack geometries. Icarus 172(2):582–602. 10.1016/j.icarus.2004.07.005 10.1016/j.icarus.2004.07.005

[CR146] Kattenhorn SA, Hurford TA (2009) Tectonics of Europa. In: Pappalardo RT, McKinnon WB, Khurana KK (eds) Europa. University of Arizona Press, Tucson, pp 199–236

[CR147] Kay JE, Head JWI (1999) Geological mapping of the Ganymede G8 calderas region: evidence for cryovolcanism. In: Lunar and Planetary Science Conference, vol 30, p 1103

[CR148] Kersten E, Zubarev AE, Roatsch T, Matz KD (2021) Controlled global Ganymede mosaic from Voyager and Galileo images. Planet Space Sci 206:105310. 10.1016/j.pss.2021.105310 10.1016/j.pss.2021.105310

[CR149] Khurana KK, Kivelson MG, Stevenson DJ, Schubert G, Russell CT, Walker RJ, Polanskey C (1998) Induced magnetic fields as evidence for subsurface oceans in Europa and Callisto. Nature 395(6704):777–780. 10.1038/27394 9796812 10.1038/27394

[CR150] Khurana KK, Pappalardo RT, Murphy N, Denk T (2007) The origin of Ganymede’s polar caps. Icarus 191(1):193–202. 10.1016/j.icarus.2007.04.022 10.1016/j.icarus.2007.04.022

[CR151] King O, Fletcher LN (2022) Global modelling of Ganymede’s surface composition: near-IR mapping from VLT/SPHERE. J Geophys Res, Planets 127(12):e2022JE007323. 10.1029/2022JE007323 10.1029/2022JE007323

[CR152] King O, Fletcher LN, Ligier N (2022) Compositional mapping of Europa using MCMC modeling of near-IR VLT/SPHERE and Galileo/NIMS observations. Planet Sci J 3(3):72. 10.3847/PSJ/ac596d 10.3847/PSJ/ac596d

[CR153] Kirchoff MR, Barr A, Bland M, Bray V, Rivera-Valentín EG, Schenk P (2024) The cratering record of Ganymede. In: Volwerk M, McGrath M, Jia X, Spohn T (eds) Ganymede. Planetary science series, vol 28. Cambridge University Press, Cambridge, pp 104–125

[CR154] Kivelson M, Khurana K, Russell CT, Volwerk M, Walker RJ, Zimmer C (2000) Galileo magnetometer measurements: a stronger case for a subsurface ocean at Europa. Science 289(5483):1340–1343. 10.1126/science.289.5483.1340 10958778 10.1126/science.289.5483.1340

[CR155] Kivelson M, Khurana K, Volwerk M (2002) The permanent and inductive magnetic moments of Ganymede. Icarus 157(2):507–522. 10.1006/icar.2002.6834 10.1006/icar.2002.6834

[CR156] Kliore AJ, Anabtawi A, Herrera RG, Asmar SW, Nagy AF, Hinson DP, Flasar F (2002) Ionosphere of Callisto from Galileo radio occultation observations. J Geophys Res 107(A11):1407. 10.1029/2002JA009365 10.1029/2002JA009365

[CR157] Krivov AV, Sremčević M, Spahn F, Dikarev VV, Kholshevnikov KV (2003) Impact-generated dust clouds around planetary satellites: spherically symmetric case. Planet Space Sci 51(3):251–269. 10.1016/S0032-0633(02)00147-2 10.1016/S0032-0633(02)00147-2

[CR158] Krüger H, Krivov AV, Hamilton DP, Grün E (1999) Detection of an impact-generated dust cloud around Ganymede. Nature 399(6736):558–560. 10.1038/21136 10.1038/21136

[CR159] Krüger H, Krivov AV, Sremčević M, Grün E (2003) Impact-generated dust clouds surrounding the Galilean moons. Icarus 164(1):170–187. 10.1016/S0019-1035(03)00127-1 10.1016/S0019-1035(03)00127-1

[CR160] Kuiper GP (1957) Infrared observations of planets and satellites. Astrophys J 62:245

[CR161] Küppers M, O’Rourke L, Bockelée-Morvan D, Zakharov V, Lee S, von Allmen P, Carry B, Teyssier D, Marston A, Müller T, Crovisier J, Barucci MA, Moreno R (2014) Localized sources of water vapour on the dwarf planet (1) Ceres. Nature 505(7484):525–527. 10.1038/nature12918 24451541 10.1038/nature12918

[CR162] Kurth WS, Wilkinson DR, Hospodarsky GB, Santolík O, Averkamp TF, Sulaiman AH, Menietti JD, Connerney JEP, Allegrini F, Mauk BH, Bolton SJ (2023) Juno plasma wave observations at Europa. Geophys Res Lett 50(24):e2023GL105775. 10.1029/2023GL105775 10.1029/2023GL105775 PMC10078157 37034392

[CR163] Lanzerotti LJ, Brown WL, Poate JM, Augustyniak WM (1978) On the contribution of water products from Galilean satellites to the Jovian magnetosphere. Geophys Res Lett 5(2):155–158. 10.1029/GL005i002p00155 10.1029/GL005i002p00155

[CR164] Leblanc F, Oza AV, Leclercq L, Schmidt C, Cassidy T, Modolo R, Chaufray JY, Johnson RE (2017) On the orbital variability of Ganymede’s atmosphere. Icarus 293:185–198. 10.1016/j.icarus.2017.04.025 10.1016/j.icarus.2017.04.025

[CR165] Liang M-C, Lane BF, Pappalardo RT, Allen M, Yung YL (2005) Atmosphere of Callisto. J Geophys Res 110(E2):E02003. 10.1029/2004JE002322 10.1029/2004JE002322

[CR166] Ligier N, Poulet F, Carter J, Brunetto R, Gourgeot F (2016) VLT/SINFONI observations of Europa: new insights into the surface composition. Astron J 151(6):163. 10.3847/0004-6256/151/6/163 10.3847/0004-6256/151/6/163

[CR167] Ligier N, Paranicas C, Carter J, Poulet F, Calvin WM, Nordheim TA, Snodgrass C, Ferellec L (2019) Surface composition and properties of Ganymede: updates from ground-based observations with the near-infrared imaging spectrometer SINFONI/VLT/ESO. Icarus 333:496–515. 10.1016/j.icarus.2019.06.013 10.1016/j.icarus.2019.06.013

[CR168] Liuzzo L, Simon S, Regoli L (2019a) Energetic ion dynamics near Callisto. Planet Space Sci 166:23–53. 10.1016/j.pss.2018.07.014 10.1016/j.pss.2018.07.014

[CR169] Liuzzo L, Simon S, Regoli L (2019b) Energetic electron dynamics near Callisto. Planet Space Sci 179:104726. 10.1016/j.pss.2019.104726 10.1016/j.pss.2019.104726

[CR170] Liuzzo L, Poppe AR, Paranicas C, Nénon Q, Fatemi S, Simon S (2020) Variability in the energetic electron bombardment of Ganymede. J Geophys Res Space Phys 125(9):e28347. 10.1029/2020JA028347 10.1029/2020JA028347

[CR171] Lucas A (2012) Slippery sliding on icy Iapetus. Nat Geosci 5(8):524–525. 10.1038/ngeo1532 10.1038/ngeo1532

[CR172] Lucchetti A, Dalle Ore C, Pajola M, Pozzobon R, Rossi C, Galluzzi V, Penasa L, Stephan K, Munaretto G, Cremonese G, Massironi M, Palumbo P (2023) Geological, compositional and crystallinity analysis of the Melkart impact crater, Ganymede. Icarus 401:115613. 10.1016/j.icarus.2023.115613 10.1016/j.icarus.2023.115613

[CR173] Lucchitta BK (1980) Grooved terrain on Ganymede. Icarus 44(2):481–501. 10.1016/0019-1035(80)90039-1 10.1016/0019-1035(80)90039-1

[CR174] Luttrell K, Sandwell D (2006) Strength of the lithosphere of the Galilean satellites. Icarus 183(1):159–167. 10.1016/j.icarus.2006.01.015 10.1016/j.icarus.2006.01.015

[CR175] Makarov OP, Ajello JM, Vatti Palle P, Kanik I, Festou MC, Bhardwaj A (2004) Kinetic energy distributions and line profile measurements of dissociation products of water upon electron impact. J Geophys Res 109(A18):A09303. 10.1029/2002JA009353 10.1029/2002JA009353

[CR176] Manga M, Wang C-Y (2007) Pressurized oceans and the eruption of liquid water on Europa and Enceladus. Geophys Res Lett 34(7):L07202. 10.1029/2007GL029297 10.1029/2007GL029297

[CR177] Marconi ML (2007) A kinetic model of Ganymede’s atmosphere. Icarus 190(1):155–174. 10.1016/j.icarus.2007.02.016 10.1016/j.icarus.2007.02.016

[CR178] Mastrapa RM, Bernstein MP, Sandford SA, Roush TL, Cruikshank DP, Dalle Ore CM (2008) Optical constants of amorphous and crystalline H_2_O-ice in the near infrared from 1.1 to 2.6 μm. Icarus 197(1):307–320. 10.1016/j.icarus.2008.04.008 10.1016/j.icarus.2008.04.008

[CR179] McCord TB, Carlson RW, Smythe WD, Hansen GB, Clark RN, Hibbitts CA, Fanale FP, Granahan JC, Segura M, Matson DL, Johnson TV, Martin PD (1997) Organics and other molecules in the surfaces of Callisto and Ganymede. Science 278(5336):271–275. 10.1126/science.278.5336.271 9323203 10.1126/science.278.5336.271

[CR180] McCord TB, Hansen GB, Clark RN, Martin PD, Hibbitts CA, Fanale FP, Granahan JC, Segura M, Matson DL, Johnson TV, Carlson RW, Smythe WD, Danielson GE (the NIMS team) (1998a) Non-water ice constituents in the surface material of the icy Galilean satellites from the Galileo near-infrared mapping spectrometer investigation. J Geophys Res 103(E4):8603–8626. 10.1029/98JE00788 10.1029/98JE00788

[CR181] McCord TB, Hansen GB, Fanale FP, Carlson RW, Matson DL, Johnson TV, Smythe WD, Crowley JK, Martin PD, Ocampo A (1998b) Salts on Europa’s surface detected by Galileo’s near infrared mapping spectrometer. Science 280(5367):1242–1245. 10.1126/science.280.5367.1242 9596573 10.1126/science.280.5367.1242

[CR182] McCord TB, Hansen GB, Matson DL, Jonhson TV, Crowley JK, Fanale FP, Carlson RW, Smythe WD, Martin PD, Hibbitts CA (1999) Hydrated salt minerals on Europa’s surface from the Galileo Near-Infrared Mapping Spectrometer (NIMS) investigation. J Geophys Res 104(E5):11827–11852. 10.1029/1999JE900005 10.1029/1999JE900005

[CR183] McCord TB, Hansen GB, Hibbitts CA (2001b) Hydrated salt minerals on Ganymede’s surface: evidence of an ocean below. Science 292(5521):1523–1525. 10.1126/science.1059916 11375486 10.1126/science.1059916

[CR184] McCord TB, Orlando TM, Teeter G, Hansen GB, Sieger MT, Petrik NG, van Keulen L (2001a) Thermal and radiation stability of the hydrated salt minerals epsomite, mirabilite, and natron under Europa environmental conditions. J Geophys Res 106(E2):3311–3319. 10.1029/2000JE001282 10.1029/2000JE001282

[CR185] McCord TB, Teeter G, Hansen GB, Sieger MT, Orlando TM (2002) Brines exposed to Europa surface conditions. J Geophys Res 107(E1):4-1–4-6. 10.1029/2000JE001453 10.1029/2000JE001453

[CR186] McCord TB, Hansen GB, Combe J-P, Hayne P (2010) Hydrated minerals on Europa’s surface: an improved look from the Galileo NIMS investigation. Icarus 209(2):639–650. 10.1016/j.icarus.2010.05.026 10.1016/j.icarus.2010.05.026

[CR187] McEwen AS (1986) Tidal reorientation and the fracturing of Jupiter’s moon Europa. Nature 321(6065):49–51. 10.1038/321049a0 10.1038/321049a0

[CR188] McGrath MA, Hansen CJ, Hendrix AR (2009) Observations of Europa’s tenuous atmosphere. In: Pappalardo RT, McKinnon WB, Khurana KK (eds) Europa. University of Arizona Press, Tucson, pp 485–505

[CR189] McGrath MA, Jia X, Retherford K, Feldman PD, Strobel DF, Saur J (2013) Aurora on Ganymede. J Geophys Res Space Phys 118(5):2043–2054. 10.1002/jgra.50122 10.1002/jgra.50122

[CR190] McKinnon WB (1999) Convective instability in Europa’s floating ice shell. Geophys Res Lett 26(7):951–954. 10.1029/1999GL900125 10.1029/1999GL900125

[CR191] Melosh HJ (2012) Planetary surface processes. Cambridge University Press, Cambridge. 10.1017/CBO9780511977848

[CR192] Migliorini A, Kanuchova Z, Ioppolo S, Barbieri M, Jones NC, Hoffmann SV, Strazzulla G, Tosi F, Piccioni G (2022) On the origin of molecular oxygen on the surface of Ganymede. Icarus 383:115074. 10.1016/j.icarus.2022.115074 10.1016/j.icarus.2022.115074

[CR193] Mills MM, Pappalardo RT, Panning MP, Leonard EJ, Howell SM (2023) Moonquake-triggered mass wasting processes on icy satellites. Icarus 399:115534. 10.1016/j.icarus.2023.115534 10.1016/j.icarus.2023.115534

[CR194] Mitri G, Showman AP (2005) Convective conductive transitions and sensitivity of a convecting ice shell to perturbations in heat flux and tidal-heating rate: Implications for Europa. Icarus 177(2):447–460. 10.1016/j.icarus.2005.03.019 10.1016/j.icarus.2005.03.019

[CR195] Mitri G, Showman AP (2008) A model for the temperature-dependence of tidal dissipation in convective plumes on icy satellites: implications for Europa and Enceladus. Icarus 195(2):758–764. 10.1016/j.icarus.2008.01.010 10.1016/j.icarus.2008.01.010

[CR196] Molyneux PM, Nichols JD, Bannister NP, Bunce EJ, Clarke JT, Cowley SWH, Gérard J-C, Grodent D, Milan SE, Paty C (2018) Hubble Space Telescope observations of variations in Ganymede’s oxygen atmosphere and aurora. J Geophys Res Space Phys 123(5):3777–3793. 10.1029/2018JA025243 10.1029/2018JA025243

[CR197] Molyneux PM, Nichols JD, Becker TM, Raut U, Retherford KD (2020) Ganymede’s far-ultraviolet reflectance: constraining impurities in the surface ice. J Geophys Res, Planets 125(9):e2020JE006476. 10.1029/2020JE006476 10.1029/2020JE006476

[CR198] Molyneux PM, Greathouse TK, Gladstone GR, Versteeg MH, Hue V, Kammer J, Davis MW, Bolton SJ, Giles R, Connerney JEP, Gérard J-C, Grodent DC (2022) Ganymede’s UV reflectance from Juno UVS data. Geophys Res Lett 49(23):e2022GL099532. 10.1029/2022GL099532 10.1029/2022GL099532

[CR199] Moore JM, Mellon MT, Zent AP (1996) Mass wasting and ground collapse in terrains of volatile-rich deposits as a Solar System-wide geological process: the pre-Galileo view. Icarus 122(1):63–78. 10.1006/icar.1996.0109 10.1006/icar.1996.0109

[CR200] Moore JM, Asphaug E, Morrison D, Spencer JR, Chapman CR, Bierhaus B, Sullivan RJ, Chuang FC, Klemaszewski JE, Greeley R, Bender KC, Geissler PE, Helfenstein P, Pilcher CB (1999) Mass movement and landform degradation on the icy Galilean satellites: results of the Galileo nominal mission. Icarus 140(2):294–312. 10.1006/icar.1999.6132 10.1006/icar.1999.6132

[CR201] Moore JM, Chapman CR, Bierhaus EB, Greeley R, Chuang FC, Klemaszewski J, Clark RN, Dalton JB, Hibbitts CA, Schenk PM, Spencer JR, Wagner R (2004) Callisto. In: Bagenal F, Dowling TE, McKinnon WB (eds) Jupiter – the planet, satellites and magnetosphere. Cambridge University Press, Cambridge, pp 397–426

[CR202] Moroz VI (1965) Infrared spectrophotometry of the Moon and the Galilean satellites of Jupiter. Astronomicheskii Zhurnal 42(6):1287–1295

[CR203] Moroz LV, Arnold G, Korochantsev AV, Wäsch R (1998) Natural solid bitumens as possible analogs for cometary and asteroid organics: 1. Reflectance spectroscopy of pure bitumens. Icarus 134(2):253–268. 10.1006/icar.1998.5955 10.1006/icar.1998.5955

[CR204] Mura A, Adriani A, Sordini R, Sindoni S, Plainaki C, Tosi F, Filacchione G, Bolton S, Zambon F, Hansen CJ, Ciarniello M, Brooks S, Piccioni G, Grassi D, Altieri F, Migliorini A, Moriconi ML, Noschese R, Cicchetti A (2020) Infrared observations of Ganymede from the Jovian InfraRed auroral mapper on Juno. J Geophys Res, Planets 125(12):e06508. 10.1029/2020JE006508 10.1029/2020JE006508

[CR205] Murchie S, Head J, Plescia J (1990) Tectonic and volcanic evolution of dark terrain and its implications for the internal structure and evolution of Ganymede. J Geophys Res, Solid Earth 95(B7):10743–10768. 10.1029/JB095iB07p10743 10.1029/JB095iB07p10743

[CR206] Musacchio F, Saur J, Roth L, Retherford KD, McGrath MA, Feldman PD, Strobel DF (2017) Morphology of Ganymede’s FUV auroral ovals. J Geophys Res Space Phys 122(3):2855–2876. 10.1002/2016JA023220 10.1002/2016JA023220

[CR207] Nimmo F, Pappalardo RT (2004) Furrow flexure and ancient heat flux on Ganymede. Geophys Res Lett 31(19):L19701. 10.1029/2004GL020763 10.1029/2004GL020763

[CR208] Nimmo F, Pappalardo RT, Giese B (2003) On the origins of band topography, Europa. Icarus 166(1):21–32. 10.1016/j.icarus.2003.08.002 10.1016/j.icarus.2003.08.002

[CR209] Noll KS, Johnson RE, Lane AL, Domingue DL, Weaver HA (1996) Detection of ozone on Ganymede. Science 273(5273):341–343. 10.1126/science.273.5273.34 8662517 10.1126/science.273.5273.34

[CR210] Orton GS, Spencer JR, Travis LD, Martin TZ, Tamppari LK (1996) Galileo photopolarimeter-radiometer observations of Jupiter and the Galilean satellites. Science 274(5286):389–391. 10.1126/science.274.5286.389 10.1126/science.274.5286.389

[CR211] Oza AV, Johnson RE, Leblanc F (2018) Dusk/dawn atmospheric asymmetries on tidally-locked satellites: O_2_ at Europa. Icarus 305:50–55. 10.1016/j.icarus.2017.12.032 10.1016/j.icarus.2017.12.032

[CR212] Oza AV, Leblanc F, Johnson RE, Schmidt C, Leclercq L, Cassidy TA, Chaufray J-Y (2019) Dusk over dawn O_2_ asymmetry in Europa’s near-surface atmosphere. Planet Space Sci 167:23–32. 10.1016/j.pss.2019.01.006 10.1016/j.pss.2019.01.006

[CR213] Paganini L, Villanueva GL, Roth L, Mandell AM, Hurford TA, Retherford KD, Mumma MJ (2020) A measurement of water vapour amid a largely quiescent environment on Europa. Nat Astron 4(3):266–272. 10.1038/s41550-019-0933-6 10.1038/s41550-019-0933-6

[CR214] Palumbo P, Roatsch T, Lara LM, Castro JM, Della Corte V, Hviid S et al (2024) The JANUS (Jovis Amorum ac Natorum Undique Scrutator) VIS-NIR multi-band imager for the JUICE mission. Space Sci Rev 220

[CR215] Pappalardo RT, Barr AC (2004) The origin of domes on Europa: the role of thermally induced compositional diapirism. Geophys Res Lett 31(1):L01701. 10.1029/2003GL019202 10.1029/2003GL019202

[CR216] Pappalardo RT, Collins GC (2005) Strained craters on Ganymede. J Struct Geol 27:827–838. 10.1016/j.jsg.2004.11.010 10.1016/j.jsg.2004.11.010

[CR217] Pappalardo RT, Head JW, Greeley R, Sullivan RJ, Pilcher C, Schubert G, Moore WB, Carr MH, Moore JM, Belton MJS, Goldsby DL (1998) Geological evidence for solid-state convection in Europa’s ice shell. Nature 391(6665):365–368. 10.1038/34862 9450750 10.1038/34862

[CR218] Pappalardo RT, Collins GC, Head JW, Helfenstein P, McCord TB, Moore JM, Prockter LM, Schenk PM, Spencer J (2004) Geology of Ganymede. In: Bagenal F, Dowling TE, McKinnon WB (eds) Jupiter – the planet, satellites and magnetosphere. Cambridge University Press, Cambridge, pp 363–396

[CR219] Parekh R, Pappalardo RT, Scully JEC, Cameron ME (2023) Small-scale mass movements on Europa, Callisto and Ganymede. 54th Lunar and Planetary Science Conference 2023 (LPI Contrib. No. 2806). https://www.hou.usra.edu/meetings/lpsc2023/pdf/1876.pdf

[CR220] Parmentier EM, Head JW (1981) Viscous relaxation of impact craters on icy planetary surfaces: determination of viscosity variation with depth. Icarus 47(1):100–111. 10.1016/0019-1035(81)90095-6 10.1016/0019-1035(81)90095-6

[CR221] Parmentier EM, Squyres SW, Head JW, Allison ML (1982) The tectonics of Ganymede. Nature 295(5847):290–293. 10.1038/295290a0 10.1038/295290a0

[CR222] Patterson GW, Collins GC, Head JW, Pappalardo RT, Prockter LM, Lucchitta BK, Kay JP (2010) Global geological mapping of Ganymede. Icarus 207(2):845–867. 10.1016/j.icarus.2009.11.035 10.1016/j.icarus.2009.11.035

[CR223] Plainaki C, Milillo A, Mura A, Orsini S, Cassidy T (2010) Neutral particle release from Europa’s surface. Icarus 210(1):385–395. 10.1016/j.icarus.2010.06.041 10.1016/j.icarus.2010.06.041

[CR224] Plainaki C, Milillo A, Mura A, Orsini S, Massetti S, Cassidy T (2012) The role of sputtering and radiolysis in the generation of Europa exosphere. Icarus 218(2):956–966. 10.1016/j.icarus.2012.01.023 10.1016/j.icarus.2012.01.023

[CR225] Plainaki C, Milillo A, Massetti S, Mura A, Jia X, Orsini S, Mangano V, De Angelis E, Rispoli R (2015) The H_2_O and O_2_ exospheres of Ganymede: The result of a complex interaction between the Jovian magnetospheric ions and the icy moon. Icarus 245:306–319. 10.1016/j.icarus.2014.09.018 10.1016/j.icarus.2014.09.018

[CR226] Plainaki P, Cassidy TA, Shematovich VI, Milillo A, Wurz P, Vorburger A, Roth L, Galli A, Rubin M, Blöcker A, Brandt PC, Crary F, Dandouras I, Jia X, Grassi D, Hartogh P, Lucchetti A, McGrath M, Mangano V, Mura A (2018) Towards a global unified model of Europa’s tenuous atmosphere. Space Sci Rev 214(1):71. 10.1007/s11214-018-0469-6 10.1007/s11214-018-0469-6

[CR227] Plainaki C, Massetti S, Jia X, Mura A, Milillo A, Grassi D, Sindoni G, D’Aversa E, Filacchione G (2020a) Kinetic simulations of the Jovian energetic ion circulation around Ganymede. Astrophys J 900(1):74. 10.3847/1538-4357/aba94c 10.3847/1538-4357/aba94c

[CR228] Plainaki C, Sindoni G, Grassi D, Cafarelli L, D’Aversa E, Massetti S, Mura A, Milillo A, Filacchione G, Piccioni G, Langevin Y, Poulet F, Tosi F, Migliorini A, Altieri F (2020b) Preliminary estimation of the detection possibilities of Ganymede’s water vapor environment with MAJIS. Planet Space Sci 191:105004. 10.1016/j.pss.2020.105004 10.1016/j.pss.2020.105004

[CR229] Poppe AR, Fatemi S, Khurana KK (2018) Thermal and energetic ion dynamics in Ganymede’s magnetosphere. J Geophys Res Space Phys 123(6):4614–4637. 10.1029/2018JA025312 10.1029/2018JA025312

[CR230] Porco CC, Helfenstein P, Thomas PC, Ingersoll AP, Wisdom J, West R, Neukum G, Denk T, Wagner R, Roatsch T, Kieffer S, Turtle E, McEwen A, Johnson TV, Rathbun J, Veverka J, Wilson D, Perry J, Spitale J, Brahic A, Burns JA, Del Genio AD, Dones L, Murray CD, Squyres S (2006) Cassini observes the active south pole of Enceladus. Science 311(5766):1393–1401. 10.1126/science.11230 16527964 10.1126/science.11230

[CR231] Porco C, DiNino D, Nimmo F (2014) How the geysers, tidal stresses, and thermal emission across the south polar terrain of Enceladus are related. Astron J 148(3):45. 10.1088/0004-6256/148/3/45 10.1088/0004-6256/148/3/45

[CR232] Postberg F, Kempf S, Schmidt J, Brilliantov N, Beinsen A, Abel B, Buck U, Srama R (2009) Sodium salts in E-ring ice grains from an ocean below the surface of Enceladus. Nature 459(7250):1098–1101. 10.1038/nature08046 19553992 10.1038/nature08046

[CR233] Postberg F, Grün E, Horanyi M, Kempf S, Krüger H, Schmidt J, Spahn F, Srama R, Sternovsky Z, Trieloff M (2011b) Compositional mapping of planetary moons by mass spectrometry of dust ejecta. Planet Space Sci 59(14):1815–1825. 10.1016/j.pss.2011.05.001 10.1016/j.pss.2011.05.001

[CR234] Postberg F, Schmidt J, Hillier JK, Kempf S, Srama R (2011a) A salt-water reservoir as the source of a compositionally stratified plume on Enceladus. Nature 474(7353):620–622. 10.1038/nature10175 21697830 10.1038/nature10175

[CR235] Poulet F, Piccioni G, Langevin Y, Dumesnil C, Tommasi L, Carlier V, Filacchione G, Amoroso M, Arondel A, D’Aversa E, Barbis A, Bini A, Bolsée D, Bousquet P, Caprini C, Carter J, Dubois J-P, Condamin M, Couturier S, Dassas K, Dexet M, Fletcher L, Grassi D, Guerri I, Haffoud P, Larigauderie C, Du Le M, Mugnuolo R, Pilato G, Rossi M, Stefani S, Tosi F, Vincendon M, Zambelli M, Arnold G, Bibring J-P, Biondi D, Boccaccini A, Brunetto R, Carapelle A, Cisneros González M, Hannou C, Karatekin O, Le Cle’h J-C, Leyrat C, Migliorini A, Nathues A, Rodriguez S, Saggin B, Sanchez-Lavega A, Schmitt B, Seignovert B, Sordini R, Stephan K, Tobie G, Zambon F, Adriani A, Altieri F, Bockelée-Morvan D, Capaccioni F, De Angelis S, De Sanctis M-C, Drossart P, Fouchet T, Gérard J-C, Grodent D, Ignatiev N, Irwin P, Ligier N, Manaud N, Mangold N, Mura A, Pilorget C, Quirico E, Renotte E, Strazzulla G, Turrini D, Vandaele A-C, Carli C, Ciarniello M, Guerlet S, Lellouch E, Mancarella F, Morbidelli A, Le Mouélic S, Raponi A, Sindoni G, Snels M (2024) Moons And Jupiter Imaging Spectrometer (MAJIS) on Jupiter Icy Moons Explorer (JUICE). Space Sci Rev 220(3):27. 10.1007/s11214-024-01057-2 10.1007/s11214-024-01057-2

[CR236] Prockter LM, Pappalardo RT (2014) Europa. In: Spohn T, Breuer D, Johnson TV (eds) Encyclopedia of the Solar System, 3rd edn. Elsevier, Amsterdam, pp 793–811. 10.1016/B978-0-12-415845-0.00036-0

[CR237] Prockter L, Figueredo P, Pappalardo R, Head J (2000) Geology and mapping of dark terrain on Ganymede and implications for grooved terrain formation. J Geophys Res, Planets 105(E9):22519–22540. 10.1029/1999JE001179 10.1029/1999JE001179

[CR238] Prockter LM, Lopes RMC, Giese B, Jaumann R, Lorenz RD, Pappalardo RT, Patterson GW, Thomas PC, Turtle EP, Wagner RJ (2010) Characteristics of icy surfaces. Space Sci Rev 153(1–4):63–111. 10.1007/s11214-010-9649-8 10.1007/s11214-010-9649-8

[CR239] Quick LC, Hedman MM (2020) Characterizing deposits emplaced by cryovolcanic plumes on Europa. Icarus 343:113667. 10.1016/j.icarus.2020.113667 10.1016/j.icarus.2020.113667

[CR240] Quick LC, Barnouin OS, Prockter LM, Patterson GW (2013) Constraints on the detection of cryovolcanic plumes on Europa. Planet Space Sci 86:1–9. 10.1016/j.pss.2013.06.028 10.1016/j.pss.2013.06.028

[CR241] Ravine MA, Hansen CJ, Collins GC, Schenk PM, Caplinger MA, Lipkaman Vittling L, Krysak DJ, Zimdar RP, Garvin JB, Bolton SJ (2022) Ganymede observations by JunoCam on Juno Perijove 34. Geophys Res Lett 49(23):e2022GL099211. 10.1029/2022GL099211 10.1029/2022GL099211 PMC10078141 37034393

[CR242] Retherford KD, Gladstone GR, Persyn SC, Davis MW, Greathouse TK, Molyneux PM et al (2024) The Ultraviolet Spectrograph on ESA’s Jupiter Icy Moons Explorer Mission (JUICE-UVS). Space Sci Rev 220

[CR243] Reynolds RT, Squyres SW, Colburn DS, McKay CP (1983) On the habitability of Europa. Icarus 56(2):246–254. 10.1016/0019-1035(83)90037-4 10.1016/0019-1035(83)90037-4

[CR244] Rossi C, Cianfarra P, Salvini F, Mitri G, Massé M (2018) Evidence of transpressional tectonics on the Uruk Sulcus region, Ganymede. Tectonophysics 749:72–87. 10.1016/j.tecto.2018.10.026 10.1016/j.tecto.2018.10.026

[CR245] Rossi C, Lucchetti A, Massironi M, Penasa L, Pozzobon R, Munaretto G, Pajola M (2023) Multi-phase activity on Ganymede’s dark terrain: tectonic evolution of Galileo regio. Icarus 390:115305. 10.1016/j.icarus.2022.115305 10.1016/j.icarus.2022.115305

[CR246] Roth L (2021) A stable H_2_O atmosphere on Europa’s trailing hemisphere from HST images. Geophys Res Lett 48(20):e2021GL094289. 10.1029/2021GL094289 10.1029/2021GL094289

[CR247] Roth L, Retherford KD, Saur J, Strobel DF, Feldman PD, McGrath MA, Nimmo F (2014b) Orbital apocenter is not a sufficient condition for HST/STIS detection of Europa’s water vapor atmosphere. Proc Natl Acad Sci USA 111(48):E5123–E5132. 10.1073/pnas.1416671111 25404343 10.1073/pnas.1416671111 PMC4260579

[CR248] Roth L, Saur J, Retherford KD, Strobel DF, Feldman PD, McGrath MA, Nimmo F (2014a) Transient water vapor at Europa’s south pole. Science 343(6167):171–174. 10.1126/science.1247051 24336567 10.1126/science.1247051

[CR249] Roth L, Saur J, Retherford KD, Strobel DF, Feldman PD, McGrath MA, Spencer JR, Blöcker A, Ivchenko N (2016) Europa’s far ultraviolet oxygen aurora from a comprehensive set of HST observations. J Geophys Res Space Phys 121(3):2143–2170. 10.1002/2015JA022073 10.1002/2015JA022073

[CR250] Roth L, Alday J, Becker TM, Ivchenko N, Retherford KD (2017b) Detection of a hydrogen corona at Callisto. J Geophys Res, Planets 122(5):1046–1055. 10.1002/2017JE005294 10.1002/2017JE005294

[CR251] Roth L, Retherford K, Ivchenko N, Schlatter N, Strobel DF, Becker TM, Grava C (2017a) Detection of a hydrogen corona in HST Ly images of Europa in transit of Jupiter. Astron J 153(2):67. 10.3847/1538-3881/153/2/67 10.3847/1538-3881/153/2/67

[CR252] Roth L, Ivchenko N, Gladstone GR, Saur J, Grodent D, Bonfond B, Molyneux PM, Retherford KD (2021) A sublimated water atmosphere on Ganymede detected from Hubble Space Telescope observations. Nat Astron 5(10):1043–1051. 10.1038/s41550-021-01426-9 10.1038/s41550-021-01426-9

[CR253] Roth L, Marchesini G, Becker TM, Hoeijmakers HJ, Molyneux PM, Retherford KD, Saur J, Carberry Mogan SR, Szalay JR (2023) Probing Ganymede’s atmosphere with HST Ly images in transit of Jupiter. Planet Sci J 4(1):12. 10.3847/PSJ/acaf7f 10.3847/PSJ/acaf7f

[CR254] Roush TL, Pollack JB, Witteborn FC, Bregman JD, Simpson JP (1990) Ice and minerals on Callisto: a reassessment of the reflectance spectra. Icarus 86(2):355–382. 10.1016/0019-1035(90)90225-X 10.1016/0019-1035(90)90225-X

[CR255] Saur J, Duling S, Roth L, Jia X, Strobel DF, Feldman PD, Christensen UR, Retherford KD, McGrath MA, Musacchio F, Wennmacher A, Neubauer FM, Simon S, Hartkorn O (2015) The search for a subsurface ocean in Ganymede with Hubble Space Telescope observations of its auroral ovals. J Geophys Res Space Phys 120(3):1715–1737. 10.1002/2014ja020778 10.1002/2014ja020778

[CR256] Schenk P (2002) Thickness constraints on the icy shells of the Galilean satellites from a comparison of crater shapes. Nature 417(6887):419–421. 10.1038/417419a 12024207 10.1038/417419a

[CR257] Schenk PM (2024) Revised cartographic and topographic data of the Galilean satellites from Voyager, Galileo, New Horizons and Juno. 55th Lunar and Planetary Science Conference, held 11-15 March, 2024 at the Woodlands, Texas/Virtual. LPI Contribution No. 3040, id.2687. https://www.hou.usra.edu/meetings/lpsc2024/pdf/2687.pdf

[CR258] Schenk PM, Moore JM (1995) Volcanic constructs on Ganymede and Enceladus: topographic evidence from stereo images and photoclinometry. J Geophys Res 100(E9):19009–19022. 10.1029/95JE01854 10.1029/95JE01854

[CR259] Schenk PM, Asphaug E, McKinnon WB, Melosh HJ, Weissman PR (1996) Cometary nuclei and tidal disruption: the geological record of crater chains on Callisto and Ganymede. Icarus 121(2):249–274. 10.1006/icar.1996.0084 10.1006/icar.1996.0084

[CR260] Schenk PM, McKinnon WB, Gwynn D, Moore JM (2001) Flooding of Ganymede’s bright terrains by low-viscosity water-ice lavas. Nature 410(6824):57–60. 10.1038/35065027 11242037 10.1038/35065027

[CR261] Schenk PM, Chapman CR, Zahnle K, Moore JM (2004) Ages and interiors: the cratering record of the Galilean satellites. In: Bagenal F, Dowling TE, McKinnon WB (eds) Jupiter: the planet, satellites and magnetosphere. Cambridge University Press, Cambridge, pp 427–456

[CR262] Schenk P, McKinnon WB, Moore J, Nimmo F (2021) The topography of Ganymede (and Callisto): Geology, global characteristics, and future exploration. 52nd Lunar and Planetary Science Conference, held virtually, 15-19 March, 2021. LPI Contribution No. 2548, id.2228. https://www.hou.usra.edu/meetings/lpsc2021/pdf/2228.pdf

[CR263] Schmidt BE, Blankenship DD, Patterson GW, Schenk PM (2011) Active formation of ‘chaos terrain’ over shallow subsurface water on Europa. Nature 479(7374):502–505. 10.1038/nature10608 22089135 10.1038/nature10608

[CR264] Schultz RA, Okubo CH, Wilkins SJ (2006) Displacement-length scaling relations for faults on the terrestrial planets. J Struct Geol 28:2182–2193. 10.1016/j.jsg.2006.03.034 10.1016/j.jsg.2006.03.034

[CR265] Schultz RA, Hauber E, Kattenhorn SA, Okubo CH, Watters T (2010a) Interpretation and analysis of planetary structures. J Struct Geol 32:855–875. 10.1016/j.jsg.2009.09.005 10.1016/j.jsg.2009.09.005

[CR266] Schultz RA, Soliva R, Okubo CH, Mège D (2010b) Fault populations. In: Watters TR, Schultz RA (eds) Planetary tectonics. Cambridge University Press, Cambridge, pp 457–510. 10.1017/CBO9780511691645.011

[CR267] Senft LE, Stewart ST (2011) Modeling the morphological diversity of impact craters on icy satellites. Icarus 214(1):67–81. 10.1016/j.icarus.2011.04.015 10.1016/j.icarus.2011.04.015

[CR268] Shematovich VI (2016) Neutral atmosphere near the icy surface of Jupiter’s moon Ganymede. Sol Syst Res 50(4):262–280. 10.1134/S0038094616040067 10.1134/S0038094616040067

[CR269] Shematovich VI, Johnson RE (2001) Near-surface oxygen atmosphere of Europa. Adv Space Res 27(11):1881–1888. 10.1016/S0273-1177(01)00299-X 10.1016/S0273-1177(01)00299-X

[CR270] Shematovich VI, Johnson RE, Cooper JF, Wong MC (2005) Surface-bounded atmosphere of Europa. Icarus 173(2):480–498. 10.1016/j.icarus.2004.08.013 10.1016/j.icarus.2004.08.013

[CR271] Shirley JH, Dalton JB, Prockter LM, Kamp LW (2010) Europa’s ridged plains and smooth low albedo plains: distinctive compositions and compositional gradients at the leading side-trailing side boundary. Icarus 210(1):358–384. 10.1016/j.icarus.2010.06.018 10.1016/j.icarus.2010.06.018

[CR272] Simpson RA, Tyler LG (1981) Viking bistatic radar experiment: Summary of first-order results emphasizing north polar data. Icarus 46(3):361–389. 10.1016/0019-1035(81)90139-1 10.1016/0019-1035(81)90139-1

[CR273] Simpson RA, Tyler LG (1991) Surface properties of Galilean satellites from bistatic radar experiments. NASA, Washington, Reports of Planetary Geology and Geophysics Program, 1990. https://ntrs.nasa.gov/citations/19920001631

[CR274] Simpson RA (1993) Spacecraft studies of planetary surfaces using bistatic radar. Remote Sens 31(2):465–482. 10.1109/36.214923 10.1109/36.214923

[CR275] Simpson RA, Tyler LG, Pätzold M, Häusler B, Asmar SW, Sultan-Salem AK (2011) Polarization in bistatic radar probing of planetary surfaces: Application to Mars Express data. Proceedings of the IEEE 99(5):858–874. 10.1109/JPROC.2011.2106190 10.1109/JPROC.2011.2106190

[CR276] Singer KN, McKinnon WB, Schenk PM, Moore JM (2012) Massive ice avalanches on Iapetus mobilized by friction reduction during flash heating. Nat Geosci 5(8):574–578. 10.1038/ngeo1526 10.1038/ngeo1526

[CR277] Singer KN, Bland MT, Schenk PM, McKinnon WB (2018) Relaxed impact craters on Ganymede: regional variation and high heat flows. Icarus 306:214–224. 10.1016/j.icarus.2018.01.012 10.1016/j.icarus.2018.01.012

[CR278] Smith HT, Mitchell DG, Johnson RE, Mauk BH, Smith JE (2019) Europa neutral torus confirmation and characterization based on observations and modeling. Astrophys J 871(1):692019. 10.3847/1538-4357/aaed38 10.3847/1538-4357/aaed38

[CR279] Smyth WH, Marconi ML (2006) Europa’s atmosphere, gas tori, and magnetospheric implications. Icarus 181(2):510–526. 10.1016/j.icarus.2005.10.019 10.1016/j.icarus.2005.10.019

[CR280] Soderblom LA, Kieffer SW, Becker TL, Brown RH, Cook AF, Hansen CJ, Johnson TV, Kirk RL, Shoemaker EM (1990) Triton’s geyser-like plumes: discovery and basic characterization. Science 250(4979):410–415. 10.1126/science.250.4979.410 17793016 10.1126/science.250.4979.410

[CR281] Solomonidou A, Stephan K, Kalousova K, Soderlund K (2021) Candidate cryovolcanic regions on Ganymede: a target priority for JUICE. EPSC Abstracts 15:EPSC2021-81. 10.5194/epsc2021-81

[CR282] Sotin C, Head JW, Tobie G (2002) Europa: tidal heating of upwelling thermal plumes and the origin of lenticulae and chaos melting. Geophys Res Lett 29(8):1233. 10.1029/2001GL013844. 74-1–74-4 10.1029/2001GL013844

[CR283] Sparks WB, Hand KP, McGrath MA, Bergeron E, Cracraft M, Deustua SE (2016) Probing for evidence of plumes on Europa with HST/STIS. Astrophys J 829(2):121. 10.3847/0004-637X/829/2/121 10.3847/0004-637X/829/2/121

[CR284] Sparks WB, Schmidt BE, McGrath MA, Hand KP, Spencer JR, Cracraft M, Deustua SE (2017) Active cryovolcanism on Europa? Astrophys J Lett 839(2):L18. 10.3847/2041-8213/aa67f8 10.3847/2041-8213/aa67f8

[CR285] Sparks WB, Richter M, deWitt C, Montiel E, Dello Russo N, Grunsfeld JM, McGrath MA, Weaver H, Hand KP, Bergeron E, Reach W (2019) A search for water vapor plumes on Europa using SOFIA. Astrophys J 871(1):L5. 10.3847/2041-8213/aafb0a 10.3847/2041-8213/aafb0a

[CR286] Spaun NA, Head JW III, Pappalardo RT (Galileo SSI Team) (2001) Scalloped depressions on Ganymede from Galileo (G28) very high resolution imaging. In: Lunar and Planetary Science Conference, vol. 32, p. 1448

[CR287] Spencer JR (1987) Thermal segregation of water ice on the Galilean satellites. Icarus 69(2):297–313. 10.1016/0019-1035(87)90107-2 10.1016/0019-1035(87)90107-2

[CR288] Spencer JR, Calvin WM, Person MJ (1995) CCD spectra of the Galilean satellites: molecular oxygen on Ganymede. J Geophys Res 100(E9):19049–19056. 10.1029/95JE01503 10.1029/95JE01503

[CR289] Spitale J, Porco C (2007) Association of the jets of Enceladus with the warmest regions on its south-polar fractures. Nature 449(7163):695–697. 10.1038/nature06217 17928854 10.1038/nature06217

[CR290] Stephan K, Jaumann R, Wagner R (2013) Geology of icy bodies. In: Gudipati MS, Castillo-Rogez J (eds) The science of Solar System ices. Springer, New York, pp 279–367. 10.1007/978-1-4614-3076-6_10

[CR291] Stephan K, Hibbitts CA, Jaumann R (2020) H_2_O-ice particle size variations across Ganymede’s and Callisto’s surface. Icarus 337:113440. 10.1016/j.icarus.2019.113440 10.1016/j.icarus.2019.113440

[CR292] Stephan K, Ciarniello M, Poch O, Schmitt B, Haack D, Raponi A (2021b) VIS-NIR/SWIR spectral properties of H_2_O ice depending on particle size and surface temperature. Minerals 11(12):1328. 10.3390/min11121328 10.3390/min11121328

[CR293] Stephan K, Roatsch T, Tosi F, Matz K-D, Kersten E, Wagner R, Molyneux P, Palumbo P, Poulet F, Hussmann H, Barabash S, Bruzzone L, Dougherty M, Gladstone R, Gurvits LI, Hartogh P, Iess L, Wahlund J-E, Wurz P, Witasse O, Grasset O, Altobelli N, Carter J, Cavalié T, D’Aversa E, Della Corte V, Filacchione G, Galli A, Galluzzi V, Gwinner K, Hauber E, Jaumann R, Krohn K, Langevin Y, Lucchetti A, Piccioni G, Solomonidou A, Stark A, Tobie G, Tubiana C, Vallat C, Van Hoolst T (the JUICE SWT Team) (2021a) Regions of interest on Ganymede’s and Callisto’s surfaces as potential targets for ESA’s JUICE mission. Planet Space Sci 208:105324. 10.1016/j.pss.2021.105324 10.1016/j.pss.2021.105324

[CR294] Strobel DF, Saur J, Feldman PD, McGrath MA (2002) Hubble Space Telescope space telescope imaging spectrograph search for an atmosphere on Callisto: a Jovian unipolar inductor. Astrophys J 581(1):L51–L54. 10.1086/345803 10.1086/345803

[CR295] Szalay JR, Smith HT, Zirnstein EJ, McComas DJ, Begley LJ, Bagenal F, Delamere PA, Wilson RJ, Valek PW, Poppe AR, Nénon Q, Allegrini F, Ebert RW, Bolton SJ (2022) Water-group pickup ions from Europa-genic neutrals orbiting Jupiter. Geophys Res Lett 49(9):e2022GL098111. 10.1029/2022GL098111 10.1029/2022GL098111 PMC9286426 35864892

[CR296] Szalay JR, Allegrini F, Ebert RW, Bagenal F, Bolton SJ, Fatemi S, McComas DJ, Pontoni A, Saur J, Smith HT, Strobel DF, Vance SD, Vorburger A, Wilson RJ (2024) Oxygen production from dissociation of Europa’s water-ice surface. Nat Astron 8(5):567–576. 10.1038/s41550-024-02206-x 38798715 10.1038/s41550-024-02206-x PMC11111413

[CR297] Taylor J, Kar-Ming C, Seo D (2002) Galileo Telecommunications final report. JPL DESCANSO (Deep Space Communications and Navigation Systems Center of Excellence), Design and Performances Summary Series. https://descanso.jpl.nasa.gov/DPSummary/Descanso5--Galileo_new.pdf

[CR298] Teolis BD, Plainaki C, Cassidy TA, Raut U (2017a) Water ice radiolytic O_2_, H_2_, and H_2_O_2_ yields for any projectile species, energy, or temperature: a model for icy astrophysical bodies. J Geophys Res, Planets 122(10):1996–2012. 10.1002/2017JE005285 10.1002/2017JE005285

[CR299] Teolis BD, Wyrick DY, Bouquet A, Magee BA, Waite JH (2017b) Plume and surface feature structure and compositional effects on Europa’s global exosphere: preliminary Europa mission predictions. Icarus 284:18–29. 10.1016/j.icarus.2016.10.027 10.1016/j.icarus.2016.10.027

[CR300] Thompson WR, Squyres SW (1990) Titan and other icy satellites: Dielectric properties of constituent materials and implications for radar sounding. Icarus 86(2):336–354. 10.1016/0019-1035(90)90224-W 10.1016/0019-1035(90)90224-W

[CR301] Tobie G, Giese B, Hurford TA, Lopes RM, Nimmo F, Postberg F, Retherford KD, Schmidt J, Spencer JR, Tokano T, Turtle EP (2010) Surface, subsurface and atmosphere exchanges on the satellites of the outer Solar System. Space Sci Rev 153:375–410. 10.1007/s11214-010-9641-3 10.1007/s11214-010-9641-3

[CR302] Tosi F, Turrini D, Coradini A, Filacchione G (2010) Probing the origin of the dark material on Iapetus. Mon Not R Astron Soc 403(3):1113–1130. 10.1111/j.1365-2966.2010.16044.x 10.1111/j.1365-2966.2010.16044.x

[CR303] Tosi F, Galluzzi V, Lucchetti A, Orosei R, Filacchione G, Zambon F, Cremonese G, Palumbo P, Piccioni G (2023) Mutidisciplinary analysis of the Nippur Sulcus region on Ganymede. J Geophys Res, Planets 128(7):e2023JE007836. 10.1029/2023JE007836 10.1029/2023JE007836

[CR304] Tosi F, Mura A, Cofano A, Zambon F, Glein CR, Ciarniello M, Lunine JI, Piccioni G, Plainaki C, Sordini R, Adriani A, Bolton SJ, Hansen CJ, Nordheim TA, Moirano A, Agostini L, Altieri F, Brooks SM, Cicchetti A, Dinelli BM, Grassi D, Migliorini A, Moriconi ML, Noschese R, Scarica P, Sindoni G, Stefani S, Turrini D (2024) Salts and organics on Ganymede’s surface observed by the JIRAM spectrometer onboard Juno. Nat Astron 8(1):82–93. 10.1038/s41550-023-02107-5 10.1038/s41550-023-02107-5

[CR305] Trumbo SK, Brown ME (2023) The distribution of CO2 on Europa indicates an internal source of carbon. Science 381(6664):1308–1311. 10.1126/science.adg4155 37733851 10.1126/science.adg4155

[CR306] Trumbo SK, Brown ME, Hand KP (2019a) Sodium chloride on the surface of Europa. Sci Adv 5(6):aaw7123. 10.1126/sciadv.aaw7123 10.1126/sciadv.aaw7123 PMC6561749 31206026

[CR307] Trumbo SK, Brown ME, Hand KP (2019b) H_2_O_2_ within chaos terrain on Europa’s leading hemisphere. Astron J 158(3):127. 10.3847/1538-3881/ab380c 10.3847/1538-3881/ab380c

[CR308] Trumbo SK, Becker TM, Brown ME, Denman WTP, Molyneux P, Hendrix A, Retherford KD, Roth L, Alday J (2022) A new UV spectral feature on Europa: confirmation of NaCl in leading-hemisphere chaos terrain. Planet Sci J 3(2):27. 10.3847/PSJ/ac4580 10.3847/PSJ/ac4580

[CR309] Trumbo SK, Brown ME, Bockelée-Morvan D, de Pater I, Fouchet T, Wong MH, Cazaux S, Fletcher LN, de Kleer K, Lellouch E, Mura A, Poch O, Quirico E, Rodriguez-Ovalle P, Showalter MR, Tiscareno MR, Tosi F (2023) Hydrogen peroxide at the poles of Ganymede. Sci Adv 9(29):eadg3724. 10.1126/sciadv.adg3724 37478185 10.1126/sciadv.adg3724 PMC10361591

[CR310] Tufts BR, Greenberg R, Hoppa GV, Geissler P (1999) Astypalaea Linea: a San Andreas-sized strike-slip fault on Europa. Icarus 141(1):53–64. 10.1006/icar.1999.6168 10.1006/icar.1999.6168

[CR311] Turcotte DL, Schubert G (2002) Geodynamics, 2nd edn. Cambridge University Press, Cambridge. 10.1017/CBO9780511807442

[CR312] Van Hoolst T, Tobie G, Vallat C, Altobelli N, Bruzzone L, Cao H, Iess L, Kimura J, Khurana K, Dirkx D, Genova A, Hussmann H, Lucchetti A, Mitri G, Moore W, Saur J, Stark A, Vorburger A, Wieczorek M, Aboudan A, Bergman J, Bovolo F, Breuer D, Cappuccio P, Carrer L, Cecconi B, Choblet G, De Marchi F, Fayolle M, Fienga A, Futaana Y, Hauber E, Kofman W, Kumamoto A, Lainey V, Molyneux P, Mousis O, Plaut J, Puccio W, Retherford K, Roth L, Seignovert B, Steinbrügge G, Thakur S, Tortora P, Tosi F, Zannoni M, Barabash S, Dougherty M, Gladstone R, Gurvits LI, Hartogh P, Palumbo P, Poulet F, Wahlund J-F, Grasset O, Witasse O (2024) Geophysical characterization of the interiors of Ganymede, Callisto and Europa by ESA’s JUpiter ICy moons Explorer. Space Sci Rev 220:54. 10.1007/s11214-024-01085-y 10.1007/s11214-024-01085-y

[CR313] Vance S, Bouffard M, Choukroun M, Sotin C (2014) Ganymede’s internal structure including thermodynamics of magnesium sulfate oceans in contact with ice. Planet Space Sci 96:62–70. 10.1016/j.pss.2014.03.011 10.1016/j.pss.2014.03.011

[CR314] Villanueva GL, Hammel HB, Milam SN, Faggi S, Kofman V, Roth L, Hand KP, Paganini L, Stansberry J, Spencer J, Protopapa S, Strazzulla G, Cruz-Mermy G, Glein CR, Cartwright R, Liuzzi G (2023) Endogenous CO2 ice mixture on the surface of Europa and no detection of plume activity. Science 381(6664):1305–1308. 10.1126/science.adg4270 37733858 10.1126/science.adg4270

[CR315] Vorburger A, Wurz P (2018) Europa’s ice-related atmosphere: the sputter contribution. Icarus 311:135–145. 10.1016/j.icarus.2018.03.022 10.1016/j.icarus.2018.03.022

[CR316] Vorburger A, Wurz P, Lammer H, Barabash S, Mousis O (2015) Monte-Carlo simulation of Callisto’s exosphere. Icarus 262:14–29. 10.1016/j.icarus.2015.07.035 10.1016/j.icarus.2015.07.035

[CR317] Vorburger A, Pfleger M, Lindkvist J, Holmström M, Lammer H, Lichtenegger HIM, Wurz P (2019) 3D-modeling of Callisto’s surface sputtered exosphere environment. J Geophys Res Space Phys 124(8):7151–7169. 10.1029/2019JA026610 10.1029/2019JA026610

[CR318] Vorburger A, Fatemi S, Galli A, Liuzzo L, Poppe AR, Wurz P (2023) 3D Monte-Carlo simulation of Ganymede’s water exosphere. Icarus 375:114810. 10.1016/j.icarus.2021.114810 10.1016/j.icarus.2021.114810

[CR319] Wagner RJ, Schmedemann N, Neukum G, Werner SC, Ivanov BA, Stephan K, Jaumann R, Palumbo P (2014) Crater size distributions on the Jovian satellites Ganymede and Callisto: Reassessment of Galileo and Voyager images, and an outlook to ESA’s JUICE mission. EPSC Abstracts 9:EPSC2014-551 https://meetingorganizer.copernicus.org/EPSC2014/EPSC2014-551.pdf

[CR320] Winterhalder TO, Huybrighs HLF (2022) Assessing JUICE’s ability of in situ plume detection in Europa’s atmosphere. Planet Space Sci 210:105375. 10.1016/j.pss.2021.105375 10.1016/j.pss.2021.105375

[CR321] Wirström ES, Bjerkeli P, Rezac L, Brinch C, Hartogh P (2020) Effect of the 3D distribution on water observations made with the SWI I. Ganymede. Astron Astrophys 637:A90. 10.1051/0004-6361/202037609 10.1051/0004-6361/202037609

[CR322] Yung YL, McElroy MB (1977) Stability of an oxygen atmosphere on Ganymede. Icarus 30(1):97–103. 10.1016/0019-1035(77)90124-5 10.1016/0019-1035(77)90124-5

[CR323] Zimmer C, Khurana KK, Kivelson MG (2000) Subsurface oceans on Europa and Callisto: constraints from Galileo magnetometer observations. Icarus 147(2):329–347. 10.1006/icar.2000.6456 10.1006/icar.2000.6456

[CR324] Zubarev A, Nadezhdina I, Brusnikin E, Giese B, Oberst J (2017) A search for Ganymede stereo images and 3D mapping opportunities. Planet Space Sci 146:40–54. 10.1016/j.pss.2017.07.021 10.1016/j.pss.2017.07.021

